# Extracellular matrix dysregulation in aging, calcification, and cancer diseases: insights into cellular senescence, inflammation, and novel therapeutic strategies

**DOI:** 10.7150/ijbs.119301

**Published:** 2025-10-24

**Authors:** Diego Liviu Boaru, Diego De Leon-Oliva, Patricia De Castro-Martinez, Cielo Garcia-Montero, Oscar Fraile-Martinez, Beatriz García-González, Isabel Pérez-González, Majd N. Michael Alhaddadin, Silvestra Barrena-Blázquez, Laura Lopez-Gonzalez, Basilio de la Torre, Leonel Pekarek, Miguel A Saez, Laura Ríos-Espinosa, Tatiana Pekarek, Roberto Fernández-Baillo Gallego de la Sacristana, Mauricio Hernández-Fernández, Carlos Casanova, Ana Castel-Oñate, Natalio Garcia-Honduvilla, Julia Buján, Raul Diaz-Pedrero, Melchor Alvarez-Mon, Miguel A Ortega

**Affiliations:** 1 Department of Medicine and Medical Specialities, Faculty of Medicine and Health Sciences, Network Biomedical Research Center for Liver and Digestive Diseases (CIBEREHD), University of Alcalá, 28801 Alcala de Henares, Spain.; 2 Ramón y Cajal Institute of Sanitary Research (IRYCIS), 28034 Madrid, Spain.; 3 Department of Nursing and Physiotherapy, Faculty of Medicine and Health Sciences, University of Alcalá, 28801 Alcala de Henares, Spain.; 4 Department of Surgery, Medical and Social Sciences, Faculty of Medicine and Health Sciences, University of Alcalá, 28801 Alcala de Henares, Spain.; 5 Oncology Service, Guadalajara University Hospital, 19002 Guadalajara, Spain.; 6 Pathological Anatomy Service, Central University Hospital of Defence-UAH Madrid, 28801 Alcala de Henares, Spain.; 7 Department of General and Digestive Surgery, Príncipe de Asturias, University Hospital, 28805 Alcala de Henares, Spain.; 8 Immune System Diseases-Rheumatology, Oncology Service an Internal Medicine (CIBEREHD), University Hospital Príncipe de Asturias, 28806 Alcala de Henares, Spain.

**Keywords:** extracellular matrix, aging, calcification, cancer, cellular senescence, chronic inflammation, extracellular vesicles, tumor microenvironment, collagen, and metalloproteases

## Abstract

This review underscores the dynamic role of the extracellular matrix (ECM) in regulating cellular behavior and maintaining tissue homeostasis, highlighting its pivotal involvement in aging, calcification, and cancer diseases. In healthy tissues, controlled ECM remodeling provides essential biochemical and mechanical cues, but dysregulation (driven by chronic inflammation, cellular senescence, and altered intercellular communication) leads to fibrosis, calcification, and the creation of a pro-tumorigenic microenvironment. Senescent cells contribute to these changes through senescence-associated secretory phenotype (SASP), which reinforces inflammation and matrix degradation, while extracellular vesicles (EVs) mediate intercellular signaling and further modulate ECM structure and function. In cancer, ECM remodeling not only facilitates tumor progression and metastasis by forming physical and biochemical barriers but also hinders the efficacy of conventional and immunotherapeutic interventions. Similarly, in cardiovascular diseases, aberrant ECM remodeling exacerbates tissue damage and impairs regenerative processes. Emerging therapeutic strategies aim to restore ECM homeostasis through targeted interventions, including ECM-normalizing agents, EV-based therapies, and stem cell approaches that modulate matrix composition to improve tissue repair. By elucidating the complex interplay between ECM dysfunction, cellular senescence, and chronic inflammation, this review highlights promising avenues for developing personalized treatments that address the underlying causes of age-related and tumorigenic pathologies, ultimately, the way to improved clinical outcomes.

## Introduction

The extracellular matrix (ECM) is a highly dynamic and complex network of proteins and other biomolecules that provides structural and biochemical support to cells and tissues. Beyond its fundamental role as a scaffold, the ECM actively regulates key cellular processes such as adhesion, migration, proliferation, differentiation, and apoptosis. It serves as a reservoir for growth factors and cytokines, mediates signal transduction through interactions with cell surface receptors (e.g., integrins), and contributes to tissue homeostasis, repair, and regeneration.

The mechanisms governing ECM secretion and assembly are highly conserved among eukaryotes, indicating their evolutionary origins long before the emergence of multicellular animals 1,2. Many ECM proteins exhibit oligomeric structures, with the most common motifs being the GXY repeats in collagens, which facilitate the formation of a stable triple helix, and the α-helical heptad repeats, which promote oligomerization into trimers, tetramers, or pentamers 2. These structural features are widely distributed across different biological domains, including viruses, suggesting an ancient and fundamental role for ECM components in cellular organization. Additionally, ECM proteins are characterized by the presence of repeated domains, such as the von Willebrand factor A (vWF-A) domain, the thrombospondin type 1 domain, and the epidermal growth factor (EGF) domain—all of which originated in early eukaryotic lineages and remain essential for ECM functionality 3,4. The evolutionary conservation of these domains underscores the critical role of the ECM in the development and maintenance of complex multicellular life.

The ECM is essential for maintaining tissue integrity and function across various physiological contexts. In development, ECM components guide cellular differentiation and organogenesis, while in wound healing, they coordinate tissue repair through tightly regulated deposition and remodeling 5-7. However, the dysregulation of ECM homeostasis contributes to a wide range of pathological conditions, particularly those associated with aging-related diseases, such as cancer, vascular calcification, fibrosis, and osteoarthritis 8-11.

As aging progresses, ECM components undergo biochemical and mechanical alterations, including increased collagen cross-linking, elastin degradation, and matrix stiffening 12. These changes compromise tissue elasticity and impair cellular function, ultimately contributing to conditions such as fibrosis, osteoarthritis, and vascular diseases 13. One prominent consequence of ECM dysfunction in aging is pathological calcification, characterized by the deposition of calcium phosphate crystals within the ECM 14. This process disrupts tissue architecture and promotes dysfunction, playing a central role in disorders such as vascular calcification, valvular heart disease, and skeletal pathologies. Inflammatory and metabolic factors further influence ECM calcification, exacerbating disease progression. Within aging-related conditions, ECM remodeling is a key driver of tumor progression 15. Aberrant ECM dynamics create a microenvironment that fosters cancer cell invasion, metastasis, and resistance to therapy. Increased ECM stiffness, excessive collagen deposition, and proteolytic ECM degradation facilitate epithelial-to-mesenchymal transition (EMT) and immune evasion, enabling malignant transformation and tumor expansion.

Given the critical role of ECM dysfunction in various diseases, extracellular vesicles (EVs) have emerged as promising therapeutic tools in regenerative medicine 16. EVs—including exosomes and microvesicles—act as natural carriers of bioactive molecules such as proteins, nucleic acids, and lipids, enabling the modulation of ECM composition and function 17. In degenerative diseases, EVs derived from mesenchymal stem cells (MSCs) have demonstrated the ability to restore ECM integrity by delivering reparative factors that stimulate fibroblasts, suppress inflammation, and enhance collagen synthesis 18. Furthermore, in fibrosis and cancer, engineered EVs can transport anti-fibrotic agents, matrix metalloproteinases (MMPs) inhibitors, or RNA-based therapies to regulate ECM remodeling and prevent disease progression.

A particularly promising application of EVs lies in targeted drug delivery 19. Functionalized EVs, designed with ECM-binding motifs, enable precise localization of therapeutic agents to affect tissues, thereby enhancing treatment efficacy while minimizing systemic toxicity. The emergence of EV-based therapies represents a paradigm shift in the management of ECM-related disorders, offering innovative strategies to restore tissue function and combat degenerative diseases 20.

This review provides a comprehensive analysis of the extracellular matrix (ECM), covering its biochemical composition, structural organization, and dynamic remodeling through synthesis, biogenesis, and enzymatic turnover. We explore its biological functions, including cell support, growth regulation, and its role as a signaling reservoir, as well as its involvement in Anoikis. In addition, we examine ECM interactions in pathological contexts such as cancer and aging, highlighting the role of extracellular vesicles (exosomes) in ECM modulation. We also review ongoing clinical trials and emerging ECM-targeted therapies, with a focus on regenerative medicine, oncology, and nanotechnology-based approaches to drug delivery and tissue engineering. This synthesis highlights the importance of ECM in health and disease and offers insights into its potential for therapeutic innovation.

## Components and structure of ECM

### Structure

As it is described above, ECM is important in regulating different cellular processes, such as adhesion, differentiation, proliferation, migration, and apoptosis 20. This regulation is facilitated by the intricate network of matrix components and their interactions with each other, signaling factors, and membrane receptors 21. ECM is an essential component of connective tissue, along with epithelial, muscle, and nerve tissue (the main tissue types in the human body) 22. The ECM is a complex mixture of water, proteins, and polysaccharides, and the balance of these components varies depending on the tissue type, such as cartilage, bone, fat, or tendon 23,24. As well as the tissue´s developmental stage and pathophysiological state. ECM components are synthesized and secreted locally by cells, primarily fibroblasts, which are the most numerous yet least specialized connective tissue cells 25. The organization of the ECM structure is influenced by the arrangement and orientation of the intracellular cytoskeleton 26.

Although the basic organization of the ECM is consistent, there are two primary types distinguished by their location and composition: the interstitial matrix and the pericellular matrix. The interstitial matrix forms a three-dimensional porous network that surrounds cells, particularly in connective tissue. In contrast, the pericellular matrix is more compact and forms a layer adjacent to the cells 27.

The interstitial matrix, often referred to as the proper matrix, forms the structural scaffolding for cells. Its basic components are heterotypic fibrils primarily composed of type I collagen, with smaller amounts of type III and V collagens in varying proportions, all of which play an essential role in fibrillogenesis in cells 28. These collagens are mostly secreted by fibroblasts. Additionally, important components of this amorphous three-dimensional gel include fibronectin and elastin, which are involved in the organization of the matrix structure 29.

A typical example of the pericellular matrix is the basement membrane, which is located at the interface between parenchyma and connective tissue. It provides a sheet-like anchoring layer that supports and stabilizes parenchymal cells, preventing them from tearing apart. Basement membranes are composed of collagen type IV, laminins, nidogens 1 and 2, and heparan sulfate proteoglycans (HSPGs) such as perlecan, agrin, collagen type XV, and collagen type XVIII 30-32. Also, it contains matricellular proteins that do not contribute to its physical stability or structural integrity but have regulatory roles. These proteins interact with surface receptors, proteases, hormones, and other biologically active molecules and may be specific to certain tissues in terms of function and structure. Examples of these proteins include SPARC (secreted protein acidic and rich in cysteine, also known as osteonectin, primarily associated with mineralizing tissues like bone), thrombospondin-1 (which is abundant in platelet α-granules and, when secreted, activates TGF-β1, or transforming growth factor-beta 1), and tenascin-C (which is expressed during embryonic development but is minimally detectable in adult tissues, becoming more apparent during pathological processes) 33-38. The basement membrane regulates tissue development, function, and regeneration by controlling cellular responses; it acts as a reservoir of growth factors, modulating their activity and concentration, and helps maintain the phenotype of surrounding cells 39. The interstitial matrix and basement membrane are closely interconnected, working together to ensure tissue integrity 40.

On the other hand, the cells embedded within the ECM interact with this macromolecular network through surface receptors, including integrins, discoidin domain receptors (DDRs), cell surface proteoglycans (PGs), and the hyaluronan (HA) receptor CD44 41. These interactions allow cells to integrate signals from the ECM that influence their functions and behavior. All cell types synthesize and secrete ECM macromolecules in response to multiple signals, contributing to ECM formation 42. Variations in ECM composition and structure affect both the architecture and biomechanical properties of the matrix, together with the signals transmitted to cells, thereby modulating their responses 43. Growth factors, cytokines, and chemokines are deposited with the ECM through binding to specific ECM molecules. These factors can be released in a controlled manner, influencing development and physiological processes at appropriate times 44. They are also actively involved in the reorganization of ECM. Besides synthesizing and secreting structural components, they also produce enzymes that degrade them. Remodeling processes are complex and must be tightly regulated to maintain environmental homeostasis 45.

Furthermore, ECM remodeling occurs under both physiological conditions and disease processes, influencing the structure and properties of ECMs in different ways 46. For instance, proteolytic degradation mediated by enzymes such as matrix metalloproteinases (MMPs), disintegrin and metalloproteinases (ADAMTS), plasminogen activators, and the degradation of HSPGs chains by heparinase, releases heparin-binding growth factors that activate angiogenesis and cell growth, particularly during tumorigenesis 47. During tumorigenesis, significant alterations in the ECM lead to the formation of fibrotic stroma characterized by increased stiffness, excessive deposition of ECM components, and the release of proteolytic enzymes that contribute to abnormal ECM remodeling 48. Enhanced lysyl oxidase (LOX) activity promotes the cross-linking of collagen fibers with other ECM components, further increasing matrix stiffness. Various cell types within the tumor stroma, such as cancer-associated fibroblasts (CAFs), endothelial cells, immune cells, pericytes, and even the tumor cells themselves, contribute to the development of deregulated and disorganized ECMs that support and promote tumorigenesis 49.

### ECM cycle

#### Extracellular matrix biogenesis: secretion and assembly

The biogenesis of the ECM is a fundamental, multi-step process essential for tissue development, maintenance, and repair. This process involves coordinated synthesis, secretion, and extracellular assembly and remodeling of a diverse set of macromolecules. Key structural ECM components include fibrous proteins such as collagens and elastins, proteoglycans, and adhesive glycoproteins like fibronectin and laminin 50. Their precursors are synthesized primarily by fibroblasts, chondrocytes, and osteoblasts, although epithelial and other mesenchymal cells may contribute to tissue-specific contexts 51.

Intracellular synthesis of collagen begins in the rough endoplasmic reticulum (RER), where the initial translation product, known as preprocollagen, contains signal peptides and globular domains at the N- and C-termini. Although Porter *et al* initially proposed a model of direct cytoplasmic extrusion of collagen fibrils at the cell surface, later autoradiographic and immunohistochemical studies, notably those by Revel and Hay, demonstrated that collagen follows the canonical secretory pathway via the RER and Golgi apparatus 52. These studies also revealed that newly synthesized collagen is typically deposited at some distance from the cell surface, suggesting that polymerization occurs extracellularly rather than directly on the plasmalemma 53. Within the cisternae of the RER, a series of PTMs is initiated to transform preprocollagen into a functional precursor suitable for further processing and secretion:

1. Cleavage of the signal peptide: The N-terminal signal peptide of the preprocollagen is cleaved upon entry into the lumen of the RER, generating procollagen.

2. Hydroxylation of proline and lysine residues: Specific proline and lysine residues in procollagen chains undergo post-translational hydroxylation catalyzed by prolyl hydroxylases and lysyl hydroxylases, respectively. These enzymes require ascorbic acid (vitamin C) as an essential cofactor. Hydroxylation is critical for the formation of intramolecular hydrogen bonds that stabilize the collagen triple helix. Deficiencies in this modification, as observed in scurvy, compromise collagen stability, leading to impaired wound healing, defective bone formation, and increased tissue fragility. In addition to its structural role, hydroxylation of lysine residues—particularly in type IV and VI collagens—is followed by glycosylation, a modification essential for the proper secretion, assembly, and spatial distribution of these collagen types. These functions are especially relevant in basement membranes and muscle tissues, where these collagen isoforms are most abundant 54.

3. Glycosylation of hydroxylysine residues: Some hydroxylysine residues are glycosylated by the addition of galactose or glucosyl-galactose moieties. Additionally, N-linked oligosaccharides are added to the terminal regions. This glycosylation modulates collagen secretion, assembly, and interactions.

4. Formation of the C-terminal globular domain: The carboxy-terminal propeptide region of each α-chain folds into a globular domain stabilized by disulfide bonds. This domain serves as a nucleation site for the proper alignment and registration of the three α-chains.

5. Assembly of the triple helix: Starting from the C-terminal end, three α-chains associate and fold into a right-handed triple helix. The N- and C-terminal propeptides remain non-helical.

6. Formation of specific inter- and intrachain hydrogen bonds and disulfide bonds that contribute to structural stability.

7. Association with molecular chaperones: The triple-helical procollagen interacts with molecular chaperones such as HSP47, which prevent premature aggregation and ensure proper folding and stabilization of the collagen trimer within the RER.

8. Lateral association and transport to the Golgi: Next, properly folded procollagen molecules begin to associate laterally via interactions at their non-helical terminal regions. These assembled precursors are packaged into secretory vesicles for transport to the Golgi apparatus, which delivers ECM components to the plasma membrane for extracellular release.

The formation of mature collagen fibrils, or fibrillogenesis, is a highly orchestrated extracellular process critical for the structural integrity of connective tissues. Following secretion, ECM components self-assemble into supramolecular structures, often initiated by fibronectin, which nucleates collagen fibrillogenesis and guides the organization of matrix architecture 55. The close association between the actin cytoskeleton and extracellular fibrils, observed in early electron microscopy (EM) studies and later confirmed by immunohistochemistry, highlights the dynamic interplay between cells and their surrounding matrix 56. This interface is mediated by integrins—transmembrane receptors that couple the ECM to the cytoskeleton and transduce biochemical signals via focal adhesion complexes and intracellular kinase cascades.

Following their secretion from the cell, procollagen molecules undergo enzymatic processing by membrane-associated procollagen N- and C-proteinases, which cleave non-helical propeptides at both ends 57,58. This proteolytic activation produces mature collagen monomers capable of assembling into higher-order structures. In particular, the serum concentration of the N-terminal propeptide of type I procollagen (PINP) serves as a clinically relevant biomarker for type I collagen synthesis and is often elevated in pathological states involving dysregulated extracellular matrix remodeling, such as bone metastasis in breast and prostate cancers 59.

Once activated, collagen molecules align in a row in the extracellular space and self-assemble in a longitudinal head-to-tail fashion 60. This precise alignment is facilitated by specialized invaginations of the plasma membrane called “fibrillogenesis bays,” which serve as nucleation sites for collagen assembly 61. These localized surface domains allow the accumulation and spatial coordination of secreted collagen molecules, promoting the formation of nascent fibrils with a defined orientation and periodicity.

The stability and mechanical properties of collagen fibrils are further enhanced by the enzymatic formation of covalent cross-links. These cross-links, mediated by lysyl oxidase, involve aldehyde groups generated from lysine and hydroxylysine residues and are essential for fibril maturation and resistance to tensile forces 62. The assembled fibrils aggregate laterally to form larger collagen fibers. These supramolecular structures confer remarkable tensile strength to tissues, with a strength-to-weight ratio comparable to that of steel, underscoring the indispensable role of collagen in maintaining the architecture and function of the extracellular matrix. In addition to enzymatic cross-linking, proper collagen assembly and function depend on post-translational modifications, including glycosylation of hydroxylysine residues catalyzed by lysyl hydroxylase 3 (LH3). Recent findings demonstrate that LH3 requires intracellular trafficking via interaction with VIPAR and VPS33B proteins, with the assistance of RAB10 and RAB25, for delivery to specialized collagen IV carriers in epithelial cells 63.

Recent evidence suggests that the assembly of type I collagen fibrils is initiated by a fibrillar nucleation complex enriched in type V and XI collagens 64. These molecules act as regulatory scaffolds that establish the initial fibril core, on which type I collagen molecules are subsequently deposited and polymerized. Type V and XI collagens play a critical role in controlling fibril diameter by limiting lateral growth once the desired thickness is reached.

In addition, mature collagen fibrils are often associated with the fibril-associated collagens with interrupted triple helices (FACIT) family, which includes collagens such as types IX and XII 65. These molecules are located on the surface of fibrils and mediate interactions with other components of the ECM, thus contributing to matrix organization and tissue-specific architecture. In cartilage, for example, type IX collagen is found on the surface of type II fibrils, anchoring them to proteoglycans and other ECM components and reinforcing the mechanical integrity of the tissue 66.

Collagen molecules are synthesized by various cell types in both connective and epithelial tissue. In connective tissue, collagen production is mainly carried out by fibroblast-like cells, including tissue-specific analogues such as chondrocytes in cartilage, osteoblasts in bone and pericytes in blood vessel walls 67-69. In addition, epithelial cells contribute to the synthesis of collagen components of basement membranes, underscoring the broad involvement of diverse cell types in collagen biogenesis 70.

The regulation of collagen synthesis is mediated by a complex network of signaling pathways involving growth factors, hormones and cytokines. For example, transforming growth factor beta (TGF-β) and platelet-derived growth factor (PDGF) are potent stimulators of collagen production by fibroblasts 70,71. In contrast, glucocorticoid hormones exert an inhibitory effect, down-regulating collagen gene expression and synthesis 72. These regulatory mechanisms are critical for maintaining tissue homeostasis and modulating extracellular matrix remodeling during development, repair, and disease.

In epithelial tissues, collagen fibrils are often arranged in highly ordered orthogonal lattices, such as those found in the dermis of aquatic vertebrates and in the corneal stroma of birds 73. To explain the development of these specialized architectures, Porter and colleagues proposed the “shingle” or scindulene hypothesis, according to which narrow layers of collagen are inserted obliquely into the basal lamina and are sequentially displaced by newly synthesized layers 74. This layered, anisotropic arrangement is thought to arise from tightly regulated interactions between secretory epithelial cells, the basal lamina, and the underlying ECM.

In connective tissues, the organization of collagen fibrils is tailored to the mechanical and functional demands of the tissue. In cartilage, type II collagen forms a dense three-dimensional network embedded in a hydrated matrix rich in proteoglycans, which confers tensile and compressive strength 75. Minor fibrillar collagens, such as types IX and XI, copolymerize with type II and modulate fibril diameter, interfibrillar spacing and cross-linking, thus contributing to the biomechanical integrity of the tissue.

In bone, type I collagen fibrils constitute the primary organic scaffold for mineral deposition 76. These fibrils exhibit a characteristic D-periodic banding pattern, reflecting the precise, staggered quarter alignment of tropocollagen molecules. This molecular organization is essential for the nucleation and orderly deposition of hydroxyapatite crystals, which endow the tissue with compressive strength. The hierarchical assembly of collagen in bone is orchestrated by osteoblasts and tightly regulated by local and systemic factors, such as mechanical stimuli, growth factors such as TGF-β and bone morphogenetic proteins (BMPs) 77. Taken together, these examples underscore the structural versatility of collagen fibrils, whose tissue-specific arrangement is governed by both intrinsic molecular properties and extrinsic cellular and biomechanical factors.

#### Extracellular remodeling

The ECM is continuously remodeled through a dynamic balance between synthesis and degradation, essential for tissue homeostasis, development, repair, and adaptation to mechanical and biochemical stimuli. Collagen fibrils, despite their structural stability, are not static entities; they are subject to slow but continuous turnover, with half-lives ranging from a few days to several years depending on tissue type, e.g., skin vs. cartilage 78. Degradation is initiated through mechanical stress, oxidative damage, or enzymatic cleavage and continues via proteolytic or phagocytic pathways 79,80.

##### Proteolytic degradation pathways

Proteolytic degradation occurs extracellularly, and the ECM can be cleaved by different families of proteases with overlapping substrate specificities (**Table 1**).

*Matrix metalloproteinases*. Matrix metalloproteinases (MMPs), a family of zinc-dependent endopeptidases secreted by fibroblasts, chondrocytes, macrophages, neutrophils, epithelial cells (e.g., keratinocytes), and cancer cells 81. MMPs act on various components of the ECM: collagenases (e.g. MMP-1) degrade native fibrillar collagens (types I, II, III and X), gelatinases (MMP-2 and MMP-9) cleave denatured collagens (gelatins), laminin, fibronectin and elastin, stromelysins (e.g. MMP-3) degrade proteoglycans, fibronectin and non-helical collagens, matrilysins target basement membrane components such as type IV collagen and proteoglycans, membrane-type MMPs (MT-MMPs), produced mainly by tumor cells, exhibit potent pericellular proteolytic activity and macrophage metalloelastases (e.g. MMP-12) degrade elastin, type IV collagen and laminin 82-86. Their expression and activity are typically low under physiological conditions but are markedly upregulated during tissue remodeling, inflammation, and pathological states such as cancer and fibrosis. MMPs are synthesized as inactive zymogens and are subsequently activated extracellularly through proteolytic cleavage by serine proteases or other MMPs, or via oxidative modification of a regulatory cysteine residue 87,88.

*Adamalysins*. This family includes disintegrinases and metalloproteinases (ADAMs) and ADAMs with thrombospondin motifs (ADAMTS). Of the 22 human ADAMs, 12 are catalytically active. ADAMs, often membrane-anchored, function as sheddases, cleaving ectodomains of membrane-bound proteins such as cytokines, growth factors, and receptors. Some ADAMs (e.g., ADAM10, ADAM12, ADAM15) can also degrade ECM proteins 89. ADAMTS enzymes are secreted proteinases characterized by type I thrombospondin motifs. Several members of the ADAMTS family (e.g., ADAMTS4 and ADAMTS5) are aggrecanases, involved in proteoglycan degradation. Others (ADAMTS2, -3, -14) function as procollagen N-propeptidases, essential for the maturation and deposition of collagen fibrils 90. ADAMTS13 cleaves von Willebrand factor and is essential for hemostasis 91.

*Meprins*. Meprins are zinc-dependent metalloproteinases belonging to the astacin family 92. They are composed of α and β subunits that form homo- or hetero-oligomeric complexes. Meprin-α is secreted, whereas meprin-β is membrane-bound but can be shed via ADAM10 93. Meprins cleave several ECM components, including collagen IV, nidogen, and fibronectin. They also contribute to the processing of procollagen I and can regulate other metalloproteinases, such as promoting MMP-9 activation via MMP-3 94.

*Other enzymes are involved in ECM remodeling*. On the one hand, serine proteases such as plasmin (activated by urokinase and tissue plasminogen activators) degrade fibrin, fibronectin, and laminin 95. Neutrophil-derived elastase targets elastin and fibronectin, while matriptase, a serine protease bound to the epithelial membrane, maintains the integrity of the epithelial barrier 96,97. On the other hand, cathepsins are lysosomal proteases with serine (cathepsins A and G), aspartic (cathepsins D and E), and cysteine (e.g., cathepsins B, K, L) variants 98,99. They can function extracellularly or degrade ECM components after endocytic uptake and lysosomal processing. Finally, heparanase cleaves heparan sulfate proteoglycans (HSPG), altering ECM structure and releasing bound growth factors and cytokines 100. Sulfatases 1 and 2 remove 6-O-sulfate groups from HSPGs, modulating the binding and signaling of factors such as FGF1 and VEGF 101.

##### Regulation of the proteolytic pathway

Endogenous inhibitors are essential for maintaining the integrity of the extracellular matrix (ECM) by tightly controlling proteolytic activity (**Table 2**). Tissue inhibitors of metalloproteinases (TIMPs)-TIMP1 to TIMP4- regulate the function of matrix metalloproteinases (MMPs), as well as members of the ADAM and ADAMTS families, with distinct binding specificities 102. Among them, TIMP3 is sequestered in the ECM, whereas the others exist predominantly in soluble form. TIMP3 is also the main inhibitor of ADAM and ADAMTS 103. Other regulatory molecules include RECK (reversion-inducing cysteine-rich protein with Kazal motifs), which modulates both MMP and ADAM activity, and other endogenous inhibitors such as cystatin C, elafin, and fetuin A, which have been shown to suppress meprin activity 104. ECM turnover is governed by complex regulatory networks involving genetic, biochemical, and mechanistic signals. The balance between proteolytic enzymes and their inhibitors, exemplified by the MMP/TIMP ratio, determines the net proteolytic capacity within a given tissue and is finely tuned by transcriptional control, zymogen activation, and inhibitor availability 105. Intact triple helix collagens are relatively resistant to proteolysis; however, once structurally compromised by mechanical stress or oxidative injury, they become more vulnerable, especially to gelatinases such as MMP2 and MMP9 106. Disruption of the delicate balance between MMPs and their inhibitors contributes to pathological degradation of the ECM, as seen in chronic inflammatory conditions, tumor invasion, and degenerative diseases such as rheumatoid arthritis and osteoporosis.

##### Phagocytic degradation pathway

Phagocytic degradation constitutes a crucial intracellular mechanism for the turnover of ECM components, particularly fibrillar collagens. In this pathway, relatively intact collagen fibers are recognized and internalized by fibroblasts or macrophages via receptor-mediated endocytosis, often facilitated by β1-integrins (e.g., α1β1, α10β1, α11β1) that bind to noncollagenous components such as fibronectin or proteoglycans decorating the collagen surface 107. The formation of actin-rich pseudopodia allows engulfment of collagen segments in phagocytic vesicles 108. Membrane matrix metalloproteinase type 1 (MT1-MMP), located on the cell surface, initiates partial cleavage of collagen prior to internalization 109. Once inside the cell, the phagocytosed collagen is transported to the lysosomes, where it is degraded by lysosomal cysteine proteases, such as cathepsins. This pathway complements extracellular degradation by allowing the removal of ECM debris and promotes tissue remodeling under physiological conditions. In contrast to the rapid extracellular cleavage that predominates in pathological contexts, phagocytic degradation proceeds at a slower rate and is particularly important for the homeostatic maintenance of ECM integrity and the resolution of matrix turnover during tissue repair 110.

**Figure 1:**
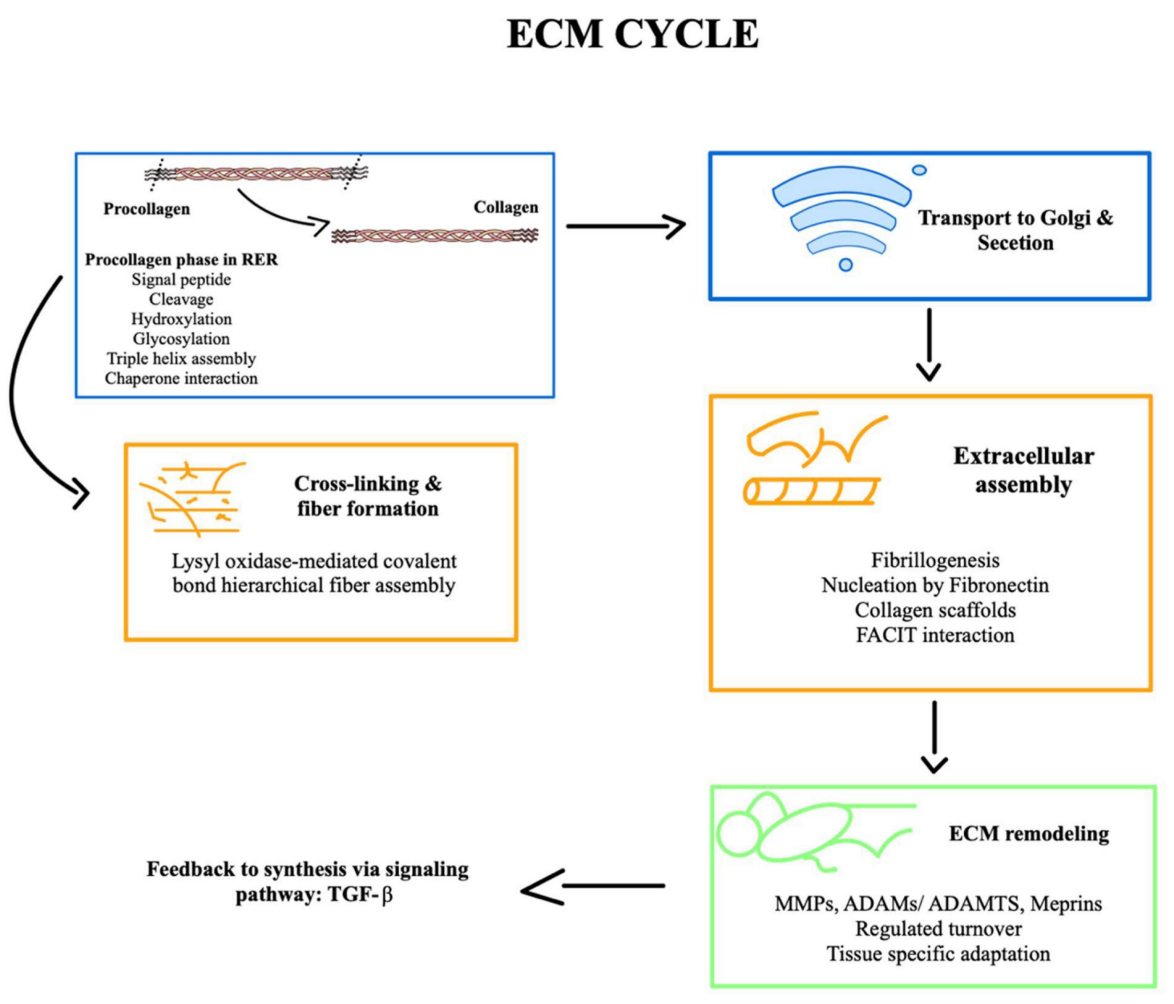
A schematic graphic of the development of the ECM cycle is presented to illustrate the dynamic processes involved in extracellular matrix remodelling and to provide a clear visual overview of its key stage and interactions.

To summarize this explanation of the ECM cycle, a schematic graphic is provided to emphasize its key stages (**Figure 1**).

### Components

#### Collagen and elastic fibers

**Collagen** terms refer to a family of glycoproteins distinguished by three key features. The first is the repeating amino acid sequence [Gly-X-Y]_ n_, which can include interruptions. The second feature is the common presence of proline and its hydroxylated form, hydroxyproline, in the X and Y positions, respectively. The third defining characteristic is the unique quaternary structure: a right-handed triple helix formed from three left-handed polyproline α-chains of identical length 111, which adopts a polyproline type II-type helical conformation and coils around each other. Interchain hydrogen bonds hold the α-chains together. The small hydrogen atom side chain of glycine in every third residue within the α-chains allows them to pack tightly together in a triple helix, with this residue in the interior of the helix and the rings of proline and 4-hydroxyproline pointing outward 112. Collagens also have non-collagenous (NC) non-triple helical domains at both C- and N-termini, which are numbered from the C-terminus (NC1, NC2, NC3, etc.) 113.

It makes up to 30% of the total protein in humans. It is synthesized and secreted into ECMs primarily by fibroblasts. It is the most abundant fibrous protein within the interstitial ECMs of all animals and is also found in pericellular matrices such as the basement membrane 114. The discovery of transmembrane collagens on the surface of various cell types containing bioactive peptides liberated upon degradation has amplified interest in collagen biology. By exerting tension on the matrix, fibroblasts organize collagen fibrils into sheets and cables, significantly influencing the alignment of collagen fibers 115.

It forms part of a superfamily of twenty-eight types, each formed by at least forty-six unique polypeptide chains (α chains) in vertebrates. Collagen type I, the most prevalent collagen, is widely expressed across various tissues. It forms ideal heterotrimeric triple helices that naturally assemble into fibrils and are a key structural component in tissues such as skin, bone, and tendons 116. Collagen types vary significantly in their structure and functions. Some have breaks in their triple helices and do not self-assemble, while others, like transmembrane collagens, exhibit long interruptions and are crucial for cell signaling and adhesion 117.

Numerous mutations have been identified in collagen genes that can affect trimerization, collagen network formation, and propeptide cleavage. These mutations are associated with various clinical pathologies, such as Ehlers-Danlos syndrome (collagen types I, III, V) 118, osteogenesis imperfecta and osteoporosis (collagen type I) 119, osteoarthrosis (collagen type II, IX, X, XI) 120, chondrodysplasias (collagen types II, IX, X, XI) 121,122, arterial aneurysms (collagen type III) 123, Bethlem myopathy and Ullrich muscular dystrophy (collagen type VI) 124, epidermolysis bullosa acquisita (collagen type VII) 125, generalized atrophic epidermolysis bullosa (collagen type XII) 126, Fuchs endothelial corneal dystrophy (collagen type VIII) 127, Knobloch syndrome (collagen type XVIII) 128, Alport syndrome (collagen type IV) 129, and Schmid metaphyseal chondrodysplasia (collagen type X) 130.

Collagens are categorized into seven groups based on their domain homology and functions. These categories include:

- **Fibrillar collagens**: Including types I, II, III, V, XI, XXIV, and XXVII, are important for providing tensile strength to tissues. These collagens form 67-nm periodic fibrils through a regular staggered arrangement of triple helical molecules, with fibril diameters ranging from 12 nm to over 500nm, depending on development 131. Their formation is influenced by other matrix macromolecules such as decorin and biglycan. Fibrillar collagens feature a long, uninterrupted Gly-X-Y domain and are flanked by N- and C-terminal propeptides. The α chains form homo- and heterotrimeric helices, with NC1 domains ensuring correct alignment for triple helix nucleation 132. Post-translational modifications in the endoplasmic reticulum include hydroxylation, glycosylation, and disulfide bridge formation. Procollagen triple helices are cleaved by specific proteinases from tropocollagen, which then assemble into collagen microfibrils. LOX facilitates the formation of cross-links, stabilizing fibrils and contributing to their mechanical properties 133. However, collagen types XXIV and XXVII exhibit imperfections in their Gly-X-Y sequences, indicating very short interruptions in their triple helical structure. Collagen fibrils found in the dermis, tendons, and other tissues are often composites of various collagen types, typically types I, III, and V 134. These composite fibers are known as heterotypic fibrils, in contrast to homotypic fibrils, which consist of a single collagen type, such as collagen VII found in fibrils are the dermo-epidermal junction 134. While collagen type I often predominated in a tissue, various types, and matrix macromolecules interact to impart specific structural and functional characteristics. Beyond their mechanical support role, collagen scaffolds also influence cell migration, adhesion, angiogenesis, tissue development, and repair 135.
- **Network-forming collagens:** Include types IV, VIII, and X, each playing distinct roles in various tissues. Collagen type IV, a key component of basement membranes, is essential for molecular filtration 136. Collagen type VII is found in Descemet´s membrane and vascular sub-endothelial matrices 137, while collagen type X is present in the hypertrophic zone of growth plate cartilage 138. These collagens feature interruptions in their triple helices, allowing flexibility and extensive network formation. Collagen type IV begins folding at the C-terminus and progresses towards the N-terminus. The NC1 domain facilitates the tail-to-tail association of trimeric molecules, forming hexamers stabilized by Met-Lys cross-links 139. This assembly leads to the creation of a two-dimensional network. Similarly, collagen types VIII and X form polygonal lattices, with the NC1 domains for their supramolecular assembly. Additionally, these collagens interact with various ECM components to form complex multimolecular networks 140. They offer structural support and attachment for cells and tissues, and function as a filtration barrier for macromolecules in organs like the kidneys 141.
- **FACITs:** encompass collagen types IX, XII, XIV, XVI, XIX, XX, XXI, and XXII. These collagens are relatively short and feature NC domains that interrupt their triple helical collagenous domains, granting them flexibility. FACITs interact with fibrillar collagens on their surfaces, connecting collagen fibers and other ECM molecules 142. For instance, collagen type IX is covalently bound to the surface of collagen type II fibrils, with collagenous domains 1 and 2 positioned between NC1 and NC3 domains. The NC4 domain extends into the NC3 hinge region. Collagen type IX is essential for maintaining cartilage integrity. Collagen type XII associates with collagen type I and II fibrils, while collagen type XIV co-localizes with collagen type I indirectly through binding to DS chains of decorin, which in turn associates with collagen type I fibrils. In skin, collagen type XVI is incorporated into microfibrils, which are molecular composites, primarily consisting of collagen II and containing collagen type XI as a minor component 143.
- **MACITs** are type II transmembrane proteins characterized by a long extracellular C-terminal domain with collagenous segments interrupted by NC domains and a short cytoplasmic N-terminal domain. This group includes collagen types XIII, XVII, XXIII, and XXV, which are produced by various cells, including malignant ones, and different tissues. These proteins function as cell surface receptors, influencing cell adhesion. When proteolytically cleaved, they release from the cell surface into the extracellular matrix, creating soluble collagens. For instance, the ectodomain of collagen type XVII can be cleaved by ADAMs, thereby modulating cell motility 144.
- **Anchoring fibrils:** Collagen type VII is the primary component of anchoring fibrils located beneath the lamina densa of the basement membrane, linking it to the underlying stroma. This collagen type is created by the homotrimerization of α1 (VII), featuring a central triple helical collagenous domain interrupted by a short NC domain and flanked by N- and C-terminal NC domains. Two collagen type VII molecules dimerize and subsequently assemble into anchoring fibrils 145.
- **Beaded-filament-forming collagens:** Include types VI, XXVI, and XXVIII. Among these, collagen type VI is the most extensively studied. It is widely expressed in tissues, where it interacts with various ECM proteins, HA, PGs, and collagen type IV in basement membranes. Collagen-type VI molecules form antiparallel dimers through staggered alignments of monomers. These dimers are then associated laterally to create tetramers, which are stabilized by disulfide bonds. The tetramers connect at their globular ends, forming beaded filaments characterized by 25 nm beads spaced 100 nm apart 146.
- **Multiplexin:** Collagen type XV and XVIII fall into the category of Multiplexins/endostatin-producing collagens. These types are widely expressed across all vascular and epithelial basement membranes in human tissues. Collagen type XV connects adjacent collagen fibrils, forming various oligomeric assemblies that enhance the adhesion of basement membranes to the underlying connective tissue stroma 147. Both collagen types XV and XVIII feature a central triple helical collagenous domain interrupted by multiple NC domains, with collagen XV carrying CS chains and collagen XVIII carrying HS chains. Collagen XVIII is a homotrimer made up of three α1 chains, containing ten interrupted collagenous domains flanked by eleven NC domains at their N- and C-termini 148. It can carry three HS chains. Bot collagen types have a C-terminal NC domain that includes anti-angiogenic endostatin and endostatin-like modules. Cleavage of part of the NC1 domain releases endostatin, which interacts with several receptors such as integrins α5β1, αvβ5, and VEGFR2. These interactions disrupt the actin cytoskeleton, trigger a signaling network that downregulates the VEGF signaling pathway, and stimulate potent angiostatin components like TSPs, thereby significantly inhibiting angiogenesis 149.

All these collagen groups are summarized in the following **Table 3**.

**Elastic** fibers are structural elements of the extracellular matrix, providing resilience to connective tissues. They are abundant in tissues that need mechanical flexibility, such as arteries, skin, lungs, and cartilage 150. Elastic fibers consist of two primary components: elastin, a core-forming amorphous protein, and surrounding microfibrils composed of glycoproteins 151. It is primarily produced from the *ELN* gene located on chromosome 7. The *ELN* gene is active during prenatal development and early life; it significantly decreases during adulthood. Mutations in the *ELN* gene can result in improper tropoelastin production or assembly, leading to diseases like supravalvular aortic stenosis (SVAS) and Williams-Beuren syndrome (WBS), where elastic fiber deficiency contributes to arterial stenosis 152,153.

The key precursor to elastin is tropoelastin, a protein with alternating hydrophobic and cross-link domains. Tropoelastin is secreted as a soluble polypeptide, with a molecular weight of around 70-72 kDa, into the extracellular matrix, where it self-assembles into globular aggregates through a process called coacervation, driven by its hydrophobic regions 154. The polymerization of tropoelastin begins with the oxidative deamination of lysine reduced, catalyzed by LOX. These cross-links give rise to the durable elastic fibers that provide issues with their resilience 155. The process also involved the formation of two amino acids, desmosine and isodesmosine, which further stabilize the elastic fibers through inter- and intra-molecular bonds. Tropoelastin deposition on microfibrils comprises glycoproteins such as fibrillin-1 and fibrillin-2, which are necessary to assemble mature fibers properly 156.

Beyond fibrillins, MAGP-1 (microfibril-associated glycoprotein-1) emerges as a significant structural component for microfibrils. Widely expressed in mesenchymal and connective tissues, MAGP-1 is associated with nearly all microfibrils 157. It binds to the amino-terminal exons of fibrillin-1 through its carboxyl-terminal region, which contains common cysteine residues. This interaction is key to forming stable complexes between fibrillin-1 and other proteins, including decorin, biglycan, and tropoelastin, which all contribute to the structural assembly of elastic fibers 158.

Microfibrils guide the spatial arrangement of tropoelastin and promote the orderly cross-linking needed for fiber maturation. The final step involves extensive cross-linking to form a stable, hydrophobic, and insoluble polymer that resists proteolysis 159.

Elastic fibers, once formed, are stable. Elastin is so durable that it rarely breaks down or gets replaced throughout a person´s life 160. This is one reason why elastin is so important for maintaining the structure and function of tissues, especially in organs that need to expand and contract, such as the lungs and heart 161,162. Over time, however, changes in elastin, like reduced cross-linking or damage from environmental factors, can cause tissues to lose their elasticity.

This leads to common signs of aging, such as sagging skin or stiffening arteries. Elastin´s long-lasting maturity makes it a key focus in understanding how tissues age and how certain diseases related to elastic fiber defects, such as aortic stenosis and skin disorders, develop 163.

#### Multi-adhesive glycoproteins

##### Fibronectin (FN)

It is a glycoprotein that ranges in size from 230 to 270 kDa and exists as a dimer, with two subunits covalently bonded at their C-terminal by a pair of disulfide bonds 164. Each monomer comprises three repeating units: 12 Type I, 2 Type II, and 15-17 Type III domains, which together constitute 90% of the FN sequence.

Similar structural domains (Type I, II, and III) are found in other biomolecules, suggesting FN evolved through exon shuffling 165. While the conformations of type I and type II repeats are stabilized by pairs of intramodule disulfide bridges, the type III repeat forms a 7-stranded β-barrel structure that lacks disulfide bonds, allowing it to undergo conformational changes. Despite being long encoded by a single gene, FN exists in multiple variants due to extensive alternative splicing 166. The domains or units of FN facilitate self-assembly and binding to ligands such as collagen/gelatin, integrins, heparin, fibronectin, and other extracellular molecules 167. The 500 kDa FN dimer is created through a pair of antiparallel isoforms due to alternative splicing. FN exists in multiple isoforms due to alternative splicing. A single FN gene transcript encodes 20 isoforms in humans 168. It is secreted as soluble inactive dimers with disulfide bonds, which must be activated by interaction with α5β1 and other integrins 169.

FN can be classified by its solubility into soluble plasma FN and cellular FN. Cellular FN is significantly more diverse because of splicing variations that are specific to different cell types and species 170.

According to its expression, it has an important role in embryos and adults, especially in areas of morphogenesis, cell migration, and inflammation. It is low in tumor cells, but it has a high expression in tissues undergoing repair 171. FN facilitates the formation of fibrillar networks by binding to cell receptors like 5β1 integrin, organizing the actin cytoskeleton, and exposing additional binding sites for fibril formation. This process is essential for matrix assembly and the organization of other ECM proteins. FN´s ability to simultaneously bind to various ECM components makes it an organizer, particularly important for assembling a fibrillin-1 network 171.

##### Fibrinogen

It is an intricate fibrous glycoprotein composed of three pairs of polypeptide chains: Aα, Bβ, and γ. These chains are interconnected by 29 disulfide bonds. The fibrinogen molecules measure 45 nm in length and feature globular domains at both ends and, in the center, linked by α-helical coiled-coil rods, giving it a molecular weight of 340 kDa 172. The E-region comprises the N-terminal ends of all six chains, while the D-regions consist of the C-terminal ends of the Bβ and γ chains along with part of the Aα chain, all connected by three-stranded α-helical coiled-coil regions. Both strongly and weakly bound calcium ions play roles in maintaining the structure and function of fibrinogen 173. Its fibrinopeptides are cleaved by thrombin to convert soluble fibrinogen into soluble fibrin. This involves interactions between exposed knobs and holes, leading to the polymerization of fibrin monomers into protofibrils 174. These protofibrils laterally aggregate to form fibers that branch into a three-dimensional fibrin clot essential for hemostasis 175. Factor XIIIa further stabilizes the clot by linking fibrin molecules via isopeptide bonds 176.

The fibrinolytic system uses plasminogen, activated to plasmin, to degrade fibrin at specific lysine residues. Fibrinogen binds various proteins, influencing cardiovascular and ECM functions but not directly affecting important disorders discussed 177. Research on dysfibrinogenemia and variant fibrinogen molecules has deepened the understanding of its functions 178. Fibrinogen interacts with activated αIIbβ3 integrin on platelets, facilitating platelet aggregation for hemostasis, and it has adhesive and inflammatory roles through interactions with other cells 179.

##### Fibulins

Are a family of seven glycoproteins secreted by various cell types and tissues. They are intricately linked with basement membranes, elastic, fibers, and other extracellular ECM 180. These proteins feature a fibulin module that allows them to release glycoproteins and possess a globular domain at their carboxy terminus. This domain is preceded by multiple tandem repeats of calcium-binding epithelial growth factor (cb-EGF) sequences. Research by Argraves has shown that fibulins are complex proteins with two distinct repeating motifs. One of these motifs shares similarities with anaphylatoxins C3a, C4a, and C5, as well as with the albumin gene family, while the other is like EGF 181.

The fibulin family is divided into classes based on length and domain configurations: Class I and Class II 182. Class I is formed by fibulins 1, 2, and 6. Class II encompasses the shorter (50-60 kDa) fibulins 3, 4, 5, and 7. These shorter fibulins play a role in the formation of elastic fibers and are active during embryonic development in skeletal and cardiovascular tissues, with calcium ions aiding in this process 183.

Fibulin-2, part of the fibulin family, is recognized for its ability to engage with various ECM ligands and modulate the interaction between cells and their environment 184. On the other hand, fibulin 3 is primarily found in mesenchyme that transforms into cartilage to bone, while fibulin 4 is notably present in heart muscle. Fibulin 5 is expressed in blood vessels, and fibulin 7 is abundant in teeth, placenta, hair follicles, and cartilage 185-188. All fibulins have a C-terminal module with an elastic-binding domain, which is prominent in fibulin 5 189.

Fibulins act not only as structural elements of the extracellular matrix but also as regulators of various cellular activities, including growth, differentiation, angiogenesis, and tumor development. They play a key role in modulating cellular behavior and function 190.

##### Fibrillins

They are a group of substantial extracellular glycoproteins (350 kDa) comprising three isoforms: Fibrillin-1, fibrillin-2, and fibrillin-3. These molecules feature 40-80 amino acid residues and multiple cbEGF domains interspersed with several motifs containing eight cysteines (TB motifs) that bind TGFβ. No other extracellular proteins contain as much cysteine as fibrillins 191. While fibrillin-2 and fibrillin-3 are found in embryonic tissues, with some presence in peripheral nerves, skin, and tendons, fibrillin-1 is present in both embryonic and adult tissues 192.

They are the principal component of microfibrils in both elastic and non-elastic extracellular matrices, interacting closely with tropoelastin and integrins through direct binding. Microfibrils maintain structural integrity in specific organs and serve as scaffolds for elastogenesis in tissues like skin, lungs, and blood vessels 193. Thus, fibrillin is used to incorporate elastin into elastic fibers. Various mutations, including those in the propeptide sequence encoded by the C-terminal domain of the fibrillin-1 gene, result in defective microfibril assembly in individuals with Marfan syndrome 194. Besides fibrillins and elastin, numerous other proteins contribute to the makeup of microfibrils 195. Fibronectin has a role in the process of binding a C-terminal fibrillin-1 region with the fibronectin gelatin-binding region. Homocysteinylation of fibronectin homocysteinylation reduces fibronectin dimers to monomers, impairing the assembly of fibrillin and microfibrils, like the effects of homocysteinylation of fibrillin-1 196. Fibrillins contain several TGF-binding motifs, making their structure and function akin to latent-TGF-binding proteins 197.

##### Laminins

Represent a fundamental group of large glycoproteins that function with the extracellular matrix, particularly in the basement membranes of tissue 198. Their molecular weights typically range from 400 to 900 kDa, depending on the isoforms and subunit composition 199. These molecules are composed of three distinct subunits: the first one, alpha (α), ranges between 160 and 400 kDa, the second one (β) from 120 to 210 kDa, and the third one, gamma (γ), from 150 to 200 kDa, which combine to form heterodimers. Out of the 11 identified in mammalian subunits, 16 different Laminin isoforms have been characterized 200. Each isoform is designated by a code based on its subunit composition, enhancing its structural components and its function in various tissues, like Lm111 (α1β1γ1) or Lm211 (α2β1γ1). These subunits assemble into heterotrimers via a long coiled-coil region, forming laminins essential to the basement membrane's structure and function. The full hetero trimeric laminins vary in size but often measure around 800-900 kDa 201.

Laminin exhibits differences in its polymerization capabilities and interactions with cellular receptors, contributing to basement membranes' specific makeup across diverse tissues 202. Most basement membranes contain laminin isoforms, suggesting that their combination contributes to the dynamic properties 203. The structure of laminins varies, with some forms taking on a cross-like configuration. These forms possess three short arms, each capped by LN domains, and a longer arm, facilitating the interaction with nidogen and promoting polymerization 204. Laminin with truncated subunits, such as those containing the α3A, α4, or γ4 chains, lacks the complete short arms and corresponding LN domains, thus precluding polymerization. These perform other functions related to signaling and structural organization without contributing to the polymer scaffold 205. The long arms of laminin molecules extend from the subunit, terminating in a set of globular domains (LG). These domains interact with integrins, dystroglycan receptors, and other cell surface molecules such as sulfated glycolipids and heparan sulfate 206. This interaction with cellular components enables laminins to regulate a range of biological processes, including cell adhesion, differentiation, and migration, which are essential for maintaining tissue integrity and function 207.

Laminin mutations, particularly in Lm332, can lead to severe diseases such as Herlitz-type junctional epidermolysis bullosa (JEB), underscoring the importance of these glycoproteins in tissue stability 208. In contrast, the α3B splice variant is present in various developing tissues, including the brain, and it contributes to polymerizing laminins with stronger self-association capabilities, which may enhance their role in tissue development and repair 209.

##### Osteopontin

It is a bone matrix glycoprotein presented in bone and dental tissues by mediating the interactions between cells and minerals 210. Osteopontin (OPN) is classified as a matricellular cytokine and functions in processes such as bone remodeling and bone formation under mechanical stress 211. Structurally, OPN is an intrinsically disordered protein (IDP) enriched with acidic residues, particularly aspartic and glutamic acids, which constitute about 25% of its sequence 212. The molecular weight of OPN typically ranges between 44 and 75 kDa, depending on its level of phosphorylation, which significantly influences its functionality 213. Phosphorylation of OPN is a post-translational modification, regulating its interaction with calcium phosphate minerals. As a member of the SIBLING family (small integrin-binding ligand N-linked glycoproteins), OPN contains an ASARM motif known for inhibiting extracellular matrix mineralization by binding hydroxyapatite crystals. This inhibitory activity is controlled by the protease PHEX, which cleaves OPN into inactive fragments, thus modulating its regulatory role in mineralization 214.

ECM mineralization, such as hypophosphatemia, hyperphosphatemia, and hypophosphatasia 215-217. In these models, full-length OPN was detected in bone extracts of Hyp and Fgf23 -/- mice, and its phosphorylation level was shown to decline. These conditions lead to an aging-like skeletal phenotype characterized by impaired mineralization and osteomalacia, where the ECM becomes compromised 218.

##### Nidogen

It is a sulfated glycoprotein consisting of three globular domains (G1, G2, and G3), a short linker, a rod-like domain, and is conserved across species 219. These domains mediate carious interactions within the ECM, particularly with Collagen IV and Laminin, establishing a structural framework for tissue integrity 220. Nidogen´s G2 domain binds to Collagen IV, while the G3 domain interacts with Laminin, facilitating the formation of stable ternary complexes 221. These molecular interactions serve as a key element in maintaining the ECM´s architecture, especially at the basement membrane, contributing to cellular adhesion and tissue stability 222. In addition to structural functions, Nidogen plays an important role in neuronal development, particularly in directing axon migration and forming neuromuscular junctions 223. In Nidogen mutations, disruptions in nerve positioning and muscle connectivity result in motor and behavioral defects, further demonstrating its importance for proper ECM function in neural tissues 224. Mice with Nidogen-1 mutations exhibit neurological abnormalities, including seizure-like symptoms and impaired muscle control 225. Mutations in Nidogen have been linked to developmental disorders, such as Dandy-Walker malformation, where abnormalities in ECM integrity lead to defects in epithelial morphogenesis and neural development. Studies suggest that disruptions in the Nidogen-Laminin interaction contribute to a wide range of phenotypic variability, from subtle skeletal defects to severe neurological conditions 226,227.

#### Proteoglycans

Proteoglycans are complex molecules composed of glycosaminoglycan (GAG) chains covalently attached to a core protein, primarily located on the cell surface or within the extracellular matrix 228. They maintain tissue hydration and act as molecular sieves in basement membranes. Their GAG chains, which contain repeating disaccharides of uronic acid and acetylated or sulfated hexosamines, vary in length based on available Ser-Gly sites on the protein core 229. Proteoglycans include several types, such as heparan sulfate, chondroitin sulfate, and keratan sulfate, each with distinct disaccharide compositions. Classified into four main types, proteoglycans exhibit specific cellular distributions 230.

Serglycin is the only proteoglycan that forms part of the intracellular group, storing proteases with mast cell granules 231. In contrast, heparan sulfate proteoglycans (HSPGs) are primarily associated with cell surfaces, where they support growth factor functions and interactions within the basement membrane 232. HSPGs facilitate cellular communication and sustain morphogen gradients essential for development and regeneration by binding to growth factors like FGF and VEGF and structural ECM components 233. Also, proteoglycans containing chondroitin and dermatan sulfate (CSPGs and DSPGs) become predominant 234. These proteoglycans have an important role in the structure of complex matrices, including cartilage, brain, intervertebral discs, tendons, and corneas. Their functions include providing viscoelasticity, retaining water, maintaining osmotic pressure, ensuring organized collagen arrangement, and preserving corneal transparency 235. Additionally, the ECM hosts the largest class of proteoglycans, small leucine-rich proteoglycans (SLRPs), which are abundant at the gene level. SLRPs act as structural elements and signaling molecules, especially during tissue remodeling associated with cancer, diabetes, inflammation, and atherosclerosis 236. By interacting with receptor tyrosine kinases (RTKs) and toll-like receptors, SLRPs influence migration, proliferation, immune responses, apoptosis, autophagy, and angiogenesis 237.

##### Syndecan

It is a family that comprises transmembrane proteoglycans that connect cells to the ECM. Syndecans feature an ectodomain that binds GAG chains and a cytoplasmic domain with a PDZ-binding motif that anchors them to the cytoskeleton 238. Syndecans are involved in a wide range of cellular functions, including growth factor binding, formation of morphogen gradients, endocytosis, and lipoprotein clearance, particularly through Syndecan-1 239. Proteolytic shedding of Syndecans, induced by cytokines and enzymes, regulates their presence on the cell surface and within the pericellular environment 240. Shed syndecan-1, particularly in cancer, promotes tumor growth and metastasis, while syndecan-2 can inhibit angiogenesis by reducing endothelial cell migration 241,242. A novel function of syndecan-1 includes nuclear translocation in cancer cells, where it affects gene transcription by modulating enzymes such as histone acetyltransferase (HAT), promoting tumorigenic gene expression 243.

##### Glypicans (GPCs)

HSPGs are attached to the plasma membrane by a glycosylphosphatidylinositol (GPI) anchor 244. Six genes encode glypicans, divided into two groups with orthologs found across species. Glypicans have unique features, including GAG attachment sites close to the membrane. Allowing their HS chains to bind morphogens like Hedgehog (Hh), Wnt, and FGF and modulate cell signaling 245,246. Glypicans undergo two types of processing; the first one is furin-like proteases that cleave to the ectodomain, creating two disulfide-linked subunits, and the second one, extracellular lipases, like Notum, release glypicans from the membrane, regulating morphogen gradients such as Wnt and BMP 247,248. Functionally, glypicans regulate growth and angiogenesis and have implications for cancer. For instance, GPC3 mutations cause Simpson-Golabi-Behmel syndrome, which involves overgrowth and developmental abnormalities. While initially thought to inhibit IGF-II, GPC3 was later shown to suppress Hh signaling, binding Indian and Sonic Hh proteins and competing with the Patched receptor 249,250. This suppression depends on HS chains and their sulfation. Glypicans ´complex regulation by proteases and lipases suggests evolving insights into their roles in biological processes 251.

##### Betaglycan

Also called the TGFβ type III receptor (TGFβ3), it is a transmembrane proteoglycan that serves as a c-receptor for the TGFβ superfamily, including growth factors such as activins and BMPs 252. Betaglycan´s extracellular domain has multiple GAG attachment sites and protease-sensitive sequences, while its short intracellular domain is rich in serine and threonine residues, allowing for phosphorylation 253. The ectodomain contains a unique zona pellucida (ZP) module that, unlike other ZP proteins, does not polymerize but instead facilitates TGFβ ligand binding 254. Betaglycan enhances the affinity of TGFβ isoforms for their receptors and is essential for TGFβ2 signaling. It also acts as a suppressor in cancer, blocking NF-κB-mediated expression of matrix metalloproteinase 2 (MMP2), which limits tumor aggressiveness 255.

##### Perlecan

It is a large, modular heparan sulfate proteoglycan (HSPG) essential for various biological processes due to its structural complexity and widespread tissue distribution 256. This 500 kDa core protein consists of five domains with homologies to several molecules, and it interacts with various receptors and ligands, making it integral to vascular and extracellular matrix biology 257. Its N-terminal heparan sulfate chains promote angiogenesis by binding and presenting growth factors like VEGFA and FGF to their receptors, while protease cleavage can release pro-angiogenic factors, impacting blood vessel formation and repair. Perlecan´s C-terminal domain V, endorepellin, functions oppositely to its N-terminal proangiogenic region by inhibiting endothelial migration and angiogenesis 258. Through dual receptor targeting of VEGFR2 and α2β1 integrin, endorepellin suppresses endothelial cell migration, induces autophagy, and alters cellular structures, impacting cancer, inflammation, and vascular pathologies 259.

##### Aggrecan

It is a vital structure proteoglycan in cartilage, forming large, resilient complexes with hyaluronan and link proteins that allow cartilage to withstand compression 260. It includes multiple domains, each serving a specific function: the G1 and G2 domains stabilize aggrecan´s attachment to hyaluronan, forming robust networks with collagen that reinforce cartilage. The central GAG-rich domain, filled with negatively charged CS and KS chains, attracts water, providing hydration and compressive resistance to cartilage 261. The G3 domain, with EGF-like and lectin elements, enables aggrecan to bind to other matrix proteins like tenascins and fibulins, enhancing structural support and mechanosensitivity 262. Genetic defects in aggrecan, such as those seen in chondrodystrophies, weaken cartilage and associated health tissues, emphasizing its role in skeletal development 263. Aggrecan also has functions in the brain, where it forms part of perineuronal nets around cortical interneurons. Here, it may aid neural maturation, stabilize synaptic connections, and protect neurons from oxidative stress, indicating roles beyond cartilage integrity in maintaining neural health 264.

##### Versican

The largest hyalecan family member has a central role in tissue organization and inflammation. Encoded by the VCAN gene, versican is structurally like aggrecan but includes unique features, such as an N-terminal IgG fold and link modules that bind hyaluronan with high affinity 265. It contains central GAGα and GAGβ domains, which are variably spliced into isoforms V0, V1, V2, V3, and a newly identified V4 associated with breast cancer 266,267. These isoforms exhibit tissue-specific expression, influencing cell adhesion and signaling, particularly in the heart, brain, and vascular tissue 268,269. Versican´s C-terminal domain includes EGF-like and lectin motifs, connecting it with cell surface glycoproteins and stabilizing supramolecular structures at the plasma membrane. Versican supports inflammation by interacting with receptors like CD44 and Toll-like receptors, facilitating leukocyte migration during tissue repair 270. Proteolytic processing by enzymes, such as MMPs and ADAMTs, modifies versican´s function, influencing cell adhesion and migration, and higher levels are linked to tumor growth and inflammation in diseases like leiomyosarcoma 271.

##### SLRPs

They are a large family of proteoglycans with 18 gene variants, known for their small protein cores and leucine-rich repeats (LRRs). They are broadly expressed in various extracellular matrices, especially around developing organs, where they shape and stabilize tissue structure 272. SLRPs are categorized into five classes based on genetic similarities, with Classes I-III containing GAG chains that help them bind to collagen and stabilize collagen fibrils, thereby protecting them from enzymatic degradation 273. The family´s best-known member, decorin, binds collagen type I and is essential for proper collagen fibrillogenesis. SLRPs also interact with various receptors and signaling pathways, including TGF-β, influencing cellular processes 274. They show structural diversity in GAG chains, allowing them to support a wide range of functions across tissues, and may be transcriptionally co-regulated, indicating a coordinated role in development and tissue repair 273. SLRPS are divided into five groups.

##### Class I SLRP

**Decorin (PG40 or DSPG1)**, the most studied SLRP, binds collagen fibrils, stabilizing tissue structure and affecting collagen´s biomechanical properties, which is important for skin integrity and connective tissues 275. Mice without decorin show weakened skin, like Ehlers-Danlos syndrome 276. Decorin also has anti-cancer properties, acting as a growth suppressor by binding to growth receptors like EGFR and Met, inhibiting tumor progression and blood vessel formation 277,278.

**Biglycan**, decorin´s close relative, has pro-inflammatory roles through interactions with immune receptors, impacting immune response and tissue injury recovery 279.

**Asporin**, another SLRP, regulates bone formation and antagonizes cartilage formation via TGF-β signaling, with genetic variations linked to osteoarthritis severity 280.

Some SLRPs, including **ECM2**, are less understood but are thought to have distinct roles based on structural homology. SLRPs´ versatile interactions in collagen assembly, immune response, and disease progression 281.

##### Class II SLRP

This class consists of five distinct SLRPs, which can be further categorized into three subgroups based on protein homology: subgroup A (fibromodulin and lumican), subgroup B (PREPL and keratocan), and subgroup C (Osteoadherin) 282. Despite their functional diversity, all Class II SLRPs share a conserved genomic structure, composed of three exons, with the largest exon encoding most of their leucine-rich repeats (LRRs). These proteins also contain a charged N-terminal region enriched in tyrosine sulfate residues, contributing to their anionic properties 283. A defining feature of these SLPRs is their keratan sulfate and polylactosamine modifications, which contribute to their role in growth factor signaling. Notably, corneal keratan sulfate binds with high affinity to FGF2 and sonic hedgehog (SHH), suggesting its involvement in morphogen gradient formation 284. Additionally, SLRPs interact with fibrillar collagens, reinforcing tissue structure and mechanical strength.

**Fibromodulin** was originally identified in cartilage. It is also notable for delaying collagen fibril formation and is necessary for maintaining ECM stability in tissues such as cartilage and tendons 285. Its N-terminal domain contains tyrosine sulfate residues, enabling dual functions: collagen cross-linking and growth factor binding, including FGF, VEGF, and various cytokines 286. It binds the same region of collagen I as lumican and is particularly important in regulating fibrillogenesis during development 287. Fibromodulin also activates the classical complement pathway, supporting immune modulation and structural stability 288.

**Lumican**, also known for its role in maintaining corneal transparency, helps organize collagen fibrils, particularly in the cornea, where it preserves the spacing necessary for visual clarity 289. Lumican also influences cancer and inflammation; in cancer, it is often upregulated in tumor stroma, especially in breast carcinomas and melanomas, and inhibits tumor progression 290. It binds neutrophils during transmigration across endothelial barriers, enhancing neutrophil response in injury and wound healing 291.

**PRELP (Proline/Arginine-Rich END Leucine-Rich Repeat Protein)** primarily functions in connective tissues near basement membranes 292. Its positively charged N-terminal domain binds heparin and heparan sulfate, creating structural links between ECM and basement membranes 293. PRELP also inhibits NF-κB signaling, which reduces osteoclast formation, making it an effective antiresorptive molecule, as seen in osteoporosis models 294. Additionally, PRELP prevents complement membrane attack complex formation, protecting vascularized tissues in inflammatory diseases 295.

**Keratocan** is essential for corneal transparency, and keratocan supports proper stromal collagen organization, which is necessary for corneal structure 296. Although mice deficient in keratocan show normal corneal openness, they have irregular collagen fibril spacing, underscoring keratocan´s role in collagen packing 297. Inflammation studies reveal that keratocan assists in chemokine gradient formation, promoting neutrophil recruitment during corneal injury and inflammation 298.

**Osteoadherin (Osteomodulin)** is highly expressed in mineralized tissue, and osteoadherin contributes to bone matrix organization 299. With numerous tyrosine sulfates and acidic residues in its N-terminal domain, osteoadherin interacts with growth factors, mimicking heparin´s binding properties 300. It facilitates osteoblast attachment, particularly via αvβe integrin, and shows a glycosylation pattern change during endochondral bone formation, suggesting a targeted role in mineralization and bone matrix integrity 301.

##### Class III SLRP

Class III consists of three closely related members: epiphycan, opticin, and osteoglycin. Unlike other SLRP classes, they have a distinct structure, containing only seven LRRs instead of the typical 10-12 found in other SLRP classes. Like Class II members, Class III SLPRs possess N-terminal consensus sequences for tyrosine sulfation, which likely serve as signals for keratan sulfate attachment during protein synthesis and post-translational modification 302.

**Epiphycan** is a glycoprotein found in epiphyseal cartilage and is the mammalian counterpart of avian PG-Lb 303. It functions in chondrogenesis, distributed throughout the growth plate during cartilage development 304. While mice with this proteoglycan show mild bone defects, epiphycan/biglycan double-knockout mice have shorter bones and develop osteoarthritis, suggesting a cooperative function 305. It is also part of the collagen IX intercom, indicating a role in growth plate organization 306.

**Osteoglycin**, also known as mimecan, was first identified in bone and later as a keratan sulfate SLRP in the cornea 307. Although multiple mRNA variants are produced from the *Ogn* genes, they generate a single protein core 308. Besides, the inhibition of this gene in mice shows increased collagen fibril diameter in the cornea and dermis, like other SLRP-related phenotypes 309. Osteoglycin undergoes proteolytic processing *in vivo*, particularly by BMP-1/Tolloid-like metalloproteinases, which enhances its role in collagen fibrillogenesis regulation. Nevertheless, this molecule is involved in myocardial integrity, cardiac remodeling, and injury response, interacting with ECM glycoproteins 310. Additionally, osteoglycin functions as an anabolic bone factor secreted by muscle cells and may serve as a predictor of cardiovascular events 311.

##### Class IV SLRP

This is a non-canonical class of the SLPRs, and it includes the following chondroadherin: 312 and nyctalopin 313.

Chondroadherin is primarily found in cartilage, linking chondrocytes to the ECM through interactions with α2β1 integrin and heparan sulfate chains 314. It also binds collagens II and VI, and mice knockout of this molecule exhibit cartilage and bone abnormalities, including disrupted collagen network assembly and weakened mechanical properties in cartilage 315,316.

Nyctalopin is unique among SLRPs as it is GPI-anchored to the plasma membrane, and X-linked- Mutations in NYX cause X-linked congenital stationary blindness, affecting night vision, myopia, and visual acuity 317. In the retina, nyctalopin interacts with TRPM1 and mGluR6, forming a supramolecular complex for visual synapse signaling 318.

##### Class V SLRP

This lesser-known group of non-canonical SLRPs consists of podocan and podocan-like.

**Podocan** was first identified due to its elevated presence in podocytes from sclerotic glomeruli in HIV-associated kidney disease 319. It is found in the glomerular basement membrane, proximal tubules, and aortic tissue, hinting at a role in vascular healing. It functions as a suppressor of smooth muscle cell migration and proliferation, potentially influencing atherosclerosis, much like biglycan 320. In podocin-deficient cells, Wnt signaling activity is heightened, whereas cells with increased podocin expression show reduced Wnt signaling, a trait observed in other SLRPs 321. Like decorin and biglycan, podocin interacts with collagen I and contributes to cell growth regulation through p21 WAF1 activation 322.

#### Metalloproteases

Matrix metalloproteinases (MMPs) are zinc-dependent endoproteases involved in ECM remodeling, which is involved in protein degradation, tissue homeostasis, and cellular signaling 323. These enzymes regulate cell proliferation, migration, differentiation, apoptosis, and angiogenesis, influencing tissue repair and immune responses 324. However, MMPs can modify bioactive molecules on the cell surface, affecting various signaling that govern cellular behavior 325. While MMP expression and activity fluctuate in normal physiological processes, such as pregnancy and wound healing, their dysregulation has been linked to pathological conditions, including cardiovascular diseases, musculoskeletal disorders, and cancer 326. In malignancies, MMPs contribute to tumor progression and invasiveness by degrading ECM barriers and facilitating cancer cell migration and metastasis 327.

Most of them are produced by a variety of tissues and cell types, including fibroblasts, osteoblasts, endothelial cells, vascular smooth muscle cells, macrophages, neutrophils, lymphocytes, and cytotrophoblasts 328. Their activity is tightly controlled by tissue inhibitors of metalloproteinases (TIMPs), which regulate ECM turnover and prevent excessive degradation 329. However, an imbalance between MMPs and TIMPs, either through increased MMP expression or reduced TIMP levels, can contribute to pathological conditions, including cardiovascular diseases, osteoarthritis, and cancer.

##### Collagenases

Collagenases (MMP-1, MMP-8, MMP-13, and MMP-18) degrade fibrillar collagens I, II, and III by first unwinding their triple-helical structure and then hydrolyzing peptide bonds 330. Their hemopexin domain is necessary for cleaving intact collagen, while the catalytic domain processes non-collagen substrates. These enzymes are important for ECM remodeling, tissue repair, and homeostasis, but their dysregulation is linked to arthritis, fibrosis, and cancer 331.

**MMP-1** is encoded in chromosome 11 and is involved in collagen degradation and inflammation 332. Its expression in inflammatory conditions and autoimmune diseases contributes to delayed wound healing 333. MMP-1 is also linked to cancer progression, with certain genetic variations associated with poor prognosis 334. Anti-fibrotic agents like stratifin and kynurenic acid influence MMP-1 expression, enhancing tissue repair and reducing fibroids 335. Overall, MMP-1 regulation affects both tissue remodeling and disease progression 336.

**MMP-8**, also known as collagenase-2 or neutrophil collagenase, is encoded in chromosome 11 and was first identified in a cDNA library from leukocytes of a leukemia patient 337. It degrades interstitial collagens I, II, and III, with its activation regulated by other MMPs like MMP-3 and MMP-10. MMP-8 appears early in dermal wound healing, and its deficiency in mice leads to delayed healing and increased inflammation 338,339. In periodontal disease, MMP-8 contributes to connective tissue breakdown, and its presence in saliva may serve as a diagnostic marker 340.

**MMP-13**, also known as collagenase-3, is encoded in chromosome 11 and efficiently degrades type II collagen 341. It is overexpressed in osteoarthritic cartilage, contributing to disease progression, and is regulated by factors like miR-411 and dietary fatty acid ratios 342. MMP-13 is implicated in lung diseases, brain astrocyte migration, and liver fibrosis, where targeted gene delivery has shown potential therapeutic effects 343. It is also frequently overexpressed in tumors, promoting metastasis, particularly in nasopharyngeal cancer 344.

**MMP-18**, also known as collagenase-4, is encoded in chromosome 12 and shares structural similarities with MMP-1, -3, and -11. It has a unique dual-cleavage activation site and is expressed in various tissues but not in the brain, skeletal muscle, kidney, liver, or leukocytes 345. MMP-18 is found in migrating macrophages and axonal growth, particularly in response to skin explants 346. Its activity is linked to ECM breakdown, influencing nerve growth and regeneration 347.

##### Gelatinases

This group includes MMP-2 and MMP-9, which share structural similarities with other MMPs but have a unique collagen-binding domain essential for gelatin degradation. They are key enzymes in ECM breakdown and have a broad range of non-matrix protein targets 348. Beyond their role in ECM remodeling, they are involved in embryonic development, angiogenesis, vascular diseases, inflammation, infections, neurodegenerative disorders, and cancer progression. Their diverse functions make them significant regulators in both normal and pathological processes 349.

**MMP-2**, also known as gelatinase-2, is on chromosome 16 and degrades collagen through a two-phase process 350. It accumulates at the cell surface via the MT1-MMP/TIMP-2 complexes, contributing to localized collagen breakdown in angiogenesis, tissue repair, and inflammation 351. MMP-2 is implicated in tumor invasion and metastasis, particularly in esophageal cancer, and targeting it with siRNA has shown promise in reducing lung cancer growth. In glioblastomas, MMP-2 interacts with α5β1 integrin, influencing IL-6/stat3 signaling and tumor progression 352.

**MMP-9**, or gelatinase-B, is a type IV collagenase located on chromosome 20 and is produced by various cell types, including epithelial cells, fibroblasts, and immune cells 353. It acts in inflammatory cell migration and tissue remodeling 354. In cholesteatoma, MMP-9 expression is elevated in tissues rather than serum, correlating with inflammatory severity 355. Moreover, it is linked to esophageal cancer progression, with its activity correlating with vascular invasion 356.

##### Stromelysins

MMP-3, MMP-10, and MMP-11, also known as Stromelysins 1, 2, and 3, share the same domain structure as collagenases but do not cleave interstitial collagen 357. MMP-3 and MMP-10 have similar structures and substrate specificities, actively degrading ECM components, particularly in proMMP activation 358. In contrast, MMP-11 has weak ECM-degrading activity and is more distantly related. Unlike MMP-3 and MMP-10, which are secreted as inactive proenzymes, MMP-11 is activated intracellularly by furin and secreted in its active form 359.

**MMP-3** is encoded on chromosome 11 and functions as a secretory endopeptidase that degrades ECM components, including various collagens, proteoglycans, elastin, and fibronectin 360,361. It can activate other MMPs involved in tissue remodeling and has been detected in cell nuclei, suggesting a role in gene regulation and apoptosis. MMP-3 is implicated in post-traumatic osteoarthritis, where fibronectin fragments upregulate its expression, accelerating cartilage degradation 362. Furthermore, it contributes to atherosclerosis, tumor growth, and metastasis 363. It may interact with neurodegeneration by inducing mitochondrial oxidative stress and neuronal death 364.

**MMP-10** is a secreted protein located on chromosome 11. It has multiple functions in various diseases, such as pulmonary fibrosis, where its serum levels correlate with lung function and disease severity 365. MMP-10 is also implicated in respiratory syncytial virus infections, peripheral arterial disease, and wound healing 366. It may contribute to tumor progression, metastasis, and liver regeneration, particularly in hepatocellular carcinoma 367.

**MMP-11** is a protease with a gene locus on chromosome 22. It is secreted in its active form after intracellular activation, unlike other MMPs, which require extracellular activation 368. MMP-11 acts in tissue remodeling during embryonic development, wound healing, and tumor invasion 369. It has been linked to poor prognosis in various cancers, including breast, esophageal, and oral squamous cell carcinoma, with its elevated expression associated with tumor progression and metastasis 370,371. However, some studies suggest it may inhibit metastasis in certain cancer models, indicating a complex role in cancer biology 372.

##### Matrilysins

**MMP-7,** or matrilysin-1, is a small protease located on chromosome 11. It appears in tissue remodeling, particularly in the uterus and following injury, by degrading ECM components and cleaving cell surface molecules like Fas-ligand and E-cadherin 373. MMP-7 has dual effects on apoptosis, either inducing or inhibiting it, and is involved in immune response in the intestine. Studies suggest its involvement in chronic tonsillitis and idiopathic pulmonary fibrosis 374. MMP-7 may also be a key factor in cancer progression, especially in oral squamous cell carcinoma 375.

**MMP-26** (matrilysin-2), or endometase, is located on chromosome 11 and shares structural similarities with macrophage metalloelastase. It is primarily expressed in the placenta and uterus, but it is found in various tumor cell lines and malignant tumors, including those of epithelial origin 376. MMP-26 exhibits broad proteolytic activity, targeting substrates like collagen, fibronectin, and gelatin, and is present in activating proMMP-9 377. As part of this, MMP-26 interacts in tissue remodeling, angiogenesis, and tumor progression, particularly in tumor invasion 378. Expression of MMP-26 is linked to granulocyte-macrophage colony-stimulating factor (GM-CSF)-induced tumor invasion and may serve as a marker for metastasis in pancreatic adenocarcinoma 379. TIMP-2 and -4 regulate MMP-26, with TIMP-4 being a stronger inhibitor 380.

##### Membrane-type MMPs

This group consists of four transmembrane MMPs (MMP-14, -15, -16, and -24) and two GPI-anchored MMPs (MMP-17 and -25). These enzymes are activated intracellularly, with active forms expressed on the cell surface. MT-MMPs feature a membrane anchoring domain and display protease activity at the cell surface, making them efficient pericellular proteolytic machines. All MT-MMPs, except MMP-17, can be active proMMP-2. MMP-14 is also capable of activating proMMP-13 on the cell surface.

**MMP-14** (MT1-MMP) is a transmembrane metalloproteinase encoded on chromosome 14, involved in extracellular matrix degradation and cell migration 381. It activates proMMP-2, contributing to tumor invasion, metastasis, and tissue remodeling 382. Elevated MT1-MMP levels correlate with aggressive cancer phenotypes, including head and neck squamous cell carcinoma and salivary gland carcinomas 383. Deficiency in MT1-MMP leads to developmental abnormalities, emphasizing its role in skeletal and connective tissue integrity. Also, MT1-MMP is implicated in atherosclerotic plaque instability 384.

**MMP-15** (MT2-MMP), encoded on chromosome 16, is a membrane-bound metalloproteinase implicated in extracellular matrix remodeling and MMP-2 activation, influencing tumor invasion 385. It is indispensable for placental vasculogenesis, as its deficiency leads to embryonic lethality in mice. In colorectal cancer, MMP-15 is upregulated during early tumorigenesis, showing stromal localization 386. However, in supraglottic carcinoma, MMP-14 appears more influential in tumor progression 387. Elevated MT2-MMP expression correlates with increased invasion in laryngeal cancer 388.

**MMP-16** (MT3-MMP) is located on chromosome 8 and is a membrane-bound protease that modulates MMP-2 and -9 activation, influencing tumor invasion and cell migration 389. Its elevated expression in melanoma and bladder cancer correlates with adverse outcomes, while miR-146a and miR-155 regulate its activity in breast cancer and cardiac progenitor cells 390,391. In neonatal lung development, MMP-16 polymorphisms impact susceptibility to bronchopulmonary dysplasia. Experimental inhibition of MMP-16 restricts cancer proliferation and enhances regenerative potential 392.

**MMP-17** (MT4-MMP), encoded on chromosome 12, is a GPI-anchored metalloproteinase distinct from MT1-MMP, lacking a cytoplasmic tail and not activating proMMP-2 393. It features a unique nine-residue insertion necessary for furin-mediated activation. Expressed in multiple tissues, including leukocytes, its role may involve modulating growth factors and inflammatory mediators like TNF-α 394. MMP-17 is overexpressed in various cancers, particularly breast cancer, and plays an important role in tumor progression 395.

**MMP-24** (MT5-MMP), encoded on chromosome 20, is a membrane-associated metalloproteinase implicated in tumorigenesis and neural plasticity 396. It facilitates proMMP-2 activation, contributing to malignancies such as glioblastoma. The brain regulates neural stem cell quiescence by cleaving N-cadherin and influences neuropathic pain by modulating sensory neuron plasticity 397. Additionally, it mediated neuro-immune interactions in nociceptive signaling, affecting thermal pain responses. Elevated MMP-24 expression in breast cancer suggests its role in tumor progression, invasion, and angiogenesis 398.

**MMP-25** (MT6-MMP), encoded on chromosome 16, is a GPI-anchored enzyme expressed in leukocytes and certain cancers 399. It exists as a disulfide-linked homodimer, with its stability influenced by cysteine residues. MT6-MMP is localized in lipid rafts and translocates to the cell surface during neutrophil apoptosis, suggesting roles in immune response and IL-8 secretion 400. It exhibits gelatinolytic activity and weak MMP-2 activation but does not promote cell migration. Elevated MMP-25 expression in glioblastomas, colon, urothelial, and prostate cancers implicates it in tumor progression 401.

##### Non-classified MMPs

**MMP-12** is a macrophage metalloelastase located on chromosome 11 and interacts in immunity, inflammation, and tissue remodeling 402,403. It facilitates interferon-α secretion, modulating antiviral responses, and contributing to asthma-related lung inflammation 404. MMP-12 disrupts the blood-brain barrier in cerebral ischemia, exacerbating brain injury 405. Its overexpression in head and neck squamous cell carcinoma correlates with extracapsular spread, making it an interest as a prognostic marker 406.

**MMP-19,** or stromelysin-4 or RASI-1, is an ECM proteinase located on chromosome 12 and is involved in tissue remodeling, fibrosis, and cancer. It regulates angiogenesis, inhibits pulmonary fibrosis, and contributes to liver fibrosis 407. In cancer, MMP-19 promotes tumor progression in gallbladder carcinoma, colorectal cancer, and NSCLC but exhibits tumor-suppressive activity in nasopharyngeal carcinoma^408^-^410^. Genetic alterations in MMP-19 have been linked to congenital optic disc anomalies 411.

**MMP-20**, also known as enamelysin, is a tooth-specific MMP encoded by a gene on chromosome 11, clustered with several other MMP family members 412. It degrades amelogenin and other enamel matrix proteins, having a role in enamel biomineralization 413. Mutations in MMP-20 lead to amelogenesis imperfect, resulting in defective enamel formation 414,415. MMP-20 also processes aggrecan and cartilage oligomeric matrix protein, contributing to tooth matrix remodeling 416.

**MMP-21**, an MMP with gelatinolytic activity located on chromosome 1, is involved in embryogenesis and cancer progression. It is highly expressed in invasive carcinomas and correlates with our prognosis in oral, esophageal, and colorectal cancers 417-419. In Merkel cell carcinoma, MMP-21 is linked to less aggressive tumors, while MMP-10 is associated with disease progression 420. In pancreatic cancer, MMP-21 marks differentiation rather than invasiveness and is upregulated by epidermal growth factor 421.

**MMP-22**, located on chromosome 1, is linked to tumor suppression and shares a locus with MMP-21. It contains domains similar to stromelysin-3 and features a Zn^+2^-binding region necessary for activation 422. Unlike the MMPs, it lacks the “cysteine switch” for autocatalytic activation. MMP-22 produces multiple mRNA variants through alternative splicing 423.

**MMP-23**, located on chromosome 1, has a unique domain structure distinct from other MMPs. It lacks a conventional signal sequence, hemopexin-like repeats, and typical MPP subclass features. Instead, it contains cysteine-rich immunoglobulin-like domains and functions as a type II membrane protein 424. MMP-23 is predominantly expressed in reproductive tissues. It is also upregulated in MDA-MB-231 breast cancer cells 425.

**MMP-27**, a stromelysin located on chromosome 11, is poorly secreted and primarily retained in the endoplasmic reticulum. It is expressed in B-lymphocytes, endometrial macrophages, and endometriotic lesions, with peak expression during menstruation 426. In cancer, MMP-27 is more abundant in high-grade breast tumors, particularly in the MDA-MB-468 cell line. Its expression may contribute to breast cancer progression. Despite the structural resemblance to MT-MMPs, MMP-27 is not an integral membrane protein and remains stored until needed 427.

**MMP-28** (epilysin) is a widely expressed MMP involved in tissue homeostasis, wound healing, and repair, located on chromosome 17 428. It is found in the epidermis and various organs and is linked with embryo implantation, cardiac remodeling, and periodontal health 429. In myocardial infarction, MMP28 deficiency impairs healing, leading to cardiac rupture. Unlike other MMPs, it is downregulated in colon cancer, suggesting a protective role. And its expression remains elevated in osteoarthritis and rheumatoid arthritis 430,431.

All MMPs are summarized in **Table 4**.

#### Glycosaminoglycans

Glycosaminoglycans (GAGs) are negatively charged, linear polysaccharides composed of repeating disaccharide units, where hexosamines are linked to hexuronic acids or galactose via O-glycosidic bonds.

Based on disaccharide composition and sulfation patterns, GAGs are classified into four main types: chondroitin sulfate (CS) and dermatan sulfate (DS), heparin and heparan sulfate (HS), hyaluronan (HA), and keratan sulfate (KS). Most GAGs contain hexuronic acids, such as glucuronic acid (GlcA) in CH, HS, and HA, or iduronic acids (IdoA) in DS and heparin, while KS incorporates galactose instead. Hexosamine units vary, with N-acetylglucosamine (GlcNAc) found in HA, HS, and KS, and N-acetylgalactosamine (GalNAc) present in CS and DS.

Except for HA, all GAGs attach covalently to protein cores in proteoglycans (PGs), forming polymerized disaccharide chains, whereas HA is a free oligosaccharide binding noncovalently to PGs. HA is synthesized at the plasma membrane by hyaluronan synthases (HASes), while other GAGs undergo biosynthesis in the Golgi apparatus, where they attach to core proteins and undergo structural modifications.

- **Chondroitin sulfate** is a common component of the ECM and cell surfaces in animals. CS chains attach to core protein, forming proteoglycans involved in diverse biological processes through interactions with growth factors, morphogens, cytokines, and adhesion molecules 432. Despite its simple disaccharide structure, CS undergoes extensive modifications, including sulfation and epimerization, leading to structural diversity 433. CS and DS often exist as hybrid chains, influencing cellular functions through specific binding sites 434. Structural studies have revealed CS-DS interactions with bioactive proteins, yet detailed saccharide sequences remain elusive 435.
- **Dermatan sulfate**, also known as chondroitin sulfate B, is a linear polysaccharide composed of disaccharide units containing GalNAc and GlcA or IdoA 436. The presence of IdoA differentiates DS from other chondroitin sulfate and contributes to its unique protein-binding properties 437. DS structure varies due to differences in chai length, sulfation patterns, and core proteins, influencing its biological interactions. It has a variety of functions in the ECM, cell function, and wound healing 438. Unlike heparin and heparan sulfate, DS has distinct expression patterns and enzymatic modifications 439.
- **Heparin** is the oldest and most widely used anticoagulant, discovered in 1926, clinically approved since 1935 440. It is derived from animal tissues, primarily pig intestinal and bovine lung, and consists of a highly sulfated GAG backbone 441. HP exerts its anticoagulant effect by binding to antithrombin III (AT), enhancing its inhibition of thrombin and other coagulation proteases 442. Beyond anticoagulation, HP interacts with various proteins, leading to additional pharmacological effects, including anti-viral, anti-tumor, anti-inflammatory, and anti-angiogenic properties 443. Recent research has explored its non-anticoagulant applications in infectious diseases 444, cancer 445, inflammatory conditions 446, Alzheimer´s disease 447, and diabetic nephropathy 448.
- **Heparan sulfate (HS)** is a widely distributed GAG found in the ECM and on cell surfaces, where it regulates tissue organization, cell signaling, and lipid metabolism 449. Structurally, HS is less sulfated than heparin and undergoes enzymatic modifications such as epimerization and sulfation in the Golgi, affecting its binding interactions with growth factors, cytokines, and ECM molecules 450. Its synthesis depends on UDP-sugar availability and is regulated by nucleotide-sugar transporters 451. HS degradation, mainly by heparanases, modifies ligand recognition and signaling, with dysregulation linked to cancer progression 452. HS proteoglycans (HSPGs), such as Syndecans and glypicans, have a role in cellular communication and structural integrity 453.
- **Hyaluronan/ Hyaluronic Acid** is a non-sulfated GAG distributed in connective and epithelial tissues 454. It is a key component of the ECM, contributing to cell proliferation, migration, and tissue hydration 455. HA consists of repeating disaccharide units of D-glucuronic acid and N-acetylglucosamine linked by β-1,4 and β-1,3 glycosidic bonds 456. Its molecular weight ranges from 10 to 1000 kDa, influencing its biological functions. HA interacts with various receptors, including CD44, RHAMM, LYVE-1, and aggrecan, in cellular signaling, wound healing, and inflammation 457. High molecular weight HA supports tissue integrity and hydration, while lower molecular weight HA is associated with inflammatory responses and scar formation 458. It is used for skin health, acting as a lubricant in connective tissues and aiding in tissue repair. HA degradation is influenced by UV exposure and inflammatory signals via toll-like receptors (TLR2-TLR4) 458.
- **Keratan sulfate (KS)** is a mucoid material containing galactose, glucose, acetyl, and sulfate groups 459. Later studies refined its classification, establishing its structural properties and protein interactions 460. KS exists in three forms: KS-I in the cornea, KS-II in cartilage, and KS-III in the brain, each differing in linkage types and sulfation patterns 461-463. It has been found to interact with regulatory proteins, including growth factors and nerve-related molecules 464

**Figure 2:**
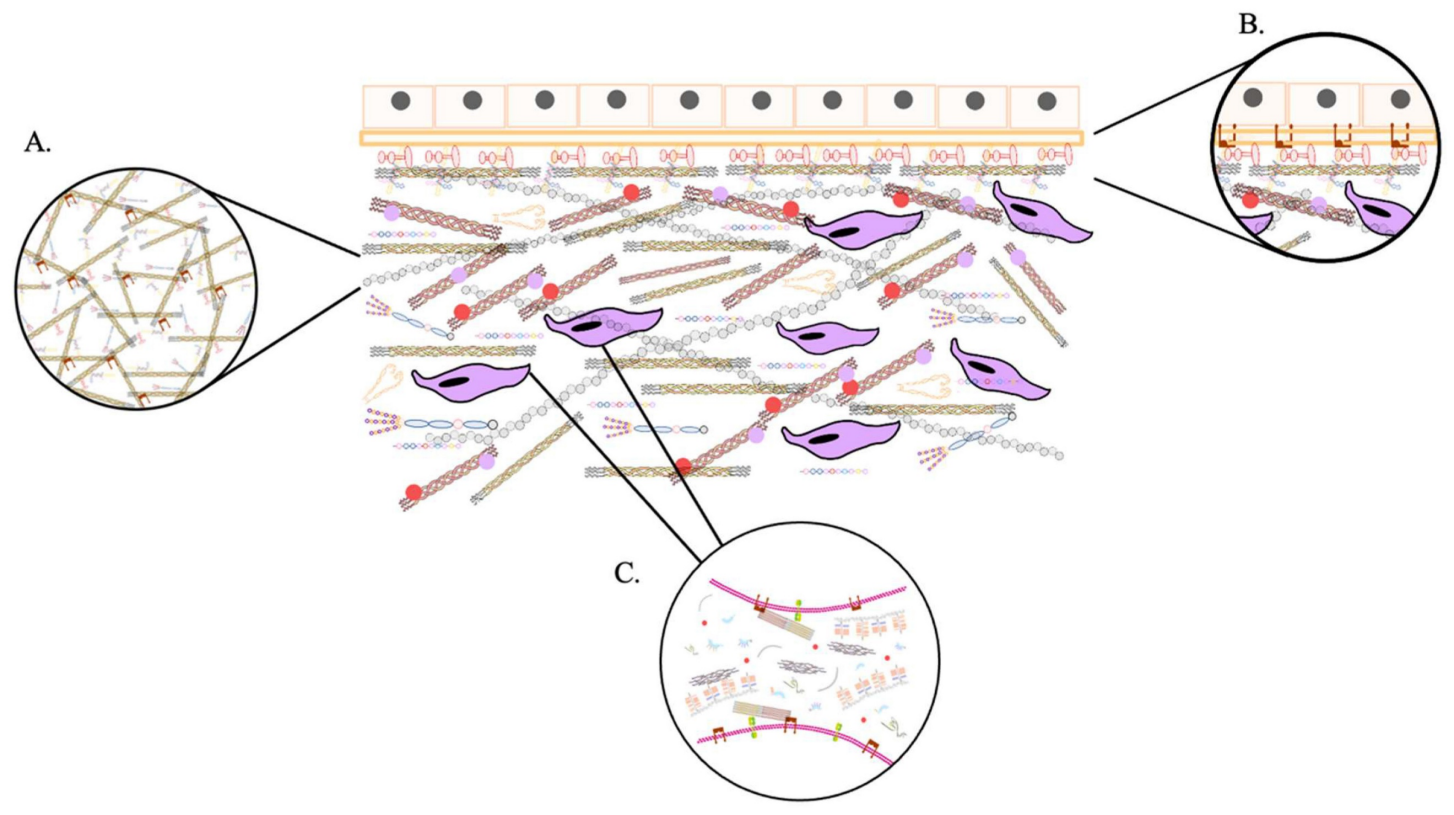
The molecules that interact in the extracellular matrix (ECM). **A.** Molecules in the intersection of the membrane and the ECM. **B.** Molecules that conform to the ECM. **C.** Molecules that are in the space between two fibroblasts.

**Figure 3:**
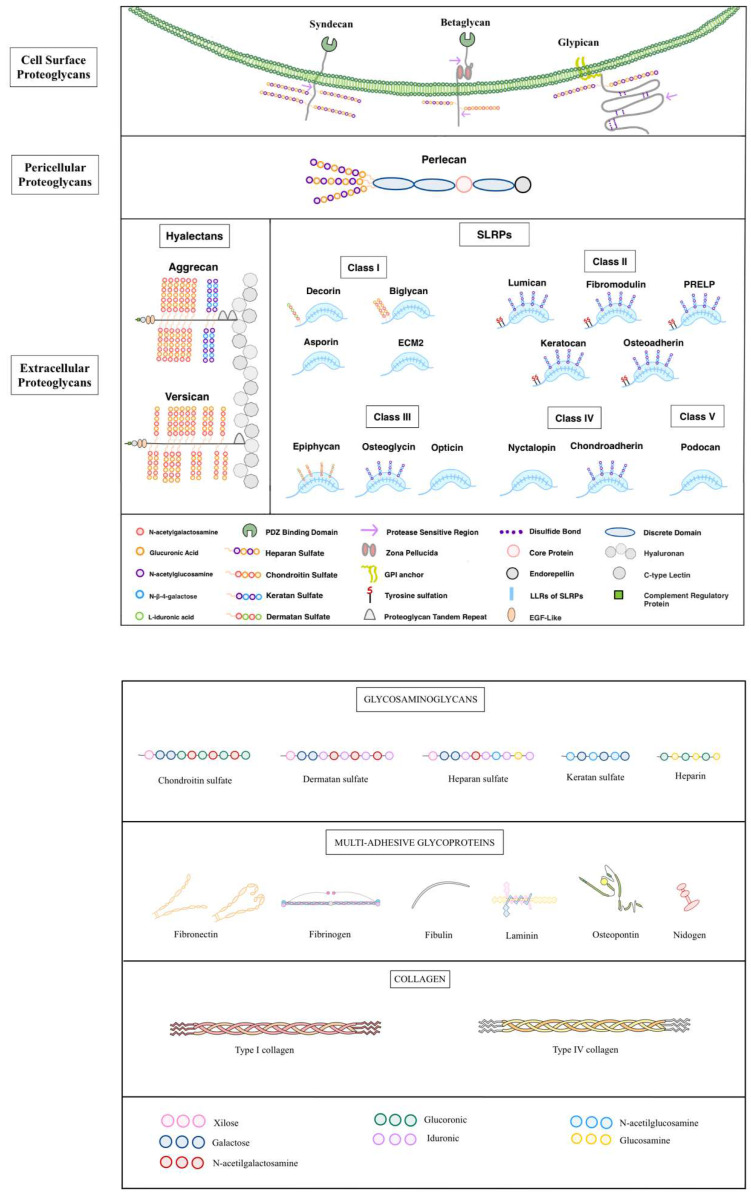
Schematic representation of extracellular matrix (ECM) components and their classification. The diagram illustrates the hierarchical organization of ECM molecules, including cell surface proteoglycans (syndecan, betaglycan, glypican), and pericellular small leucine-rich proteoglycans (SLRPs, Classes I-V). Core proteins and glycosaminoglycan (GAG) chains are indicated, with color-coded domains highlighting structural and functional motifs such as protease-sensitive regions, disulfide bonds, and glycosylation sites. Glycosaminoglycans, including chondroitin sulfate, dermatan sulfate, heparan sulfate, keratan sulfate, and heparin, are shown separately, with corresponding symbols for sugar residues. Multi-adhesive glycoproteins (fibronectin, fibrinogen, fibulin, laminin, osteopontin, nidogen) and collagens (Type I and Type IV) are depicted with their structural organization. This comprehensive schematic provides a reference framework for ECM composition, molecule interactions, and functional domains.

All molecules are summarized and represented in **Figures 2 and 3**.

## Biological functions of the extracellular matrix

Far from being a passive scaffold, the extracellular matrix (ECM) constitutes a dynamic and interactive interface between cells and their microenvironment. It continuously integrates and responds to mechanical forces and biochemical signals, thus regulating essential cellular processes, maintaining tissue organization, and influencing pathological phenomena such as fibrosis, chronic inflammation, and tumor progression (**Table 5**). Thus, the ECM is essential not only for structural integrity but also for signal transduction, homeostasis, and disease modulation.

### Structural support and mechanical integrity

The ECM provides essential mechanical support to tissues by conferring tensile strength, elasticity, and structural resilience. This is especially important in load-bearing tissues such as skin, tendons, cartilage, and blood vessels, where fibrillar collagens (especially types I and III) and elastin are primarily responsible for biomechanical properties 465,466.

Beyond mechanical reinforcement, the ECM functions as a physical scaffold that supports a wide range of cell types, including epithelial, muscle, and bone cells, anchoring them within their native tissue compartments 467. This anchoring occurs primarily through cell surface integrins, which bind to ECM ligands such as fibronectin, laminins, and collagens 468. Once bound, integrins form focal adhesions, multiprotein complexes that link the ECM to the actin cytoskeleton and serve as a hub for mechanotransduction and signal transduction pathways 469,470.

In addition, the ECM plays a critical role in maintaining epithelial polarity, orienting tissue patterning, and preserving the structural integrity of specialized compartments such as basement membranes 471. By defining the spatial organization of cells within tissues, the ECM contributes to maintaining organ function and integrity.

### Biochemical barrier and compartmentalization

The ECM also functions as a selective biochemical barrier, regulating the diffusion and local concentration of signaling molecules, nutrients and metabolites 472. This control is essential for the establishment and maintenance of tissue compartments, as well as for the orchestration of biochemical gradients that guide morphogenesis, immune responses and tissue repair.

ECM components such as proteoglycans, with their high-affinity binding sites for soluble factors, sequester growth factors and cytokines-including TGF-β, FGF, VEGF, and EGF-and release them in a controlled, spatiotemporal manner following matrix remodeling or mechanical stress 473. This regulation adjusts the local availability of bioactive molecules and modulates processes such as cell migration, proliferation, and angiogenesis.

In pathological contexts such as chronic inflammation or cancer, changes in ECM porosity and composition may facilitate or hinder immune cell infiltration and molecular diffusion, thus modulating local immune response and disease progression 474.

### Regulation of cell metabolism, differentiation, and proliferation

The ECM exerts a profound influence on cell behavior through biochemical signals and biomechanical properties (**Table 6**). The coupling of integrins and other ECM receptors activates intracellular signaling cascades - most notably the PI3K/AKT, MAPK and FAK pathways - that govern cell proliferation, metabolism, differentiation, and survival 475,476.

A critical phenomenon linked to ECM interactions is anoikis, a form of apoptosis triggered by the loss of cell-matrix adhesion 477. This mechanism serves as a safeguard against inadequate cell survival and anchorage-independent growth, especially relevant in the context of epithelial homeostasis and tumor suppression 478,479.

In addition, ECM stiffness and topography can instruct lineage specification and modulate stem cell fate through mechanotransduction 480. Cells sense changes in the mechanical properties of the ECM and transform them into nuclear signals through pathways such as YAP/TAZ, which ultimately influence gene expression programs 481.

The ECM also acts as a dynamic reservoir of soluble growth factors and morphogens, including members of the WNT, EGF, and TGF-β families, which are stored in latent forms and activated locally upon proteolytic cleavage or mechanical deformation of the matrix 482,483. This spatial and temporal control of signal availability is essential during development, wound healing, and tissue regeneration.

During embryogenesis, ECM composition provides positional information and guidance signals that regulate morphogenetic movements, cell fate decisions, and differentiation trajectories 484,485. These effects are mediated by coordinated interactions between ECM molecules and specific cell surface receptors, reinforcing the role of the ECM as a critical modulator of developmental biology 486.

The barrier function of the skin depends on the structural integrity of its cellular components and their rapid restoration after injury. Skin wound healing in adult mammalian skin is a highly coordinated multiphasic process involving hemostasis, inflammation, re-epithelialization, granulation tissue formation, neovascularization, and tissue remodeling 487,488. Under optimal conditions, re-epithelialization begins within hours and continues for several days as the basement membrane is restructured, and the epidermal surface is restored 489. This regenerative process is regulated by a complex network of signaling pathways involving growth factors, cytokines, matrix metalloproteinases, cell receptors, and ECM components 490.

### The ECM as a platform for cell migration

Cell migration is highly dependent on the ECM, which serves as both a structural substrate and a source of directional signals. During processes such as embryogenesis, tissue repair, immune cell trafficking, and cancer dissemination, cells traverse ECM networks through coordinated cycles of adhesion, traction, and detachment.

Cell-ECM interactions during migration are mediated by integrins, proteoglycans (e.g., syndecans, perlecan), and glycoproteins (e.g., fibronectin, laminin), which together regulate adhesion dynamics and cytoskeleton organization 491. Cells respond to ECM properties, such as stiffness, density, and orientation (collectively termed ECM topography), by modulating their migration speed, directionality, and mode of locomotion (e.g., mesenchymal versus amoeboid migration) 492-494.

ECM remodeling is an integral part of cell migration. MMPs, ADAM family proteases, and other matrix-degrading enzymes cleave ECM components to create migration pathways and release bioactive fragments 495-499. These degradation products, termed matrikines, can further modulate cell behavior by acting as chemotactic or angiogenic signals.

### ECM remodeling in fibrosis, chronic inflammation, and tumor progression

ECM remodeling is a tightly regulated process essential for maintaining tissue homeostasis, especially in response to physiological demands such as growth, wound healing, and regeneration. However, when dysregulated, it contributes to the pathogenesis of various chronic diseases (**Table 7**).

In fibrotic disorders, there is an excessive and often irreversible accumulation of ECM components, particularly fibrillar collagens, resulting in increased matrix stiffness, loss of tissue elasticity, and distortion of architecture 500-502. These changes impair organ function and are usually driven by activated fibroblasts and myofibroblasts, which may originate from resident mesenchymal cells, epithelial-mesenchymal transition (EMT) cells, or bone marrow-derived precursors 503,504. These cells produce large amounts of ECM proteins and secrete profibrotic factors, especially TGF-β, establishing a feed-forward loop that perpetuates matrix deposition.

Chronic inflammation further amplifies ECM dysregulation. Persistently recruited immune cells release cytokines and matrix-modifying enzymes, such as MMPs, that degrade ECM components and alter the biochemical and mechanical landscape of tissues 505-508. The resulting disorganization of the ECM can promote aberrant healing responses, immune evasion, and, over time, increase susceptibility to malignant transformation.

In cancer, the ECM undergoes extensive biochemical and biomechanical remodeling. Tumor-associated ECM is often denser, stiffer, and biochemically altered, favoring integrin clustering and activation of downstream oncogenic pathways such as FAK and YAP/TAZ 509-511. These pathways enhance cell proliferation, survival, invasion, and resistance to apoptosis. In addition, physical properties of the tumor ECM, such as altered porosity and fiber alignment, may hinder immune cell infiltration and contribute to an immunosuppressive microenvironment.

The spatial heterogeneity and molecular composition of tumor ECM define distinct niches that may modulate therapeutic responses and metastatic potential 512. Continuous remodeling through synthesis, degradation, chemical modification, and reassembly of ECM components is a hallmark of cancer progression and therapeutic resistance 513-515. Importantly, ECM protein fragments generated during remodeling (*e*.*g*., endostatin, tumstatin) can act as bioactive mediators that influence angiogenesis, inflammation, and tumor dormancy, further highlighting that the ECM is both a target and effector in pathological remodeling 516,517.

## Hidden connections: how ecm interacts with aging, calcification, and cancer

While individual roles of aging, calcification, and cancer have been extensively described, their convergence through ECM dysregulation remains less clearly represented. Strengthening these correlations is essential to understanding how common alterations in the matrix can simultaneously drive tissue degeneration, mineral deposition, and tumor progression.

**Figure 4:**
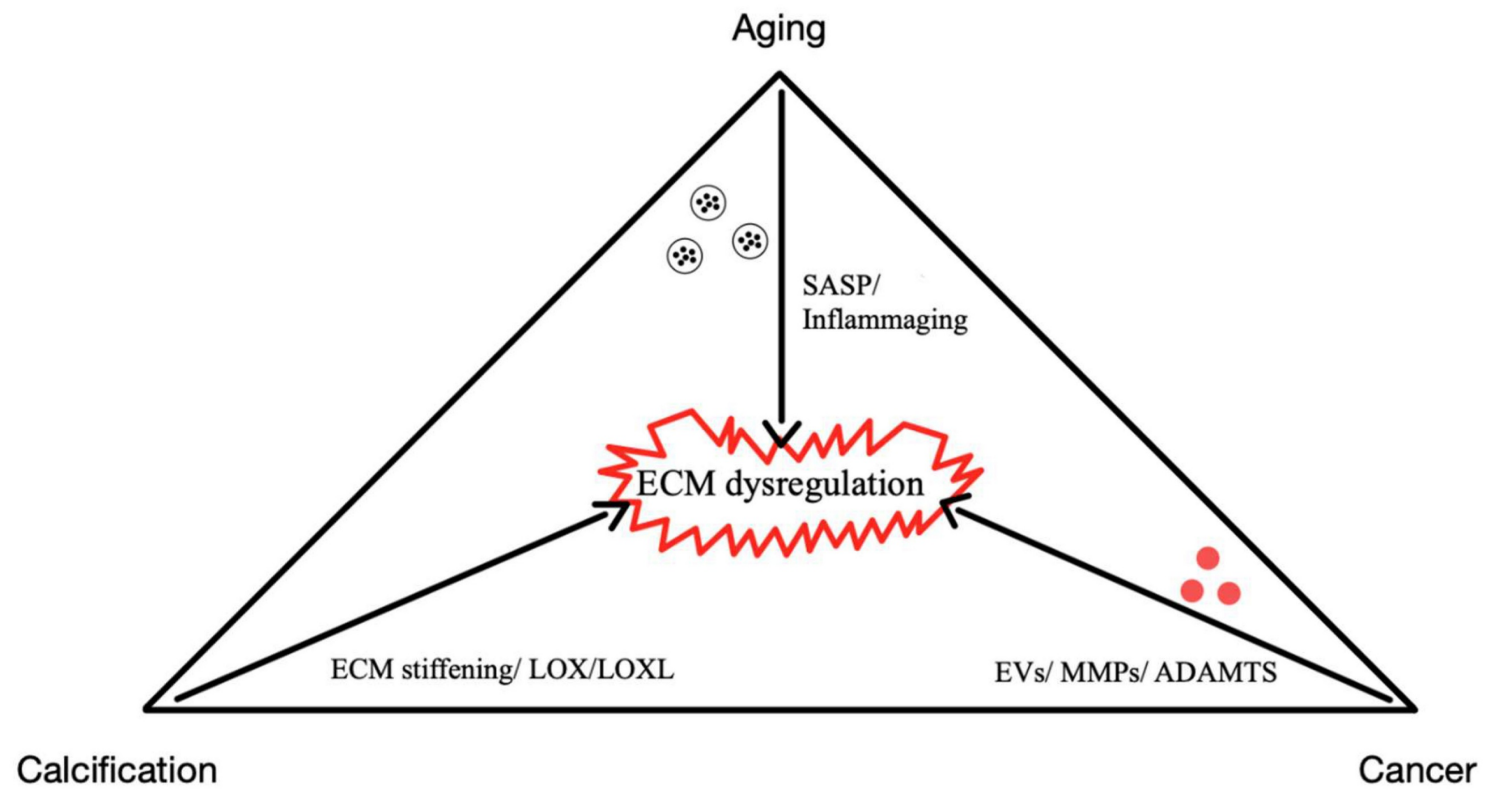
Schematic representation of the interconnections between aging, calcification, and cancer through ECM dysregulation. The diagram illustrates how ECM alterations act as a central hub linking these three processes. Although aging, pathological calcification, and cancer present distinct pathological features, they share common mechanisms such as ECM stiffening driven by LOX/LOXL crosslinking, chronic inflammation and SASP activity, as well as EVs and protease-mediated remodelling. By placing ECM dysregulation at the cancer, the figure highlights the complex and often difficult-to-visualize relationships that underlie tissue dysfunction and disease progression.

To address this, it is summarized in the following table the principal ECM changes and shared mechanisms across these conditions (**Table 8**). In parallel, a schematic model is provided to visualize ECM dysregulation as the central hub linking these dysfunctions, offering a clearer perspective on their interconnected nature (**Figure 4**).

### Interaction of ECM and aging

The extracellular matrix (ECM) is a dynamic and versatile network that serves as a key regulator of fundamental cellular functions 518. It guides cells on how to organize their structure, including adhesion and polarity; when to divide (proliferation) migrate, or undergo programmed cell death (apoptosis); where to release specific molecules (secretion); how to differentiate into specialized cell types; and how to interpret and respond to external signals 519. Dysfunction of the ECM is linked to various age-related diseases and disorders, making it a valuable target for therapeutic approaches aimed at treating conditions such as fibrosis and cancer 520.

Aging is a widespread process marked by the progressive accumulation of biological changes that gradually impair an organism´s functionality over time 521. In humans, aging is associated with a steady decline in both cognitive and physical abilities, alongside an increased susceptibility to different diseases, including cancer, diabetes, cardiovascular disorders, musculoskeletal conditions, and neurodegenerative illnesses 522.

These age-related impairments have a bad impact on quality of life, leading to disability, increased morbidity, and a heightened risk of mortality. Aging places a significant burden on individuals, their families, and society 523. The interaction between the genetic development of an individual, environmental influences, and the stochastic nature of damage accumulation determines susceptibility to age-related diseases 524. Several biological processes have been identified as hallmarks of aging, including genomic instability, dysregulated nutrient sensing, mitochondrial dysfunction, stem cell depletion, disrupted cellular communication, and excessive cellular senescence 525.

During aging, the structural integrity of the extracellular matrix deteriorates due to the accumulation of fragmented collagens, oxidation, glycation, and aggregated proteins 526. These changes impair ECM dynamics, leading to tissue fibrosis. The stiffness of the ECM also increases with age because of the gradual formation of enzymatic and nonenzymatic intramolecular and intermolecular covalent bonds between slowly turning over molecules, such as fibrillar collagens and elastin 527. Interestingly, inhibiting the cross-linking of LOXL2 and LOXL3 has been shown to restore normal collagen fibrillogenesis, reducing tissue stiffness 528. This suggests that targeting collagen cross-linking can help maintain tissue mechanohomeostasis, limiting the self-perpetuating effects of ECM stiffness on fibrosis and aging.

Also, protein modifications often involve the cleavage of peptide bonds, a process predominantly driven by enzymatic activity 529. While peptide bond cleavage and protein degradation are components of normal protein homeostasis, this balance can be disrupted in aging tissues, where degradation may outpace synthesis 530. Furthermore, partially cleaved proteins can persist within the ECM due to physiological or age-related crosslinking, leading to the accumulation of dysfunctional proteins.

Some studies have hypothesized that aging results from the accumulation of cellular and molecular damage caused by the failure of the repair mechanism. This damage is thought to occur randomly, which could explain the variability in aging phenotypes observed, even among genetically identical individuals 531.

Besides these factors that accelerate aging in ECM, the accumulation of senescent fibroblasts with age (a more detailed explanation is provided in the following sections) is ongoing, about whether SASP-driven effects on tumor development are directly attributable to aging 532. Interestingly, long-lived species such as lobsters and rainbow trout retain telomerase activity, allowing continuous cellular proliferation, which may contribute to slower senescent cell accumulation 533,534. However, direct evidence supporting this hypothesis remains limited.

Senescence is not a uniform process, and different triggers, including oncogenic activation, replication stress, or environmental factors, can alter the composition of SASP factors 535. Thus, not all forms of senescence necessarily reflect aging-related decline. In aged tissues, a marked reduction in fibroblast density and proliferative capacity has been observed, yet this loss is highly localized rather than uniformly distributed 536. Studies in aging mouse skin suggest that fibroblasts do not compensate for this loss by proliferating but instead extend their membrane protrusions to maintain tissue architecture 537.

Beyond numerical decline, aged fibroblast undergoes significant phenotypic change. Recent findings indicate that fibroblasts in aged skin exhibit genomic reprogramming, shifting from ECM-producing cells toward a more adipogenic-like phenotype 538. Notably, this transformation is metabolically regulated, caloric restriction suppresses this age-related shift, whereas a high-fat diet accelerates it.

**Figure 5:**
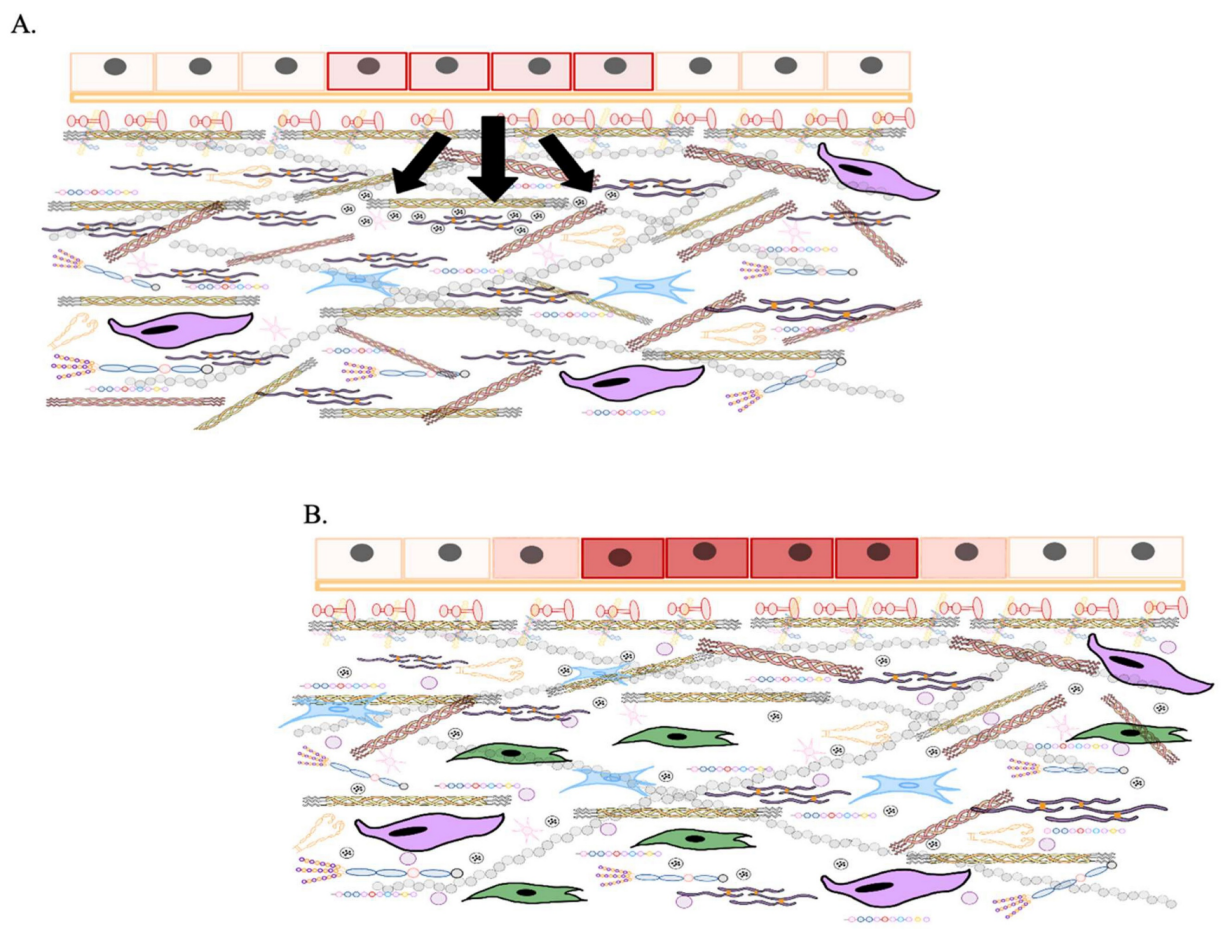
** A.** Representation of an extracellular matrix (ECM) before undergoing degradation due to factors associated with aging and senescence. The ECM contains normal levels of collagen and elastin and factors characteristic of the senescence-associated secretory phenotype (SASP). **B.** Representation of an aged and senescent ECM, showing a reduction and fragmentation of elastin fibers, a lower amount of collagen fibers, the presence of senescence-associated fibroblasts, and the dispersion of molecules synthesized and released into the extracellular matrix by the senescence-associated secretory phenotype (SASP). These molecules promote the senescence of nearby cells near the primary senescent cells.

Aging is a significant factor that leads to a decline in human health, contributing to the development of various illnesses, including tissue calcification and tumors. This section explores the different pathways through which aging impacts the activity of the ECM. All these concepts are observed in **Figure 5**.

#### Vascular aging

The fragmentation of elastic fibers is a hallmark of aging in tissues like skin and blood vessels. Vascular aging is characterized by both structural and functional changes in the arteries, often accompanied by increased thickening of the intimal and medial layers of the arterial wall 539. Alterations in the composition and organization of ECM components primarily drive these changes. Among the various molecular factors that accumulate with age, advanced lipid peroxidation end products (ALEs) are particularly notable 540. These byproducts are closely linked to oxidative stress, with a role in diseases such as atherosclerosis. Aldehydes, produced through the peroxidation of polyunsaturated fatty acids (PUFAs), including 4-hydroxyphenyl (4-HNE), malondialdehyde (MDA) 541, and acrolein 542, can form adducts with cellular proteins. This leads to progressive protein dysfunction, contributing to the complex pathophysiology of vascular aging 543. Despite the established role of aldehydes in protein modification, their specific impact on ECM components remains unclear 544. In one study using immunohistology and confocal immunofluorescence techniques, the research showed that 4-HNE-histidine adducts accumulate in an age-dependent manner across all layers of the human aorta, including the intima, media, and adventitia 545. These adducts are predominantly localized in vascular smooth muscle cells (VSMC). Interestingly, while elastin fibers in aged vessels exhibit significant structural alterations, the study demonstrated that elastin itself is minimally affected or only weakly modified by 4-HNE. For instance, young human aortas exhibit thick, organized elastic fibers arranged in parallel 545. In contrast, older aortas show thinner, fragmented, and disorganized fibers interspersed with an increased presence of other ECM components. These findings establish a complex union between lipid peroxidation and ECM components, suggesting that while aldehyde adducts have a role in vascular wall remodeling and the progression of atherosclerosis, their direct impact on elastin integrity appears limited 545.

While Sox9, a transcription factor, is known to influence VSMC differentiation into osteo/chondrogenic lineages, its connection to aging and calcification remains unexplored 546. Recent research examined human aortic samples and senescent VSCMs to assess Sox9 expression and its impact on ECM properties. Sox9 was not directly linked to vascular calcification but was strongly correlated with cellular senescence markers like p16 547. Mechanosensitive responses revealed increased Sox9 expression and nuclear translocation in aged cells and stiff ECM environments. Notably, Sox9 altered ECM stiffness and organization by modulating collagen expression and reducing VSMC contractility, leading to an ECM profile characteristic of senescent cells 547. An important discovery was the role of procollagen-lysine, 2-oxoglutarate 5-dioxygenase 3 (LH3), as a Sox9 target, mediating ECM stiffness through its secretion in extracellular vesicles. Experimental manipulation demonstrated that Sox9-induced ECM stiffening promotes VSMC senescence, while ECM synthesized from Sox9-depleted cells rescued senescence and restored proliferation 547.

Similarly, in the dermis, long elastic fibers characteristic of younger tissue becomes shorter and more fragmented with age 548. While mechanical fatigue has been proposed as a direct cause of elastic fiber damage 549, studies on porcine aortic elastic fibers subjected to repetitive cyclic loading *in vitro* have demonstrated fatigue-related damage 550. However, mechanical fatigue alone does not account for all observed changes. Notably, increased activity of MMPs with elastase activity in the aortic walls of both rodents and humans suggests a significant role for enzymatic degradation in elastin fragmentation 551.

#### Alterations of collagen by the effects of aging

In tendons, the ECM provides biochemical signals for tissue growth, repair, and healing, and a physical scaffold to support the biochemical properties of the tendons 552. However, aging induces significant changes in tendon ECM composition and organization, impacting its function and structure 553. Collagen accounts for 60-85% of the dry weight in tendons and is central to their tensile strength and viscoelasticity 554. Collagen type I dominates, with smaller contributions from type III and V, which regulate fibrillogenesis and alignment 555. With age, collagen biosynthesis declines due to reduced activity of collagen-secreting tenocytes and tendon stem/progenitor cells (TSPCs) 556. At the same time, remodeling enzymes, oxidative stress, and advanced glycation end products (AGEs) contribute to collagen fragmentation, disorganization, and cross-linking 557. These processes lead to diminished fibril size, altered fibril alignment, and decreased ECM functionality, ultimately compromising the mechanical performance of the tendons 558. Enzymes such as MMPs and their inhibitors (TIMPs) have a pivotal role in ECM remodeling. Aging tends to disrupt the balance between MMP and TIMP activity, favoring excessive collagen degradation 559. For example, increased activity of collagenases (e.g., MMP-1, MMP-13) and gelatinases (MMP-2, MMP-9), combined with reduced TIMP levels, accelerates ECM turnover but also results in disorganized collagen and impaired tissue repair 560,561. Chronic upregulation of MMP activity has also been linked to tendon degeneration and pathologies 562. Studies on equine flexor tendons have revealed elevated levels of neo-epitopes (markers produced by the cleavage of collagen´s triple helix by MMP-1 and MMP-13 in older horses) 563. Also, the expression of MMP-10 at the RNA level and MMP-3 at the protein level increased in aging tendon tissue 564.

AGE formation, which occurs through the non-enzymatic binding of sugars to collagen, further exacerbates the decline in ECM integrity during aging 565. AGEs increase collagen stiffness, disrupt collagen-proteoglycan interactions, and impair cell adhesion and migration 566. Hyperglycemia and oxidative stress from senescent cells accelerate AGE accumulation, leading to a more rigid and less functional ECM. Additionally, the long half-life of tendon collagen allows AGE-related cross-linking to accumulate over time, further hindering ECM homeostasis and tendon regeneration 567. Overall, aging leads to profound alterations in tendon ECM composition and structure, reducing its biochemical properties and repair capacity.

The relationship between aging and tendon biomechanics has been extensively studied in human and animal models. Still, findings are often inconsistent due to differences in study design, subject age, tendon type, and conditions 568.

One of the defining features of tendon aging is the gradual and often irreversible decline in its mechanical properties 569. This deterioration is largely driven by changes in tendon cellularity, collagen turnover, fibril diameter and alignment, and collagen cross-linking, particularly glycation-induced cross-linking. Key biochemical parameters used to evaluate tendons include tensile strength, modulus, stiffness, and viscoelasticity 570. These metrics, while interrelated, reflect distinct properties: stiffness measures resistance to deformation under force, while strength quantifies the maximum stress a tendon can endure before permanent damage or failure 571, determining whether a tendon is both string and elastic or strong yet rigid. Proteomic analyses have uncovered age-related neo-peptides derived from collagens and glycoproteins in older equine tendons, absent in younger tissue, suggesting shifts in ECM breakdown patterns with age 572. A key focus moving forward is identifying and driving the production of these neo-peptides, as they represent a therapeutic target for minimizing ECM fragmentation in aging tissues. Generally, various studies associate aging with unchanged or reduced tensile modulus, stiffness, and strength. For instance, some research has shown minimal differences in maximum strength in aged human tendons or mouse models 573. In contrast, others report reduced stiffness and modulus in the Achilles tendon of older adults 573. However, contradictory evidence exists; one study noted stiffer Achilles tendons in elderly participants compared to younger individuals.

#### Alterations in the production of cartilage caused by aging

The ECM of cartilage has been studied extensively due to its connection to osteoarthritis, a degenerative condition associated with aging 574. Proteoglycans, such as aggrecan, gradually decline in cartilage and are subject to enzymatic cleavage over time 575. Aggrecan is broken down between its G1 and G2 globular domains by enzymes like MMP-13 and ADAMTS5, with these fragments accumulating in older tissue 576. The G3 domain of aggrecan, located at its C-terminal end, also diminishes with age 577. Similarly, type II collagen, another key structural component of cartilage, undergoes denaturation and fragmentation in aging cartilage, undergoes denaturation and fragmentation in aging cartilage 578.

These observations illustrate the complex processes of ECM degradation across various tissues as they age, emphasizing the importance of further research to uncover the enzymes and pathways responsible. Such insights could enable the development of targeted treatments to maintain ECM integrity and reduce age-related tissue degradation 579.

#### The role of senescent cells in ECM production

Cellular senescence is a hallmark of aging, characterized by growth arrest and profound changes in gene expression, particularly through the senescence-associated secretory phenotype (SASP) 580. While it remains difficult to determine whether senescence is driven primarily by aging or external factors, a defining characteristic is the progressive accumulation of senescent cells in aged tissues and stromal microenvironments 581.

It serves as a double-edged sword in physiological processes, providing both protective and detrimental effects depending on its duration and regulation 582. In its beneficial role, senescence contributes to tissue repair, tumor suppression, and the resolution of fibrosis, ensuring organismal integrity and even playing a role in embryonic development 583. Under normal conditions, the immune system efficiently clears senescent cells, preventing their excessive accumulation and maintaining tissue homeostasis 584.

However, as aging progresses, the immense system becomes less effective at removing senescent cells, leading to their prolonged persistence and accumulation 585. This contributes to chronic inflammation, impaired wound healing, and increased susceptibility to tumor progression, turning what was once a protective mechanism into a driver of aging-related diseases 586. This phenomenon, known as antagonistic pleiotropy, illustrates how biological mechanisms that are beneficial in early life can become detrimental with age 587.

The mechanisms behind this accumulation remain debated. One hypothesis suggests that age-related immune cell decline reduces the clearance of senescent cells, leading to their persistence 588. However, some studies challenge this notion, arguing that the evidence supporting impaired immune surveillance as the primary cause remains inconclusive 589. Regardless of the mechanics, senescent cells actively modify their environment by secreting proinflammatory cytokines, chemokines, growth factors, and matrix-degrading enzymes 590. This secretory phenotype has dual effects: it can signal immune cells to clear damaged cells but also promote chronic inflammation, tumor progression, and ECM remodeling 591,592.

Traditionally, DNA damage has been considered a major trigger for age-related senescence. However, emerging research suggests that epigenetic alterations and chromatin remodeling play equally significant roles 593. For instance, histone deacetylase (HDAC) modulation in tumor-associated fibroblasts can induce SASP without direct DNA damage 593.

These findings enhance the complex regulatory network governing senescence, emphasizing that genomic instability, epigenetic modifications, and immune dysfunction contribute to senescent cells' persistence 594.

Notably, while increased ECM stiffness may contribute to the development of cellular senescence during aging and chronic fibrotic diseases, ECM derived from young human fibroblasts has been shown to restore a more youthful state in aged, senescent cells 595. As ECM stiffness increases, the fibrotic process is further driven by excessive secretion of TGFβ, YAP-1, and its paralog WW domain-containing TAZ 596. YAP and TAZ function as mechanotransducers, activating the expression of pro-fibrotic genes, such as transglutaminase-2 and lysyl oxidases 597.

However, the relationship between aging and YAP/TAZ signaling is complex and tissue-specific 598. For instance, genetic inactivation of YAP/TAZ in stromal cells accelerates aging, while maintaining YAP activity has been shown to rejuvenate aged cells and prevent age-related changes by mitigating inflammaging 598.

Elastin, an important ECM protein (the most abundant in the lung), has an important role in the formation and structural integrity of alveoli in mice. As a key component of the alveolar architecture, elastin becomes severely dysregulated in chronic obstructive pulmonary disease (COPD), an age-related lung disorder characterized by progressive airflow limitation 599. This pathology is closely linked to the accumulation of senescent cells and a diminished proliferative capacity of mesenchymal precursor cells in the alveolar parenchyma. Together, these factors significantly impair ECM production, including elastin synthesis 599. A prominent feature of COPD is emphysema, marked by the abnormal expansion of alveolar spaces and extensive remodeling of lung parenchyma. Evidence suggests that emphysema arises from reduced production and inadequate regulation of ECM components necessary for tissue repair and maintenance 600. This decline in ECM remodeling disrupts the homeostasis of connective and epithelial tissues, leading to structural insufficiency 601. Cellular senescence appears to be a central driver of these changes, primarily through its impact on mesenchymal cell function 602. The resulting decline in ECM protein production, including elastin, exacerbates tissue dysfunction and the role of elastin in preserving normal lung mechanics 601. These findings emphasize the destructive consequences of senescence-associated elastin dysregulation, providing valuable insights into the progression of COPD and its underlying mechanisms 603.

The accumulation of senescent fibroblasts significantly contributes to skin aging 604. These fibroblasts lose their cellular identity, exhibit an increased release of the senescence-associated secretory phenotype (SASP) (**Figure 3**), and disrupt the balance of ECM homeostasis 605,606. This process triggers a cascade effect as senescence spreads between cells, perpetuating dermal aging 607. The aged dermis displays prominent clinical characteristics, including decreased thickness, reduced resilience, and diminished mechanical strength, which results in a wrinkled and flabby appearance 608. These changes are associated with the loss of ECM components during aging, driven by reduced synthesis and increased degradation of ECM in aged fibroblasts. SASP components, such as MMPs, which directly cleave collagen fibrils, significantly contribute to ECM degradation during aging 609. Research has demonstrated that levels of MMPs, including MMP1, MMP2, and MMP9, are elevated in the aged dermis and fibroblasts derived from older individuals 610. Overexpression of hMMP1 induces aging phenotypes in *ex vivo* 3D human skin organ culture and *in vivo* mouse models. Also, increased MMP levels are accompanied by reduced TIMPs in aged skin, leading to an imbalance in the MMPs/TIMPs ratio and progressive collagen fragmentation 611. Overexpression of TMP-1 has been shown to protect ECM integrity and maintain elasticity under chronic UVB exposure, whereas neutralizing TIMP-1 produces the opposite effect 612.

Furthermore, fibroblasts in aged skin exhibit reduced production of ECM, particularly the key collagen network components such as collagen types I and III 613. This decline is attributed to reduced TGF-β signaling. Multiple studies enhance the importance of TGF-β in dermal aging. Physiologically, the aged dermis shows reduced ECM content 614. TGF-β signaling promotes ECM gene expression while suppressing ECM degradation by downregulating MMPs and upregulating TIMPs 615. Oxidative stress or UV irradiation can disrupt the TGF-β signaling pathway in fibroblasts, leading to decreased expression of downstream targets such as connective fibers growth factor (CTGF/CCN2) and type I collagen 616,617. Knockdown of TβRII or Smad3 reduces collagen synthesis, while overexpression of TβRII restores UV-induced collagen loss by activating TGF-β signaling 618. Disrupted TGF-β signaling also alters CCN1 expression in dermal fibroblast mice, mimicking aged skin characterized by a wrinkled appearance and disrupted collagen network 618. Reduced fibroblast size (a hallmark of dermal fibroblasts in aged skin) is associated with decreased TβRII expression and diminished ECM production in aged human skin 619. Thus, a compromised TGF-β signaling pathway is closely linked to dermal aging 620.

### Interaction of ECM and calcification

Calcification is the process by which calcium accumulates in tissue, leading to the deposition of calcium salts through the crystallization of phosphate ion (PO_4_^3-^) and ionized calcium (Ca^+2^) 621. While this mechanism is vital for the development and mutation of bones and teeth, pathological calcification can occur in nearly all soft tissues, often associated with aging and diseases 622. In bone physiology, calcification is fundamental for skeletal growth and mechanical strength. This process involves precisely depositing calcium phosphate crystals within a protein matrix, gradually forming a rigid and durable skeletal framework 623. The regulation of this process is orchestrated by a complex network of osteoblasts, osteoclasts, stem cells, and signaling molecules, ensuring the production of robust bone tissue. The composite structure of mammalian bones and teeth reflects a sophisticated combination of inorganic and organic components 624. These tissues have approximately 70% inorganic minerals, 20% proteins, and 10% water by weight 625. The inorganic crystals confer resistance to compressive forces and enhance tissue toughness by interacting with the protein matrix. Type I collagen, the primary in this matrix, provides tensile strength and is a scaffold for hydroxyapatite deposition, the main inorganic component 625.

Aging is the leading driver of pathological calcification, though this process is also frequently observed in tumors, blood vessels, and joints 626. Pathological calcification can occur through various pathways, with differing levels of cellular regulation, particularly in non-skeletal tissues such as the vasculature and neoplasms 627. Calcified deposits, composed of calcium phosphate crystals embedded within the extracellular matrix, can have detrimental effects on tissue function. These deposits contribute to mechanical stress and stiffness, impairing the flexibility and resilience of affected tissues 628. Additionally, they are associated with cellular damage and inflammation, further exacerbating tissue dysfunction. Despite their adverse effects, calcified deposits are commonly utilized as markers for diagnosing and tracking disease progression 629.

The formation of calcium phosphate crystals can occur through different pathological mechanisms, leading to distinct types of calcifications 630. Dystrophic dysfunction arises in areas affected by trauma or necrosis despite normal plasma levels of calcium and phosphate 631. This process can result from various factors, including mechanical injury, inflammation, injections, or parasitic infections, leading to localized calcium accumulation 632. In contrast, metastatic calcification develops due to systemic disturbances in calcium and phosphate homeostasis 633. Conditions such as hypercalcemia drive calcium deposition in previously healthy tissues, leading to widespread calcification in multiple organ systems 634.

Also, iatrogenic calcification can occur as an unintended consequence of medical interventions 635. Surgical procedures, radiation therapy, or the administration of calcium or phosphate-containing agents can trigger pathological calcium deposition 636. Although abnormal calcification can affect almost any soft tissue in the body, certain areas are particularly susceptible, including blood vessels, heart valves, the brain, breasts, kidneys, gastric mucosa, lungs, and tendons 637-640. Among these, vascular calcification has been a primary focus of research due to its significant clinical implications.

**Figure 6:**
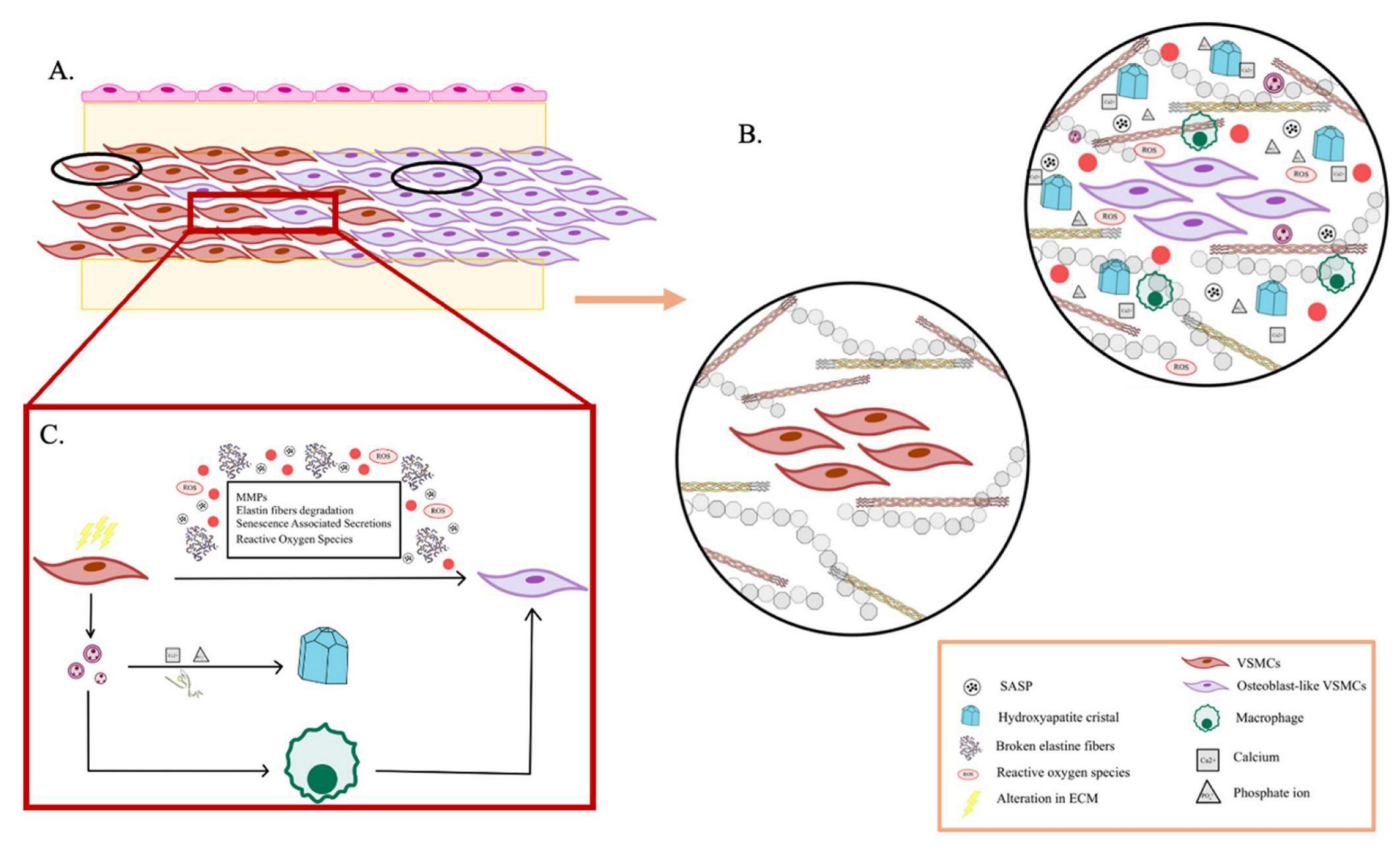
** A.** Generic image of the muscle cell calcification process. **B.** Enlarged view of the general image. The left image shows the physiological conditions of the extracellular matrix (ECM) and its interaction with the cell. In contrast, the right image illustrates changes in ECM conditions, where calcification occurs due to ECM alterations. This process begins with the deposition of hydroxyapatite crystals mediated by extracellular vesicles. The accumulation of reactive oxygen species (ROS) promotes this mineralization. These pathological changes are exacerbated by ECM remodeling due to an increase in the presence of matrix metalloproteinases (MMPs), which degrade the ECM, along with other products associated with the senescence-associated secretory phenotype (SASP). **C.** Calcification process of a muscle cell induced by extracellular matrix (ECM) alterations. The increase in matrix metalloproteinases, the degradation of elastin fibers, and the accumulation of reactive oxygen species create a pro-inflammatory environment that promotes cellular dysfunction. In response, muscle cells release extracellular vesicles rich in calcium and inorganic phosphate, promoting the nucleation of hydroxyapatite crystals through proteins such as osteopontin. Additionally, these vesicles activate macrophages, which amplify inflammation and contribute to the destruction of healthy muscle tissue, further exacerbating the calcification process.

Vascular calcification refers to the pathological deposition of calcium salts within blood vessels, primarily affecting the intimal and medial layers as well as the aortic valves. Initial calcification is strongly associated with atherosclerosis, where calcium phosphate deposits form within the ECM rather than inside cells 641. This process is commonly linked to atherosclerosis plaques, leading to vascular stiffening and increased cardiovascular risks. The mineral composition of vascular calcifications varies, with depositions consisting of apatite, whitlockite, or octacalcium phosphate, ranging in size from submicron particles to formations exceeding 0.5 mm 642. As vascular calcification progresses, it disrupts ECM homeostasis, contributing to arterial stiffening and vessel rupture, both of which significantly impact cardiovascular tissue 643. **Figure 6** shows the main intervention of calcification and its interaction with ECM.

#### Calcific aortic valve disease

Calcific aortic valve disease (CAVD) is a progressive disorder that ranges from aortic valve (AoV) sclerosis to aortic stenosis (AS), characterized by extensive calcification and impaired leaflet function 644. It is the most prevalent valvular heart disease in developed countries, particularly affecting aging populations, with a prevalence of up to 13% in individuals over 65 years old 645. The development of CAVD is influenced by multiple risk factors, including hypertension, smoking, diabetes mellitus, hypercholesterolemia, and male sex. In addition to these well-established contributors, genetic and developmental factors have a significant role 646. For instance, bicuspid aortic valve (BAV), a common congenital heart defect, has been linked to CAVD through dysregulation of RUNX2 expression, a key regulator of osteogenic differentiation 647.

The progression of the disease follows a continuum, beginning with valve sclerosis, followed by chronic inflammation, and culminating in pathological calcification, which results in aortic stenosis 648. This pathological process is orchestrated by a dynamic interplay of cellular components, including valve interstitial cells (VICs), vascular endothelial cells (VECs), and inflammatory cells, which collectively mediate ECM remodeling 648. An abnormal ECM remodeling is a key hallmark of CAVD. In a healthy air valve, ECM integrity is maintained through complex signaling interactions between VICs and VECs, with additional contributions from circulating mesenchymal cells, innate immune cells, and extracellular vesicle release 649. The ECM composition within the aortic valve varies across its internal layers, each serving distinct mechanical functions. The outflow layer, known as the fibrosa, is rich in type I and III collagen bundles, which provide the tensile strength necessary to withstand mechanical stress 650. In contrast, the inflow layer to the ventricles contains an advanced concentration of radially oriented elastic fibers, which have an important role in ensuring proper valve closure at the end of the diastolic phase and in absorbing mechanical pressure fluctuations throughout the cardiac cycle 651. Disruptions in this finely tuned ECM organization contribute to the pathological progression of CAVD, leading to compromised valve function and calcification 652.

This pathological calcification occurs through two primary mechanisms: dystrophic calcification, driven by passive hydroxyapatite deposition, and biomineralization (ossification), an active, cell-mediated process 653. Microscopic, calcified nodules frequently emerge near sites of endothelial damage, where aggregated cell debris and degraded ECM fibers serve as substrates for calcium and phosphate deposition 654. Within these nodules, apoptotic cells further contribute to mineral accumulation 655,656. Myofibroblasts also have a role due to their contractile properties increasing in response to stiffer ECM environments, promoting apoptosis. Meanwhile, oxidative stress has been linked to immune cell apoptosis within the inflamed aortic valve, although the molecular pathways driving ECM mineralization remain poorly understood 657.

Emerging evidence suggests that extracellular vesicles (EVs) are involved in valve mineralization, with two key subtypes identified: mineralized spheroid microparticles and matrix vesicles (MVs) 658. Mineralized spheroid microparticles (1-3 µm in size), first described in 2013, have been associated with microcalcifications characteristic of dystrophic calcification 659. VIC cultures undergoing osteogenesis differentiation exhibit these microparticles on their surface, similar to observations in human calcified aortic valves. Although the precise mechanism of their secretion remains unclear, their presence is closely linked to VIC apoptosis and osteogenic transformation 658. In parallel, MVs, which are enriched in MMPs, likely mediate ECM remodeling and mineralization 660. Notably, these vesicles overexpress ectonucleotidases such as alkaline phosphatase (ALP), ectonucleotide pyrophosphatase/phosphodiesterase 1 (E-NPP1), and 5´-nucleotidase (CD73), all of which form part of calcium-phosphate nucleation. VICs, macrophages, and VSMC have been shown to release MVs, further promoting biomineralization 661-664. Studies suggest that mineralization begins within MVs, with Ca^+2^ and PO_4_^3-^ influx triggering hydroxyapatite formation upon their attachment to the ECM. However, alternative mechanisms propose that mineralization originates intracellularly before propagating to MVs and ECM fibers 665.

As the disease progresses, valve thickening and fibrosis result from excessive ECM deposition, particularly collagen accumulation, leading to tissue hardening and scarring 666. TGF-β has been identified as a major regulator of this fibrotic phase, promoting ECM synthesis and deposition through canonical Smad signaling. Elevated TGF-β levels have been detected in fibrotic organs, including stenotic aortic valves, where it drives collagen overproduction 667. However, this fibrotic phase is followed by increased ECM turnover, leading to a decline in collagen I and III content and further ECM remodeling. These events are tightly linked to VIC osteogenic differentiation, reinforcing the pathological transformation of the valve 668. Nevertheless, hypoxia is involved in ECM remodeling and calcification. Neovascularization in aortic valves restores oxygen supply but also enhances VIC activation and ECM remodeling, contributing to the formation of calcific nodules 669. A high expression of hypoxia-inducible factor-1 alpha (HIF-1α) has been detected in calcified nodules, driving ECM degradation through pathways involving NF-κB, MMP-2, MMP-9, and neutrophil gelatinase-associated lipocalin (NGAL) 670. NGAL is known for its ability to enhance MMP-9 activity, accelerate ECM breakdown, and further destabilize the valve microenvironment 671. In hypoxic aortic valves, the MMP-9-NGAL complex is upregulated, facilitating progressive tissue degradation. Moreover, the presence of ectopic elastic fibers in the fibrosa suggests disruptions in elastin homeostasis, indicating that hypoxia-driven ECM remodeling affects both collagen and elastin metabolism, further contributing to CAVD pathogenesis 672.

#### Osteoarthritis

Osteoarthritis (OA) is a chronic, degenerative joint disease and a leading cause of disability worldwide 673. It is characterized by progressive articular cartilage degeneration, subchondral bone remodeling, osteophyte formation, synovial inflammation, and pathological calcification of joint structures, all of which contribute to joint dysfunction. Despite its significant impact, there are currently no effective treatments to halt or reverse OA progression due to its complex biology 674. While calcification is a normal physiological process in bone formation, its abnormal deposition in cartilage and soft tissues is a key pathological feature of OA 675. The accumulation of calcium in the ECM exacerbates joint degradation, with calcium deposition in the cartilage matrix being closely linked to disease severity. Inhibiting ECM calcification has been shown to slow OA progression, enhancing the importance of understanding the molecular mechanisms behind cartilage calcification as a potential therapeutic target 676.

Krüppel-like factor 10 (Klf10) is a transcription factor regulated by the TGF-β/Smad signaling pathway and has an important role in bone biology and cartilage homeostasis 677. It contains a C_2_H_2_ zinc finger DNA-binding domain, enabling it to interact with promoter regions such as the CACCC element and GC box 678. Overexpression of Klf10 in chondrocytes inhibits cell proliferation and migration, while its deletion has been shown to prevent chondrocyte hypertrophy, a key process in cartilage calcification and longitudinal bone growth. However, the role of Klf10 in pathological cartilage calcification remains poorly understood 679.

Recent findings suggest that Frizzled9 (Fzd9), a G-protein-coupled receptor and a member of the Wnt signaling pathway, may be a downstream target of Klf10 680. Fzd9 is expressed in various tissues, including the brain, testes, skeletal muscle, and kidneys, and is known to regulate bone formation during fracture healing 681. Unlike canonical Wnt signaling, Fzd9 functions through a β-catenin-independent mechanism to positively regulate bone remodeling 682. Studies have shown that Klf10 binds to the Fzd9 promoter, modulating its expression and influencing calcium ion entry into chondrocytes, which is critical for ECM mineralization 683. In OA, calcium phosphate (BCP) and calcium pyrophosphate dihydrate (CPPD) crystals are the primary components of pathological calcification. These crystals are not only involved in ECM mineralization but also strongly associated with chondrocyte senescence, with CPPD having a more pronounced effect 684. Experimental data indicate that Klf10-induced chondrocyte senescence is mediated through abnormal calcium deposition and that knockdown of Klf10 reduces ECM calcification in mouse primary chondrocytes 685. Furthermore, restoring ECM calcification using BCP or CCPD crystals accelerates chondrocyte aging, reinforcing the link between calcification and cellular senescence 686. *In vivo*, studies using a destabilization of the medial meniscus (DMM) model of OA In mice demonstrated that Klf10 knockdown mitigated cartilage ECM calcification and slowed cartilage degeneration. These findings suggest that targeting Klf10 and its downstream pathways may provide a novel therapeutic approach for preventing cartilage mineralization and chondrocyte species, thereby attenuating OA progression 686.

#### Calcific aortic valve

Calcific aortic valve (AoV) disease is a major clinical challenge with poorly understood regulatory mechanisms 687. Although enhanced cell-cell adhesion is known to contribute to cellular aggregation, its role in calcific lesion formation remains unclear 688. Cadherin-11 (Cad-11) has been implicated in lesion formation *in vitro*, but its function in adult valve homeostasis and disease progression has not been established 689. Using a novel double-transgenic Nfatc1Cre; R26-Cad11Tg/Tg mouse model, researchers induced conditional overexpression of Cad-11 in heart valves 690. This led to hemodynamically significant aortic stenosis and the development of calcific lesions within the AoV leaflets. Cad-11 upregulation activated RhoA and Sox9 in VICs, promoting pathogenic ECM remodeling and calcification 691. *In vitro*, mimicking *in vivo* lesions, molecular analyses confirmed the upregulation of osteoblastic and myofibroblastic markers, reinforcing the role of Cad-11 in osteogenic differentiation 692. Further experiments demonstrated that inhibition of Rho-associated protein kinase (ROCK) significantly reduced calcific nodule formation, confirming that Cad-11-driven calcification is mediated through the RhoA/ROCK signaling pathway 693. These findings identify Cad-11 as a role regulator of AoV calcification and suggest that targeting the Cad-11/RhoA/ROCK axis could serve as a therapeutic strategy to prevent or mitigate aortic valve calcification 692.

Inflammation in the cardiovascular system induces significant alterations in the ECM, similar to its effects in other organ systems 694. During this process, certain matrix proteins experience increased expression and secretion, while the pre-existing ECM undergoes degradation and remodeling through the action of MMPs 695. In vascular tissues, inflammation is initiated when lipoprotein particles accumulate within the subendothelial matrix, where they undergo oxidative modifications, triggering an immune response and fueling a chronic inflammatory state 696. Pro-inflammatory stimuli, including cytokines and lipid oxidation products, have been shown to promote the osteoblastic differentiation of vascular cells, contributing to atherosclerosis and valvular cell calcification in CAVD 697. Molecular imaging studies further support this link, revealing that inflammation precedes matrix calcification in the vasculature and that inflammatory mediators are closely associated with intimal arterial calcification 698. Recent research has enhanced the role of smooth muscle cell (SMC)-derived exosomes in vascular calcification 699. Some studies demonstrated that exposure to TNF-α, a key inflammatory cytokine, enhances the production of exosomes by SMCs, which may contribute to ECM mineralization 700. Additionally, cell apoptosis often accompanies an inflammatory response, and accumulating evidence suggests that apoptotic cell death further accelerates vascular calcification, reinforcing the connection between chronic inflammation and pathological ECM remodeling 701.

#### Placenta calcification

Also, in the placenta, the ECM has a pivotal role in its development and function, regulating processes such as angiogenesis, trophoblast invasion, and tissue remodeling 702. In pathological pregnancies, such as those complicated by chronic venous disease (CVD) or preeclampsia (PE), ECM homeostasis is disrupted, leading to structural and functional alterations in placental tissue 703. One of the most notable changes is placental villous calcification, which has been associated with abnormal gene expression and signaling pathways involved in osteogenesis, inflammation, and vascular remodeling 704.

Among the key transcription factors involved in calcification, Runt-related transcription factor 2 (RUNX2) has a crucial role in bone and vascular calcification by regulating the expression of osteogenic genes, including type I collagen, osteopontin (OSP), osteocalcin (OSC), and bone sialoprotein 704. Interestingly, RUNX2, OSP, and OSC have been detected in vascular and placental calcifications, suggesting that pathological mineralization processes in the placenta may share molecular mechanisms with bone tissue 704. Also, the Wnt/β-catenin signaling pathway, which is essential for embryonic development and tissue homeostasis, is activated in placental calcification and may contribute to dysregulated mineral deposition in CVD and PE 705.

Another key regulator of placental pathology is pigment epithelium-derived factor (PDEF), which has been found at elevated levels in the placenta with venous insufficiency and is implicated in vascular dysfunction and calcification 706. Transcription factors such as MSX2/HOX8 and SOX9, which are involved in osteogenesis and chondrogenesis, have also been identified as potential contributors to placental ECM remodeling 707. These molecular pathways, however, remain poorly understood in the context of placental vascular disease, necessitating further investigation into their role in placental function and development.

Beyond calcification, CVD during pregnancy has been linked to increased placental apoptosis, extracellular matrix remodeling, and cellular hypoxia, similar to the mechanism observed in PE and intrauterine growth restriction (IUGR) 708. Placental angiogenesis is particularly affected, with alterations in VEGF signaling having a pivotal role. Increased expression of VEGF and its receptor (VEGFR-1/Flt-1 and VEGFR-2/KDR) has been reported in CVD placentas, enhancing their involvement in disease progression 709. Notably, VEGF regulates the expression of soluble Flt-1 (sFlt-1), a decoy receptor for VEGF and placental growth factor (PIGF), which is highly upregulated in PE and other vascular pregnancy complications 710. Dysregulation of the sFlt-1/PIGF ratio has been proposed as a biomarker for PE and IUGR, as lower PIGF levels correlate with adverse pregnancy outcomes and increased risk of urgent delivery 711.

Another important factor in ECM remodeling and placental pathology is the insulin-like growth factor (IGF) system 712. IGF-1 and its binding proteins (IGFBPs) are involved in fetal growth and nutrient transport across the placenta, and their dysregulation has been linked to IUGR 713. In animal models, IGF-1 supplementation has been proposed as a potential therapy to restore placental function in growth-restricted pregnancies 714. Furthermore, PAPP-A (pregnancy-associated plasma protein-A), a regulator of IGF bioavailability, is overexpressed in the placenta affected by CVD, with ECM alterations contributing to its dysregulated expression. Given that PAPP-A interacts with calcium ions and is involved in proteolysis, its upregulation may further exacerbate placental calcification, reinforcing the pathogenic role of ECM remodeling in vascular pregnancy disorders 715.

The importance of ECM integrity in placental function extends beyond vascular regulation, as it also has a role in perinatal neurodevelopment 716. Elevated levels of reelin and ECM glycoprotein have been associated with cerebral blood redistribution, suggesting a link between placental ECM changes and fetal neurological outcomes 717. Additionally, dysregulated ECM composition, including collagen fiber alterations and increased villous calcification, has been consistently observed in the CVD-affected placenta 718. These findings underscore the systemic impact of vascular pregnancy diseases, not only on the mother but also on fetal development.

#### Impact of MMPs in calcification

The ECM forms a loose, hydrated network rich in glycoproteins in healthy tissue, maintaining flexibility and structural integrity. However, as the valvular disease progresses, the ECM becomes increasingly dense and fibrotic due to excessive collagen deposition and remodeling 719. *In vitro* studies have shown that calcifying vascular cells (CVCs) produce an ECM with elevated levels of collagen I and fibronectin but reduced collagen IV, compared to normal VSMC 720. This shift in matrix composition creates a stiffer, more adhesive microenvironment, which may further promote mineralization and calcification. In advanced atherosclerosis, the intimal layer thickens and exhibits increased expression of matrix proteins, including thrombospondin, tenascin, osteopontin, osteocalcin, and dentin matrix acidic phosphoprotein 1 (DMP-1) 721. In the aortic valve, VICs respond to injury by upregulating fibronectin production, further contributing to pathological ECM remodeling. As valvular disease advances, expression of key osteogenic markers, such as osteopontin and osteocalcin, continues to rise, alongside an increase in MMPs (MMP-1, MMP-3, and MMP-9) and their inhibitors, which regulate ECM turnover and degradation. In addition to structural alterations, inflammation-driven matrix changes impact the vascular lipid microenvironment 722. The elevated fibronectin content in diseased tissue enhances lipoprotein retention, likely through its heparin-binding domain, while proteoglycans also facilitate lipoprotein binding. These interactions contribute to lipid accumulation, further exacerbating vascular dysfunction and disease progression 723.

On the other hand, the MMP family is involved in early bone and dentin formation to fracture healing, and pathological calcification 724. In osteoblasts and osteocytes, MMP-13 expression has been associated with osteogenic differentiation, and studies in MMP-2-deficient mice have demonstrated progressive loss of bone mineral density and impaired calcification, reinforcing the importance of ECM degradation in matrix mineralization 725. Similarly, matrix vesicles, which initiate calcification, contain MMP-2 and MMP-9, further improving the contribution of proteolytic enzymes in remodeling the ECM and promoting calcium phosphate deposition. Beyond MMPs, another family of extracellular proteases, the ADAMTS family, has been implicated in ECM turnover during bone development. Enzymes such as ADAMTS-1, ADAMTS-4, and ADAMTS-5 degrade proteoglycans like versican, which accumulate in the ECM and may regulate mineralization 726. Evidence suggests that ADAMTSs and MMPs function cooperatively, accelerating ECM breakdown and facilitating matrix calcification during bone formation 727. These findings underscore the complex interplay between ECM degradation, mineralization, and proteolytic enzyme activity 728. While ECM proteins serve as essential regulators of bone and dentin calcification, further studies are needed to fully elucidate the balance between mineralization inhibitors and the enzymes that degrade them 729. Understanding these regulatory mechanisms could provide novel insights into bone regeneration, dentin repair, and pathological calcification processes 730.

### Interaction of ECM and cancer

Traditional perspectives on cancer have evolved to recognize the ECM as a fundamental regulator of cell proliferation, migration, and apoptosis 731. The organization and composition of ECM components form a tissue-specific microenvironment that has a primary action in tumor progression 732. It is established that ECM is not merely a passive scaffold but undergoes continuous remodeling, actively influencing cell adhesion, migration, and signaling pathways 733. Even minor distribution in ECM homeostasis can significantly affect cancer cell behavior, making ECM remodeling a key determinant in tumor growth and metastasis 734. Its remodeling is a dynamic process governed by four primary mechanisms that collectively regulate tissue structure, biochemical signaling, and mechanical properties 735. These processes include ECM deposition, which alters the abundance and composition of ECM components, thereby influencing both biochemical and mechanical properties 736; post-translational chemical modifications, which affect the structural characteristics and biochemical functionality of the ECM 737; proteolytic degradation, which releases bioactive ECM fragments and ECM-bound factors, often facilitating processes such as cell migration by eliminating physical constraints 738; and force-mediated physical remodeling, which reorganizes ECM fibers, aligning them to create pathways for cellular migration 739. Tissue homeostasis relies on the precise regulation of ECM deposition, modification, degradation, and organization, as even small disturbances in these processes can disrupt the balance and lead to pathological consequences 740. ECM components serve as key ligands for various cell surface receptors, including integrins, Syndecans, and receptor tyrosine kinases, integrating ECM remodeling into broader cellular signaling networks 741. Given this intricate interplay, it is not surprising that cancer cells and tumor-associated stromal cells actively manipulate all four remodeling mechanics 735.

Collagen, the most abundant ECM protein, is central to maintaining tissue integrity and function 742. Changes in collagen deposition, degradation, and crosslinking can lead to a loss of ECM homeostasis, contributing to tumor progression 743. Tumor-associated ECM remodeling involves increased secretion of fibronectin and collagens I, III, and IV, enhancing the dynamic interplay between tumor cell and their microenvironment 744. Excessive ECM protein deposition disrupts cell-cell adhesion and polarity, amplifying growth factor signaling and fomenting tumor progression 745. However, the precise role of collagen is complex (increased collagen crosslinking promotes integrin signaling and tumor progression) 746. Yet, the depletion of fibrillar collagens can also drive malignancy by altering biomechanical forces within the tumor niche 746. Collage also exerts its influence through DDR1 and DDR2, which are implicated in cancer progression. DDR1 is essential for collective cell migration, while DDR2 747. has been identified in invasive breast tumors, where it stabilizes SNAIL1, promoting epithelial-mesenchymal transition (EMT) and metastasis 748. The extracellular collagen network, through DDR signaling, thus modifying cell-intrinsic properties, reinforces tumor invasiveness 748.

The ECM serves as both a barrier and a promoter of tumor progression, depending on its composition and structural modifications 735. ECM gene signatures have also been used to stratify breast cancer subtypes, with tumors exhibiting high protease inhibitor expression correlating with better prognosis 749. In contrast, those with high MMP expression are linked to poor survival and increased recurrence risk. MMPs have been linked to cancer progression, with initial research suggesting that MMP-mediated ECM degradation facilitates tumor invasion and metastasis 750. Early studies demonstrated that MMP inhibition reduced tumor invasiveness in animal models, leading to clinical trials to block MMP activity 751. Beyond degrading physical barriers, MMPs influence multiple signaling pathways, modulating both normal physiological processes and disease progression 752. Their role extends beyond ECM remodeling to impact cellular communication, immune response, and tumor microenvironment regulation 753. Also, ADAM and ADAMTS protease families, which share structural similarities with MMPs, have been implicated in tumor progression and are targeted by broad-spectrum inhibitors designed to suppress metzincin protease activity 754. While MMPs are predominantly associated with cancer promotion, emerging evidence suggests that some MMPs and other extracellular proteases may exhibit tumor-suppressing effects under certain conditions 755. Their diverse biological functions extend beyond cancer, influencing tissue homeostasis and remodeling in both healthy and diseased states 756. These findings underscore the dual nature of MMP activity, enhancing the need for a more nuanced therapeutic approach when targeting these enzymes in cancer treatment.

The lethality of most cancers is due to the dissemination of metastatic tumor cells and the growth of secondary tumors in distant organs 757. Metastatic initiation requires tumor cells to invade peripheral tissues, enter the blood or lymphatic system (intravasation), and establish secondary tumors in receptive environments known as premetastatic niches 758. MMPs facilitate this process by degrading ECM barriers, such as the endothelial basement membrane, creating pathological metastasis-prone vasculature 759. MMP-1 expression has been particularly implicated in classifying atypical ductal hyperplasia into being versus pre-malignant lesions, reinforcing the ECM role in cancer progression and risk assessment 760. Also, MMP-1 activates proteinase-activated receptor-1 (PAR-1), a G-protein-coupled receptor involved in thrombosis and inflammation 761. This activation promotes cancer cell migration and invasion, particularly in breast, colon, and lung cancers, underscoring the significance of stroma-derived proteinases in tumor progression 762. Bone metastases, a frequent complication in cancers such as breast and prostate cancer, are also mediated by MMPs 763. MMP-7, expressed at the tumor-bone interface, facilitates osteolysis by cleaving receptor activator of nuclear factor κB ligand (RANKL), leading to osteoclast activation and enhanced bone degradation 764,765. Similarly, MMP-1 and ADAMTS-1 activate EGF-like ligands, further stimulating the RANKL pathway and promoting bone metastasis. Beyond ECM degradation, MMPs contribute to tumor progression by reshaping the tumor microenvironment and influencing immune cell interactions 766. Some studies using high-resolution multimodal microscopy confirm that MMP-14-driven pericellular proteolysis enables single-cell and collective-cell migration, reinforcing the importance of ECM remodeling in tumor invasion 767. Interestingly, metastatic cells can switch from protease-dependent to protease-independent amoeboid migration, allowing them to navigate the ECM without proteolytic degradation 768. Additionally, tumor-associated macrophages (TAMs) are key in tumor motility and intravasation 769. Macrophage-derived MMP-2 and MMP-9 facilitate immune cell migration and may contribute to tumor cells in circulation by degrading the endothelial basement membrane 769.

**Figure 7:**
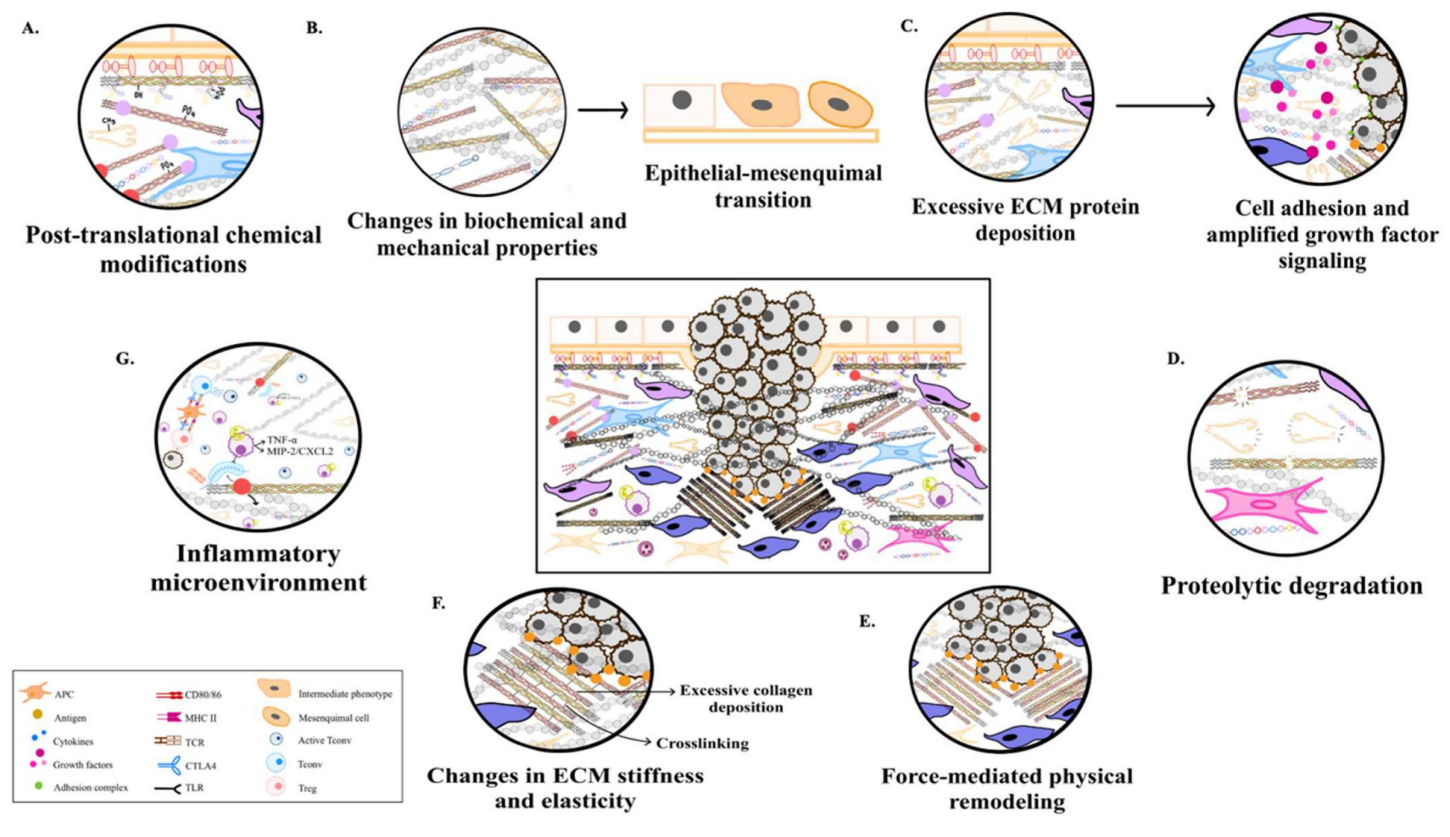
The extracellular matrix (ECM) undergoes a series of processes that favor the creation of a tissue-specific microenvironment that promotes tumor progression. These modifications include changes in post-translational chemical modifications (**panel a**), which lead to changes in biochemical and mechanical properties, promoting epithelial-mesenchymal transition (EMT) (**panel b**). Excessive ECM protein deposition also contributes to these changes. Furthermore, it leads to alterations in cell adhesion and amplification of growth factor signaling (**panel c**). The ECM is also subject to proteolytic degradation (**panel d**). Together with force-mediated physical remodeling (**panel e**), both lead to ECM fiber remodeling, which, along with collagen deposition and crosslinking, contributes to changes in ECM stiffness and elasticity (**panel f**). The active modification of the extracellular matrix by stromal and tumor cells generates an inflammatory microenvironment that facilitates tumor invasion. Matrix proteases release bioactive fragments, such as the proteoglycan biglycan, which stimulate Toll-like receptors in macrophages by promoting the release of TNF-α and MIP-2/CXCL2. In addition, antigen-presenting cells (APCs) stimulate the activation of T lymphocytes by presenting tumor antigens (**panel g**). This sustained inflammation creates a favorable environment for metastasis. All these processes together promote cancer progression and increase its invasiveness, contributing to the transformation of the primary tumor into a distal secondary tumor.

ECM remodeling extends beyond primary tumor sites, influencing metastatic colonization (**Figure 7**). Changes in ECM stiffness and elasticity, governed by collagen organization, crosslinking, and chemical modifications, allow tumor cells to interact with and adapt to their environment 770. Increased ECM stiffness, driven by excessive collagen deposition and crosslinking, disrupts normal tissue architecture and promotes tumor invasion 771. This process is largely mediated by LOX and LOX-like (LOXL) enzymes, which are frequently overexpressed in primary and metastatic tumors 771. High LOX expression correlates with poor survival outcomes, particularly in breast cancer, where LOX-induced collagen crosslinking activates β1 integrin clustering, PI3K signaling, and focal adhesion formation, driving tumor progression 772. Inhibiting LOX has been shown to reduce fibrosis and delay tumor development, demonstrating its potential as a therapeutic target 773. MicroRNAs (miRNAs) are emerging as potent regulators of ECM remodeling. The miR-29 family, for example, modulates genes involved in ECM dynamics, including collagen chains, LOX, and MMPS, such as MMP2 and MMP9 774. In breast cancer, miR-29b overexpression alters the tumor microenvironment and suppresses metastasis 775. These findings suggest that non-coding RNAs, including long non-coding RNAs, may regulate the chromatin state and transcription of ECM genes, presenting new avenues for research into tumor-associated ECM regulation 776.

**Figure 8:**
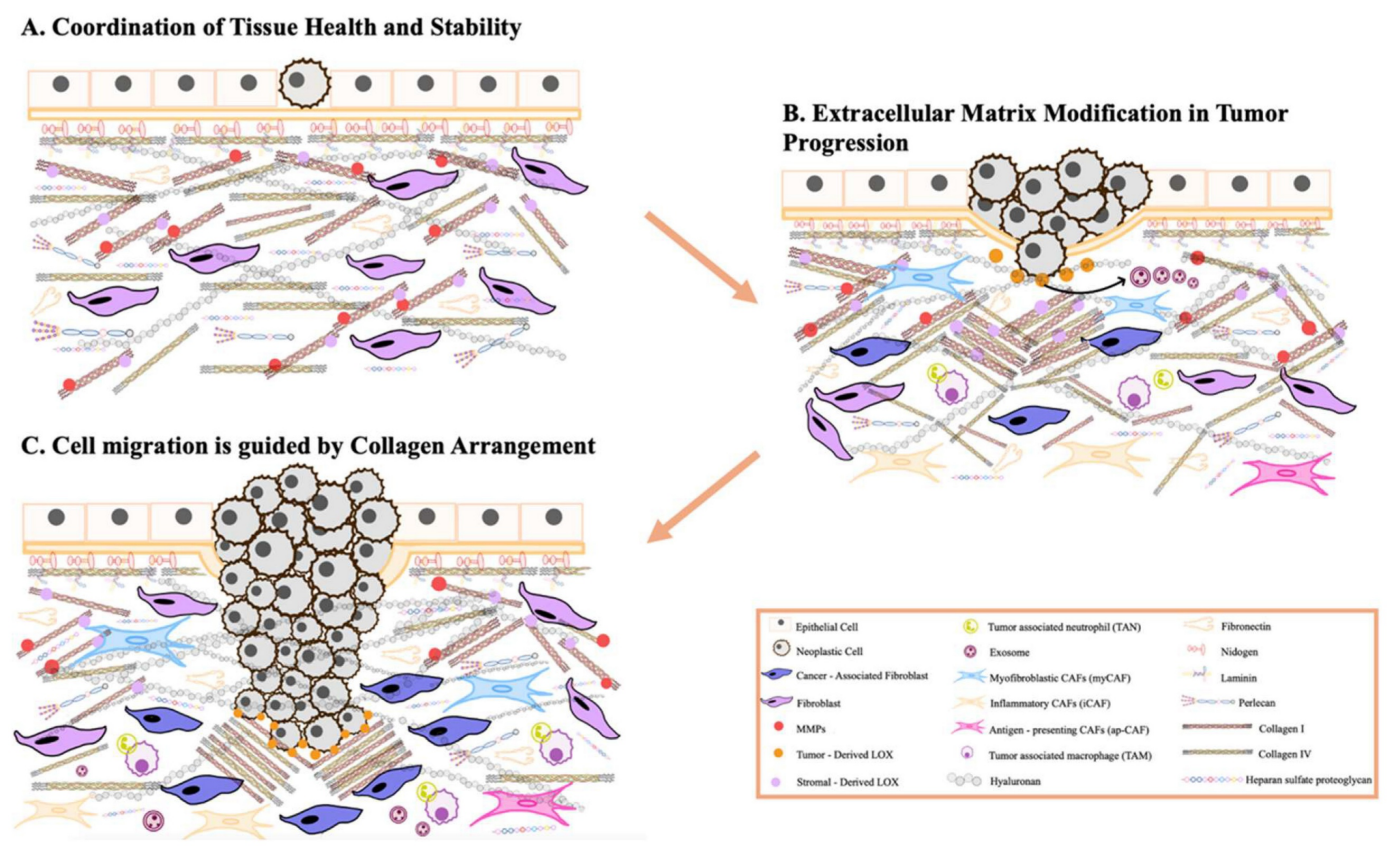
** A.** Coordination of Tissue Health and Stability: Initial tumor lesion showing the basement membrane, composed of laminin, collagen IV, and nidogen, which separates epithelial cells from the extracellular matrix. The extracellular matrix contains fibroblasts, collagen I/III, fibronectin, hyaluronan, and other proteoglycans. **B.** Extracellular Matrix Modification in Tumor Progression: Neoplastic cells proliferate rapidly, releasing extracellular vesicles into the extracellular matrix and inducing mechanical strain on the basement membrane, causing it to bulge. Matrix metalloproteinases (MMPs) contribute to tumor progression by reshaping the tumor microenvironment, degrading ECM components locally, and influencing immune cell interactions. Immune cells like tumor-associated macrophages (TAMs) and tumor-associated neutrophils (TANs) also contribute to ECM remodeling by producing cytokines and growth factors that activate cancer-associated fibroblasts (CAFs). CAFs deposit collagen I, which is aligned by stromal-derived lysyl oxidase (LOX), while hyaluronan, a glycosaminoglycan involved in tissue hydration, absorbs water, increasing interstitial fluid pressure and promoting ECM swelling. **C.** Cell migration is guided by Collagen Arrangement: Neoplastic cells breach the basement membrane and migrate along aligned collagen fibers. Tumor-derived LOX further stiffens the ECM through collagen crosslinking. The production of cytokines and growth factors by immune cells is increased, and, therefore, the activation of CAFs is also enhanced, intensifying ECM remodeling and facilitating tumor invasion into surrounding tissues.

On the other hand, the tumor microenvironment (TME) (**Figure 8**) has a dynamic action, undergoing continuous ECM remodeling that influences immune evasion, tumor progression, and therapy resistance 777. Tumor and stromal cells actively modify the ECM, generating an inflammatory microenvironment that facilitates cancer invasion and metastasis 778.

ECM components can act as danger-associated molecular patterns (DAMPs), activating pattern recognition receptors (PPRs) on immune cells and triggering pro-inflammatory responses 779. ECM-degrading proteases liberate bioactive ECM fragments, including low-molecular-mass hyaluronan (LMM-HA) and biglycan, alongside matrix-bound cytokines and growth factors, fueling chronic inflammation 780. For instance, biglycan activates Toll-like receptors (TLR4 and TLR2) on macrophages, promoting TNF-α and MIP-2/CXCL2 expression, which sustains tumor-associated inflammation 781.

Beyond their role in modulating immune responses, ECM components also regulate immune cell infiltration and tumor immunity 782. The ECM proteoglycan versican, when cleaved by ADAMTS proteases, generates versikine, a bioactive fragment that enhances CD8^+^ T-cell infiltration in colorectal cancer and multiple myeloma 783. Versikine increases Interferon Regulatory Factor 8 (IRF8) expression in macrophages, promoting the generation of CD103^+^ CD11c^+^ MHCII^hi^ conventional dendritic cells (cDCs), which play a key role in anti-tumor immunity 784. However, the TAMs that infiltrate tumors contribute to ECM remodeling in ways that both support and suppress tumor progression 785. M2-like TAMs facilitate proteolytic clearance of interstitial collagen through upregulated MMP expression, including pro-angiogenic TIMP-1-free MMP-9, which, along with neutrophils, increases ECM degradation and tumor invasion 786.

Additionally, TAMs contribute to ECM deposition, upregulating the synthesis and assembly of collagens I, VI, and XIV, leading to enhanced matrix cross-linking and stiffening, which favors tumor cell invasion 787. Mechanical stress is another factor influencing immune evasion and therapy resistance in the TME, which arises from solid stress, fluid shear stress, and increased intratumoral pressure 788. These physical forces drive cancer mechanobiology, altering tumor architecture and triggering immune defense mechanisms, including EMT and autophagy, suggesting that targeting these mechanical forces could enhance immunotherapy efficacy.

The adaptive immune system, particularly cytotoxic T cells, is important in immune surveillance, identifying and eliminating tumor cells by recognizing mutated or foreign antigens 789. However, the ECM exerts dual effects on the tumor immune response, acting as both a facilitator and a barrier to T-cell infiltration and activation 790.

On the one hand, the ECM supports T-cell migration by providing structural pathways (migratory highways) that guide immune cells toward tumors. Also, ECM degradation products, generated through MMP-mediated cleavage, can act as chemoattractants, enhancing immune cell infiltration 791. This has been demonstrated in inflamed lung tissue, where MMP12 and elastase-driven elastin digestion facilitates monocyte migration 792.

Conversely, ECM stiffening can suppress T-cell function, impairing antigen representation by antigen-present cells (APCs) and disrupting T-cell activation 793. The ligation of type I collagen to LAIR receptors can directly inhibit T-cell proliferation, while increasing ECM stiffness may interfere with CD3/CD28 signaling, reducing IL-2 production (a cytokine essential for T-cell expression and Th1 differentiation) 794. As a result, a tumor-associated stiffened ECM may compromise anti-tumor immunity, contributing to immune evasion and cancer progression 795.

Chronic inflammation is a defining feature of tumors and significantly increases the risk of malignant transformation 796. Notably, persistently inflamed tissue often exhibits fibrotic remodeling, characterized by excessive collagen and fibronectin deposition, which not only alters tissue architecture but also influences immune cell recruitment and activation 797.

The ECM has a pivotal role in regulating immune infiltration. For instance, neutrophil recruitment is severely impaired in the absence of α6β1 integrin, a receptor for laminin 798. At the same time, macrophage infiltration into atherosclerotic plaques requires DDR1 protein, which inhibits macrophage infiltration, enhancing the regulation of ECM complexity in immune cell migration 799.

Beyond recruitment, ECM composition influences macrophage activation and polarization. A collagen-rich ECM supports macrophage proliferation and promotes M2 polarization, which is associated with pro-tumorigenic activity 800. In contrast, a fibronectin-rich ECM enhances M1 macrophage polarization, which supports anti-tumor immune responses 801,802. In tumor microenvironments, ECM stiffening, particularly through type I collagen accumulation, favors an immunosuppressive M2 phenotype, potentially by diminishing TNF-α expression in response to inflammatory stimuli like LPS 803.

For instance, the immune system can prevent tumor formation, yet age-related chronic inflammation (inflammaging) creates an environment that supports tumor initiation and progression 804. This persistent inflammatory state disrupts acute immune responses, promotes tissue degradation, and is linked to multiple age-related malignancies 805. Cellular senescence contributes significantly to inflammaging through the SASP, which maintains a low-grade inflammatory response 806. Other factors, such as gut microbiota alterations, obesity, and ECM remodeling, further drive this process by increasing systemic levels of pro-inflammatory cytokines (IL-1, IL-6, TNF) 807.

A key link between inflammaging and cancer is the recruitment of myeloid-derived suppressor cells (MDSCs), which impair T-cell function through the secretion of ARG1, TGF-β, and ROS 808. In a melanoma mouse model, upregulated inflaming mediators (IL-1β, GM-CSF, and IFNγ) promoted MDSCS infiltration, accelerating tumor progression and metastasis. Pharmacological inhibition of MDSCS immunosuppressive activity reversed these effects, restoring T-cell function and reducing tumor growth 809.

Similarly, regulatory T cells (Tregs), which suppress effector T-cell responses, are highly enriched in chronic inflammatory environments 810. In an allergic contact dermatitis (ACD) model, the cytokine IL-33 forms part of the shifting of acute inflammation into a chronic, tumor-promoting state 811. IL-33-deficient mice were protected against carcinogen-induced skin cancer, suggesting that the IL-33-Treg axis contributes to chronic inflammation-driven tumorigenesis, including colitis-associated colorectal cancer 812.

In breast cancer, IL-6, a major inflammaging cytokine, has been identified as a driver of tumor progression, with high serum levels correlating with poor prognosis 813. Also, several inflammaging-associated microRNAs (inframammary), such as miR-19, miR-21, miR-126, and miR-146a, have been linked to cancer progression, partially through their regulation of inflammatory cytokines and immune signaling pathways 814-816.

Finally, it is observed that EVs are a crucial component of the SASP, facilitating intercellular communication by transporting proteins, mRNAs, and DNA between cells 817. Notably, EVs can also accumulate within the ECM, interacting with matrix components and influencing ECM remodeling. The cargo within senescence-associated EVs is influenced by age, sex, and environmental exposures, underscoring their role in age-related diseases 818. EVs have been implicated in idiopathic pulmonary fibrosis (IPF), a progressive fibrotic lung disease. Elevated exosomal miR-21, a key driver of inflammaging, has been detected in circulating EVs from IPF 819. Interesting that senescent alveolar epithelial cells (AECs) play a pivotal role in IPF progression, with miR-21 upregulation observed in both fibrotic lung tissues and experimental models Furthermore, EVs from IPF lung fibroblasts contain miR-23 b-3p and miR-494-3p, which inhibit SIRT3, a regulator of mitochondrial homeostasis, and their expression correlated with disease severity 820.

Senescent fibroblasts also increase EV secretion, amplifying paracrine signaling that accelerates mitochondrial damage and epithelial cell senescence, thereby perpetuating lung fibrosis 821. However, EV effects are highly context-dependent 822. For instance, EVs derived from inflammatory and myofibroblast cell death suggest a potential antifibrotic role in certain conditions 823.

**Figure 9:**
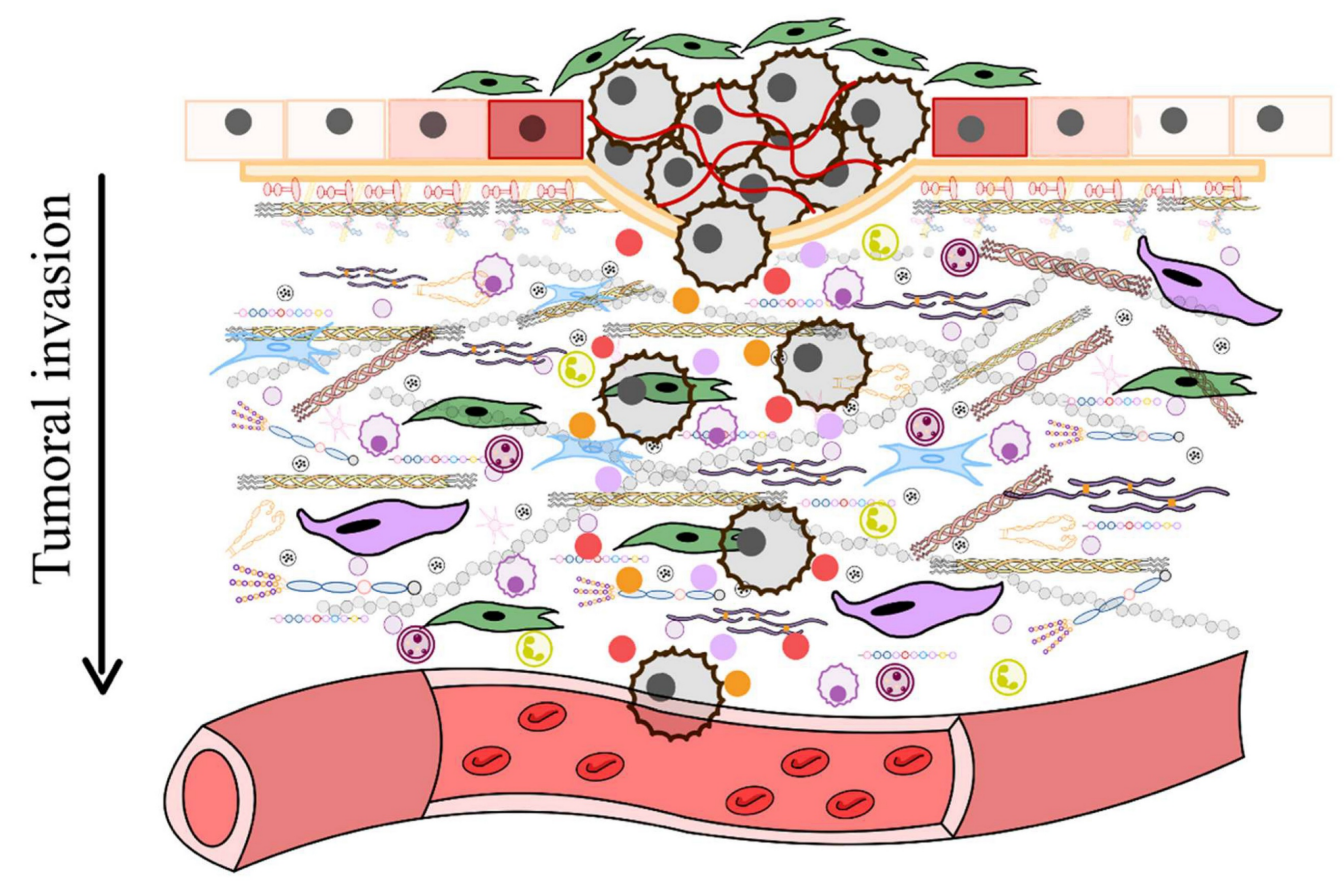
Representation of how a senescent extracellular matrix (ECM) promotes tumor invasion and the metastatic process in cancer. The presence of senescence-associated fibroblasts around tumor cells is observed, which, together with the molecules released by the senescence-associated secretory phenotype (SASP), facilitates the dissemination of these tumor cells.

In essence, senescent cells not only lose their proliferative capacity but actively reshape the ECM in ways that create a fertile ground for cancer development and metastasis, transforming the surrounding tissue into a landscape that favors tumor cells' invasion and survival **(Figure 9)**.

## Applications of ECM in different pathologies

The ECM is a highly dynamic, three-dimensional network that plays a crucial role in cell morphology, function, and tissue integrity. Under normal physiological conditions, ECM remodeling is tightly regulated, ensuring proper cellular behavior 824. However, in pathological states such as cancer, ECM homeostasis is disrupted, leading to uncontrolled remodeling that drives disease initiation and progression 825.

The ECM comprises numerous interacting molecules that communicate with neighboring cells via cell surface receptors, forming complex interaction networks 826. Understanding these interactions is necessary to identify novel disease biomarkers and develop personalized therapeutic interventions. Recent advancements in big data analytics have facilitated the creation of online databases that enable stochastic evaluations of ECM interactions, helping researchers filter relevant molecular pathways for their specific studies 827.

Some studies have a data-driven approach that addresses existing limitations in ECM research, broadens their understanding of ECM-mediated cellular functions, and has the potential to enhance targeted therapy developments 828.

Ongoing studies continue to highlight the importance of ECM regulation in disease pathology, emphasizing the need for pharmacological strategies to modulate ECM interactions and restore tissue homeostasis.

The extracellular matrix is significantly altered in various diseases, including atherosclerosis, chronic inflammation, and cancer 829. In recent years, attention has shifted towards targeting the tumor microenvironment, particularly ECM components, as they play a crucial role in tumor progression, drug resistance, and immune evasion 829. Unlike healthy tissues, cancer-associated ECM undergoes abnormal remodeling, creating physical and biochemical barriers that hinder drug delivery and therapeutic efficacy 830.

Targeting ECM components has emerged as a promising therapeutic strategy. Several clinical trials are investigating approaches such as chimeric antigen receptor (CAR) T-cell therapies against glypican-3 in liver cancer or heparanase inhibitors for multiple myeloma. Also, PGs, MMPs, and CD44/HA signaling pathways have been explored as potential drug targets due to their role in tumor growth and ECM interactions 831. One major challenge in cancer therapy is inefficient drug transport caused by abnormal vasculature, increased interstitial fluid pressure (IFP), and excessive ECM deposition 832. Tumor blood vessels are often disorganized and leaky, creating hypoxic conditions that promote tumor survival 833. Strategies like vascular normalization through VEGF and PDGF inhibitors can improve blood flow and drug distribution 834. Additionally, collagen-degrading enzymes (MMPs and collagenase) and LOX inhibitors have been shown to reduce ECM stiffness, enhancing drug penetration 835.

Biomaterials are also being investigated as drug carriers to overcome ECM-related resistance. Natural and synthetic matrices, including collagen, gelatin, fibrin, and alginate, have been engineered to improve drug delivery and tissue regeneration 836. The incorporation of ECM-derived peptides into biomaterials has demonstrated synergistic effects, promoting vascularization, wound healing, and bone regeneration 837. By interacting ECM-modulating therapies with existing cancer treatments, researchers aim to enhance drug efficacy, reduce metastasis, and improve patient outcomes 838.

Decellularized ECM materials have become versatile in tissue engineering, taking forms such as patches, powders, and injectable hydrogels 839. With additional processing, these ECM hydrogels offer minimally invasive delivery options for regenerative therapies 840-843. Derived from various tissue sources, ECM hydrogels have been applied in models of ischemic injury, organ regeneration, and replacement 844. They promote cellular infiltration, particularly of progenitor cells and macrophages, stimulate neovascularization, and support favorable functional remodeling 845,846.

Although their mechanism of repair remains incompletely understood, ongoing research seeks to optimize the selection, processing, and modification of ECM hydrogels for specific regenerative applications 847. New technologies like 3D printing and advanced modification protocols are enhancing the complexity of these hydrogels, enabling them to better mimic the target tissue environment 848. While acellular ECM platforms are closest to clinical application, there is potential for combination therapies incorporating stem cells or growth factors, albeit with increased complexity and cost 849. Commercial products and standardized manufacturing practices are emerging, setting the stage for broader clinical translation of ECM hydrogel therapies in regenerative medicine 850.

It should be noted, however, that while these emerging interventions are promising, a deeper critique regarding their clinical translatability, limitations, and the available preclinical and clinical evidence would be valuable. More studies are needed to fully understand the ECM functions, remodeling, and impact on human health. **Table 9** summarizes all the therapeutic strategies discussed in this section.

### The therapeutic use of ECM in vascularization illness

Cardiac regeneration through stem cell therapy is a promising yet complex approach due to the heart´s limited intrinsic repair mechanism and irreversible remodeling after injury 851. Despite the early enthusiasm for resident cardiac stem cells (CSCs), their regenerative potential remains insufficient after myocardial infarction 852.

In myocardial infarction, for example, the use of stem cell therapy modifies the stiffness of the infarcted heart tissue by altering ECM composition, making the scar more compliant and cellular 853. It aims to restore heart function by repopulating cardiomyocytes, with various preclinical and clinical studies underway 854. Preconditioning strategies, such as heat shock, hypoxia, and chemical treatments, enhance cell survival and engraftment by inducing cytoprotective pathways and promoting angiogenesis. Hypoxia upregulates HIF-1 and CXCR4, improving graft integration and cardiac repair 855. However, clinical studies primarily focus on medical outcomes, with limited data on the long-term fate of transplanted cells and their effects on ECM remodeling 856.

After this, the strategies of stem cell therapy include cell transplantation (autologous/allogenic stem cells) and stimulation of endogenous progenitor cells via pharmacological, biological, or ECM-based methods 857. Different cell types, delivery methods, dosages, and administration timing contribute to variability in clinical outcomes, making direct comparisons difficult 858. While stem cell therapy is known to alter ECM composition, its role in modulating biomechanical properties and facilitating ECM remodeling remains unexplored 859. Given the unique composition of cardiac ECM, the ability of noncardiac-derived stem cells to restore heart-specific ECM proteins remains unclear, enhancing the need for further research 860.

For instance, cardiac tissue engineering faces key challenges, primarily cell source limitations and scaffold design 861. The myocardium´s stiffness, ranging from 20 kPa to higher values, is important in cell differentiation and functional maturation 862. Biodegradable scaffolds must endure the mechanical forces of the cardiac cycle while providing biochemical and structural cues for cell adhesion, migration, and integration 863. Decellularized ECM is a promising scaffold due to its MMP-sensitive peptides, spatial organization, and growth factors, facilitating vascularization and host cell proliferation 864. However, construction thickness is a major limitation, as oxygen diffusion constraints restrict the maximum viable thickness to 400 µm.

Several drugs, including omecamtiv mecarbil, spironolactone, and agrin, have shown potential in targeting the cardiac ECM and cytoskeleton for cardiovascular disease (CVD) treatment 865. Omecamtiv mecarbil, a myosin activator, enhances cardiac contractility and slightly reduces heart failure (HF) risk in patients with low ventricular ejection fraction (LVEF) 866. Agrin, an ECM proteoglycan, aids in neuromuscular junction formation and has demonstrated protective effects against myocardial infarction and dilated cardiomyopathy (DCM) 867. Spironolactone, a mineralocorticoid receptor antagonist, limits ECM turnover, reducing cardiac fibrosis and improving congestive HG outcomes. These examples enhance the reduction of cardiac fibrosis of ECM-targeting drugs in CVD management 868. However, concerns remain regarding unintended effects due to the fundamental roles of ECM and cytoskeletal proteins in cellular function 869. Despite this, ongoing research, supported by computational analyses, underscores the promise of ECM and cytoskeletal targeting as a novel approach to drug development 870. Vascularization strategies are being explored to overcome diffusion limits, improving tissue survival and function in engineered cardiac grafts. **Table 10 summarizes all the therapeutic agents used.**

### The strategies of ECM therapy in cancer development

The increasing global burden of cancer enhances the urgent need for precise treatment strategies and the discovery of novel therapeutic targets 871. Traditional therapeutic approaches, including biomarker-based treatments, immunotherapy, and chemotherapy, have significantly improved patient prognosis 872. Besides, aberrant gene expression and dysregulated signaling pathways in tumor cells drive pro-tumorigenic ECM remodeling, creating a hostile environment that impedes the infiltration of anti-tumor immune cells and therapeutic agents while simultaneously supporting cancer cell survival under stress induced by anti-cancer treatment 873,874. The ECM, as shaped by cancer cells, is closely linked to multiple forms of drug resistance.

In this sense, each tumor type is characterized by a unique gene signature, which is closely linked to specific ECM composition 875. Understanding these ECM-related gene signatures may provide valuable insights into a tumor´s sensitivity to therapy 876. Several studies have explored this connection; for instance, stromal-related gene signatures have been associated with prognosis in patients with large B-cell lymphoma undergoing chemotherapy 877.

Beyond genetic signatures, proteomic analyses of ECM proteins have also proven useful in distinguishing cancerous tissues from normal ones 878. More recently, a study revealed that matrix components released into the bloodstream following anti-PD-1 treatment in melanoma patients were predictive of treatment outcomes 879. Furthermore, TGF frequently controls ECM signature genes-β1 signaling, with COL11A1 showing upregulated expression during ovarian cancer progression 880. These findings underscore the critical role of ECM remodeling in cancer progression and resistance to therapy 881. Identifying pivotal genes involved in ECM regulation could be instrumental in designing more effective and personalized therapeutic strategies.

In cancer, immunotherapies harness the body´s immune system to combat cancer, offering promising treatment strategies. These approaches include immune checkpoint inhibitors, such as PD-1, PD-L1, and CTLA-4 blockers, which enhance anti-tumor activity within the TME 882. Another significant advancement is adoptive cell therapy (ACT), particularly chimeric antigen receptor (CAR) T-cell therapy 883. This method involves extracting T cells from patients, genetically engineering them to recognize tumor-specific antigens, and reintroducing them to target cancer cells more effectively 884. While some of these therapies have led to complete tumor remission, their effectiveness varies among patients and tumor types. Understanding the factors influencing treatment response is important for advancing immunotherapy strategies 885. The ECM plays a pivotal role in regulating immunotherapy efficacy. Studies have shown that ECM-related gene expression can prevent resistance to PD-1 blockade therapy 886. For example, in metastatic urothelial cancer, TGF-β-induced peritumoral collagen creates a barrier that prevents CD8^+^ T cells from reaching tumor cells, leading to poor treatment outcomes 886. In breast cancer, CAFs expressing ECM proteins and TGF-β signaling contribute to immune evasion by upregulating PD-1 and CTLA-4 in regulating T cells 887. Similarly, collagen-induced CD8^+^ T cell exhaustion in lung tumors has been linked to immune checkpoint therapy resistance 888.

Interestingly, ECM stiffness in tumors can also influence PD-L1 expression, with studies showing that depleting β3-integrin reduces PD-L1 levels and enhances PD-1 blockade efficacy 889. These findings suggest that combating ECM-targeting strategies with immunotherapy could improve treatment response 890. Indeed, targeting key ECM components such as TGF-β and PD-L1 has demonstrated increased ECM remodeling, enhanced CD8^+^ T cell infiltration, and a shift in macrophage activity towards an anti-tumor phenotype in colon and breast cancer models 891.

ECM remodeling strategies also facilitate the penetration of immunotherapeutic agents into tumors. In preclinical models of breast cancer and melanoma, the use of hyaluronidase to degrade HA enhances the efficacy of PD-L1 inhibitors and cancer vaccines by improving drug delivery 892. Similarly, oncolytic viruses engineered to express hyaluronidase generate low-molecular-weight HA, which activates NF-κB signaling in macrophages and promotes CD8^+^ T cell infiltration, ultimately enhancing PD-1 blockade efficacy in glioblastoma models 893.

CAR-T cell therapy has shown limited success in solid tumors, partly due to the EC acting as a physical barrier that restricts T cell infiltration 894. Expanding CAR-T cells *ex vivo* reduces their ECM remodeling capabilities, a limitation linked to low heparanase expression 895. Engineering CAR-T cells to express heparanase has been shown to improve their tumor-penetrating ability and enhance anti-tumor effects 896. Furthermore, training CAR-T cells enhances their cytotoxicity through AP-1 pathway activation in preclinical studies 897.

Another promising strategy exploits the high collagen content of tumors to direct immunotherapeutic agents, more precisely 898. By conjugating PD-L1 and CTLA-4 inhibitors, as well as cytokines like IL-2, with a collagen-binding motif derived from the von Willebrand factor A3 domain, these agents can be selectively delivered to tumor sites, improving treatment specificity and efficacy 899,900.

On the other hand, chemotherapy remains one of the most used strategies for cancer treatment, primarily targeting malignant cells by disrupting their proliferation mechanisms 901. These mechanisms include inducing DNA damage, inhibiting microtubule, and protein function, blocking DNA synthesis, or interfering with the oncogenic signaling pathway 902. However, chemotherapy is often associated with systemic side effects, such as myelosuppression, due to its impact on blood cell production 903. Beyond its systemic effects on the immune system, it also alters the local tumor immune microenvironment (TIME), with evidence suggesting it can convert immunologically cold tumors into hot ones 904. However, this effect is not universal and may vary between tumor types 905. For instance, in pancreatic cancer, chemotherapy has been observed to promote tumor-supportive immunity by driving the differentiation of monocytes into myeloid-derived suppressive cells (MDSCs) through granulocyte-macrophage colony-stimulating factor (GM-CSF) secretion by cancer cells 906.

Immunogenic cell death (ICD), a key effect of certain cancer therapies such as chemotherapy and radiotherapy, triggers immune activation by releasing damage-associated molecular patterns (DAMPs) and tumor antigens, fostering an anti-tumor immune response 907. However, chemotherapy can also induce changes in the ECM, including fibrosis, which is closely linked to inflammation 908. In some cases, chemotherapy has been shown to promote pro-tumor effects within the TIME 909. For example, paclitaxel treatment can lead CD8^+^ T cells to secrete LOX, facilitating collagen and elastin crosslinking in the lungs and ultimately promoting metastasis 910. Despite these findings, the specific impact of chemotherapeutic and targeted therapies on ECM remodeling and TIME modulation remains largely unexplored, and their effects are likely dependent on the tumor´s pre-existing microenvironment.

A clear example of chemotherapy-induced TIME alterations is seen in the effects of neoadjuvant chemotherapy (NACT) 911. Concurrently, it promotes ECM remodeling, such as the upregulation of collagen VI, which has been associated with chemotherapy resistance and poor prognosis 912. Notably, these ECM alterations are site-dependent, differing between metastatic sites and primary tumors, suggesting that the local TIME is a crucial determinant of ECM remodeling following chemotherapy 913.

Targeting ECM remodeling has shown the potential to enhance chemotherapy responses. For instance, in a breast cancer mouse model. The absence of MMP-9 improved the response to doxorubicin by increasing vascular permeability 914. However, doxorubicin treatment has also been linked to the recruitment of MMP-9-expressing macrophages, which promote chemoresistance 915. These findings highlight the need for further investigation into the relationship between ECM remodeling, immune modulation, and chemoresistance.

As research progresses, integrating ECM-targeting strategies with immunotherapy could open new avenues for more effective and personalized cancer treatments. Altogether, these findings emphasize the complex interplay between chemotherapy, immunotherapy, the immune microenvironment, and ECM remodeling. A deeper understanding of these interactions could lead to novel therapeutic strategies that enhance treatment efficacy, minimize resistance, and improve patient outcomes by co-targeting the ECM and immune components in conjunction with the therapy against cancer. **Table 11 summarizes the main therapeutic agents targeting the ECM in cancer.**

### The use of extracellular vesicles in aging, calcification, and cancer

Extracellular vesicles (EVs) are membrane-enclosed structures released by cells carrying a diverse range of molecular cargo 916. These vesicles play crucial roles in cell-to-cell communication, influencing the fate of recipient cells in both local and distant tissues and serving as pathophysiological biomarkers 917. Traditionally, EVs have been classified into three main types: exosomes, microvesicles, and apoptotic bodies 918.

However, recent research has identified additional subtypes, including autophagic EVs, stress-induced EVs, and matrix vesicles 919.

Autophagy has an important role in EV biogenesis and release. During this process, autophagosomes can fuse with endosomes to form amphiboles, which are subsequently secreted as autophagic EVs 920. Also, processes such as EMT and cancer stemness contribute to EV production. Matrix vesicles and the EMC actively interact with the TME, influencing tumor progression 921.

Extracellular vesicles from healthy cells are being explored as next-generation therapeutics due to their immunogenicity, biocompatibility, and ability to transfer bioactive agents 922. While no ECM-related EV therapeutics are in clinical trials yet, studies suggest their role in modulating the matrix for tissue regeneration 923. EVs are found in bodily fluids like blood, saliva, cerebrospinal fluid, and urine, making them valuable for biomarker discovery in diseases, including cancer 924,925. These biomarkers help in diagnosis, predicting treatment response, and detecting pre-symptomatic conditions. EVs contain RNAs, including miRNA, mRNA, and long noncoding RNAs, which influence recipient cells 926,927. Other RNA types, like circular RNA (circRNA) and PIWI-interacting RNA (piRNA), have also been identified in EVs 928.

#### Aging and EVs

EVs serve as fundamental mediators of intracellular communication, playing both beneficial and detrimental roles throughout the aging process 929. EVs derived from young organisms, healthy tissues, and stem cells often function as positive signaling molecules, promoting cellular repair and regeneration 930. In contrast, EVs from aged or damaged tissues, like tissues with SASP, can propagate cellular senescence and contribute to chronic inflammation 931.

Over time, the balance between healthy and unhealthy vesicles may shift toward an increased presence of aging-promoting EVs, which reinforce tissue damage and accelerate the onset of age-related diseases 932. This phenomenon underscores the need for targeted geroptherapeutic interventions.

Two primary strategies have emerged to counteract the detrimental effects of aging-associated EVs 933. The first approach focuses on eliminating or inhibiting cells that secrete unhealthy EVs 934. The second strategy, which is focused on EV-based regenerative medicine, aims to replace this harmful vesicle with healthy EVs capable of restoring tissue homeostasis and mitigating age-related degeneration 935.

Aging-related diseases currently account for more than 20% of the global disease burden, with cardiovascular disease leading to 30%, followed by cancer with 15%, and pulmonary, musculoskeletal, and neurodegenerative disorders 936. Given the promising therapeutic potential of EV-based treatments in addressing key hallmarks of physiological aging, it is not surprising that these vesicles are being explored as next-generation therapies for age-related conditions 937 **(Table 12).**

#### Calcification and EVs

Apart from being involved in cancer, EVs interact with vascular calcification (**Table 13**). This process refers to the pathological mineralization of the ECM within blood vessels, a process commonly associated with the diseases explained above 938. This phenomenon begins with the transdifferentiation of VSMCs into an osteoblast-like phenotype 939. Under normal physiological conditions, VSMCs release EVs containing calcification inhibitors, which help maintain vascular homeostasis 940. However, under pathological conditions driven by chronic inflammation or abnormal mineral metabolism, osteoblast-like VSMCs produce EVs that resemble matrix vesicles, similar to those involved in bone formation 941.

These pathological EVs have an important role in destabilizing the ECM of blood vessels by promoting calcification in specific regions of the vessel walls 941. This process increases the risk of thrombosis and vessel rupture, leading to severe cardiovascular complications. Recent studies have demonstrated that annexin AI is highly enriched in EVs released by osteoblast-like VSMCs during vascular calcification 941. This protein plays a key role in facilitating the aggregation of EVs within collagen fibrils of the ECM 942. These aggregated EVs serve as nucleation sites for mineralization, which continues in the presence of elevated extracellular Ca^+2^ phosphate (PO_4_^3-^) levels 943. Unlike the mineralization process in bone and cartilage, which occurs within MVs, vascular calcification is driven by the surface phospholipids and annexins of EV aggregates that accumulate with the ECM 944.

#### Cancer and EVs

In TME, EVs facilitate communication between cancer and stroma cells, carrying bioactive molecules like proteins, mRNA, and miRNA 945. Some EVs modulate the ECM, contributing to chemotherapy resistance and metastasis, making them potential therapeutic targets and biomarkers (**Table 14**) 946. Tumor-derived EVs can be detected in the bloodstream, enhancing their clinical relevance 947. A subset of ECM-embedded nanovesicles, matrix-bound vesicles (MBVs), influences cell behavior and may have tumor-specific characteristics 948. MBVs could serve as novel biomarkers, but further research is needed 948. Cell culture models, particularly 3D cultures, help study EVs, as their molecular content closely resembles EVs found in patient tumors 949.

Through different mechanisms, the cancer cell-derived EVs play a central role in shaping the TME, thanks to tumor-derived EVs that contribute to the formation of immunologically cold tumors 950. Furthermore, stromal cells in the TME release EVs that deliver bioactive molecules to cancer cells, promoting chemoresistance, immunotherapy resistance, dormancy stemness, and EMT 951. This reciprocal communication between tumor-associated cells (TACs) and cancer cells via EVs fosters an immunosuppressive and therapy-resistant microenvironment 952.

First, exosomes originate from endosomes through a three-step process: biogenesis, transport, and release 953. They offer a promising drug delivery system due to their biocompatibility, stability, and ability to transport molecules efficiently 954. Unlike conventional drugs, which often have low bioavailability and high toxicity, exosome-based delivery systems can enhance therapeutic efficacy while minimizing side effects 955. They can carry small molecules, proteins, nucleic acids, and gene therapies, improving drug solubility and specificity 956. Exosomes can be loaded with drugs via direct methods, such as incubation, electroporation, liposome transfection, or indirect methods, like engineering donor cells 957,958. Various administration routes exist, but modifying exosomes to extend their circulation time enhances their effectiveness 959. Surface modifications, such as peptide fusions or magnetic therapies, further improve targeting, increasing their potential in personalized cancer therapy 960.

Furthermore, ECM in the TME consists of proteins like collagen, fibronectin, and laminin, supporting cancer progression and immune interactions 961. ECM-rich tumors, such as pancreatic ductal adenocarcinoma, often show poor prognosis due to their dense stroma, which enhances resistance to therapy 962. ECM acts as a barrier against drugs and immune responses while increasing tumor stiffness and promoting mechanotransduction signaling 963,964. Matrix vesicles, including extracellular matrix-bound and matrix-coated vesicles, play key roles in ECM interactions, affecting immune cell function and vascular regulation 965. CAFs and cancer cells produce matrix vesicles, adding niche formation and metastasis 966. ECM-bound molecules like glypican-1 serve as biomarkers, helping in early cancer detection. MMPs on EV surface contribute to cancer progression and tumor niche development 967.

### Regenerative medicine

The molecular crosstalk between cells and the ECM is inherently dynamic and reciprocal. This complex interaction governs essential physiological processes, which are mechanisms vital for maintaining tissue homeostasis and enabling effective wound healing (**Table 15**) 968. In tissue engineering, one of the greatest challenges lies in replicating the native ECM´s biochemical and physical properties 969. These include surface topology, pore size, mechanical strength, biocompatibility, and degradation profile, factors that collectively influence tissue regeneration.

As it is said before, decellularized ECM (dECM) materials have emerged as a promising solution, offering a biologically relevant microenvironment that supports specialized cell function and activates the body´s intrinsic regenerative pathways 970. Through decellularization, immunogenic cellular components are removed, while structural proteins and bioactive macromolecules are largely preserved 971. FDA-approved dECM scaffolds derived from sources like the urinary bladder matrix (UBM) and small intestinal submucosa (SIS) have already demonstrated clinical efficacy in regenerating skin, muscle, and gastrointestinal tissues 972,973.

While early strategies focused on using entire decellularized organs or tissue sheets, issues related to mechanical stability and native tissue mimicry prompted the development of more adaptable forms 974. Researchers have shifted toward micronized dECM particles, which are reconstituted into diverse biomaterial formats, including injectable hydrogels, electrospun scaffolds, and bioprinted constructs 975-977. These newer forms offer improved versatility, enabling customization to match the shape, mechanical properties, and biological demands of specific tissue types.

Overall, dECM-based biomaterials represent a powerful platform for engineering tissue-specific scaffolds that can guide repair and regeneration 978. The most developed applications so far span nine tissue types, including cardiac, neural, cartilage, muscle, liver, and lung, each showing significant progress in scaffold design and regenerative outcomes 979-984. Additional tissues, like the pancreas, kidney, and vocal folds, are beginning to show promise as research expands 985-987.

By tailoring scaffold composition and structure to each tissue´s unique physiological demands and leveraging advanced fabrication technologies, the importance of these strategies is to enable functional tissue regeneration through biomaterials that recapitulate the native ECM 988,989.

## Discussion

The body of work reviewed underscores the intricate interplay between the extracellular matrix (ECM), cellular communication, and the pathogenesis of a wide array of diseases, including cancer, cardiovascular calcification, and age-related degenerative disorders. Collectively, the data reveal that the ECM is far more than a mere structural scaffold; it is a dynamic, bioactive network that influences cellular behavior, modulates tissue homeostasis, and plays a decisive role in disease progression.

In a healthy state, the ECM provides essential biochemical and mechanical cues to maintain tissue integrity and regulate cell proliferation, migration, differentiation, and apoptosis. Its tightly controlled remodeling processes are fundamental for normal development, repair, and regeneration. However, in pathological conditions, these same remodeling processes become dysregulated. Aberrant ECM remodeling (driven by factors such as chronic inflammation, altered intercellular communication, and cellular senescence) can lead to the formation of fibrotic, stiff, and chemically modified matrices. This shift not only supports tumor progression and metastasis. For instance, in cancer, the ECM is remodeled by cancer cells and stromal components like cancer-associated fibroblasts (CAFs), which deposit excess collagen and other matrix proteins. This remodeling increases tissue stiffness, impedes the infiltration of anti-tumor immune cells, and establishes physical and biochemical barriers that reduce the efficacy of therapeutic agents.

Furthermore, the role of cellular senescence in ECM remodeling is multifaced. While a sentence is a natural, protective mechanism (limiting the proliferation of damaged cells and facilitating tissue repair through the senescence-associated secretory phenotype (SASP)), its chronic accumulation can have deleterious effects. Senescent cells secrete a host of pro-inflammatory cytokines, chemokines, growth factors, and proteases, which contribute to a persistent inflammatory state termed inflammaging. The inflammatory milieu not only accelerates the ECM degradation but also fosters an environment conducive to tumor progression. The balance between beneficial and detrimental outcomes of senescence appears to shift unhealthy with age, leading to an accumulation of unhealthy EVs and other secreted factors that reinforce tissue damage and support disease progression.

Also, cellular senescence, a defining feature of aging, establishes a critical nexus between calcification and cancer. As cells enter senescence, they secrete a complex cocktail of pro-inflammatory cytokines, proteases, and growth factors, which disrupt normal ECM remodeling. This altered ECM not only becomes prone to pathological calcification, as seen in VSMC transforming into osteoblasts-like cells but also fosters a tumor-friendly microenvironment. In the context of cancer, senescence-driven ECM changes contribute to increased tissue stiffness and immune evasion, thereby facilitating tumor initiation, progression, and metastasis. Together, these interrelated processes underscore how age-related cellular senescence can promote both calcification and malignancy, highlighting potential targets for therapeutic intervention.

Extracellular vesicles (EVs) further highlight their complex communication networks within tissues. EVs serve as critical mediators of intercellular signaling, transferring bioactive molecules (such as proteins, mRNA, miRNA, and long noncoding RNAs) across cells. In the tumor microenvironment (TME), EVs from cancer cells can modulate the ECM, promote angiogenesis, drive the polarization of immune cells into protumorigenic phenotypes, and even contribute to chemoresistance. Conversely, EVs derived from healthy cells or stem cells have emerged as promising therapeutic agents capable of promoting tissue repair and beneficially modulating the ECM. Their low immunogenicity, high biocompatibility, and intrinsic cargo-carrying capabilities make them attractive for use as drug delivery vehicles and biomarkers for disease progression.

The potential of ECM-targeting and EV-based therapies is especially compelling in the context of regenerative medicine. Stem cell therapies, for instance, not only aim to replace lost or damaged cardiac cells but also seek to modulate the ECM of injured tissues.

Evidence suggests that the engraftment of stem cells, such as mesenchymal stem cells (MSCs), can alter the stiffness and composition of the infarct zone, transforming a rigid car into a more compliant, heterogeneous matrix. This change in ECM properties is thought to facilitate the proliferation and integration of host cells, thereby improving cardiac function. However, challenges remain regarding the optimization of cell delivery, retention, and functional integration, with factors like scaffold design, oxygen diffusion, and biochemical matching playing pivotal roles.

In oncology, the interplay between ECM remodeling and immune surveillance is becoming increasingly apparent. Traditional chemotherapy, while effective in targeting rapidly dividing tumor cells, can also alter the ECM, sometimes exacerbating chemoresistance and fostering an immunosuppressive environment. The use of immune checkpoint inhibitors and adoptive cell therapies has revolutionized cancer treatment, yet their efficacy is often limited by the physical and biochemical barriers imposed by an aberrantly remodeled ECM. Recent studies suggest that a combination of ECM-targeting strategies with immunotherapies could overcome these hurdles. For instance, agents that degrade excessive ECM components, such as hyaluronidase or collagenase, may improve drug penetration and enhance the infiltration and activation of immune cells. Moreover, strategies aimed at normalizing ECM composition (by targeting key enzymes like lysyl oxidase) could help restore a microenvironment more amenable to therapeutic intervention.

Another promising avenue is leveraging the unique gene and protein signatures of the ECM to predict therapeutic responses and guide personalized treatments. The identification of ECM-specific biomarkers, such as particular collagen subtypes or glycoproteins like glypican-A1, provides valuable prognosis information and may inform the selection of targeted therapies. Additionally, computational and big data approaches have enabled the development of ECM gene expression signatures that correlate with treatment sensitivity or resistance, offering a powerful tool for patient stratification.

In summary, the multifaced roles of ECM in regulating cellular behavior, modulating the immune response, and influencing the efficacy of therapeutic interventions are increasingly recognized as critical determinants of disease progression and treatment outcomes. Whether through direct targeting of ECM components, modulation of intercellular communication via EVs, or the integration of advanced regenerative medicine strategies, the emerging evidence underscores the potential for novel therapeutic approaches that address the underlying ECM dysfunctions in a range of diseases. Future research must continue to elucidate the precise mechanism of ECM remodeling and its interplay with cellular senescence, inflammation, and immune therapy. By doing so, it can pave the way for more effective, personalized interventions that not only treat the symptoms of disease but also restore tissue homeostasis and function.

## Conclusion

The body of evidence reviewed establishes the ECM as a central regulator of tissue homeostasis, disease initiation, and therapeutic response. Far from being a passive scaffold, the ECM emerges as a dynamic and bioactive network that integrates mechanical, biochemical, and immunological signals, thereby shaping cellular behavior and influencing the trajectory of health and disease. Its dysregulation, whether through chronic inflammation, senescence, or aberrant remodeling, contributes to fibrosis, calcification, cancer progression, and resistance to therapies, underscoring its dual role as both a mediator of physiological repair and a driver of pathology.

Importantly, the growing underscoring of ECM-cell interactions has opened new therapeutic avenues. Approaches that combine ECM-targeting strategies with immunotherapies or regenerative medicine hold particular promise, as they address not only the tumor or damaged tissue but also the supportive microenvironment that sustains disease. The emerging role of extracellular vesicles as mediators of ECM remodeling and as vehicles for drug delivery adds another layer of therapeutic potential, bridging molecular communication with translational applications.

Looking ahead, the challenge lies in translating these mechanistic insights into clinically effective interventions. This will require refining ECM-targeted therapies, integrating predictive biomarkers, and harnessing big data approaches to better stratify patients and personalize treatments. At the same time, advancing scaffold design, stem cell delivery, and exosome engineering will be essential for regenerative medicine to realize its full potential.

In conclusion, positioning the ECM at the center of therapeutic innovation offers a unique opportunity, not only to combat disease more effectively but also to restore tissue balance and resilience. By continuing to explore its complexities with rigor and creativity, future research can transform the ECM from a barrier to therapy into a gateway for more precise, durable, and patient-tailored interventions.

## Figures and Tables

**Table 1:** Enzymes involved in ECM proteolytic degradation pathways

Enzyme family	Representative members	Substrates	Source cells	Mode of action
MMPs	MMP-1, MMP-2, MMP-9	Collagens I-IV, elastin. Fibronectin	Fibroblasts, neutrophils	Extracellular cleavage
ADAMTS	ADAMTS2, ADAMTS4	Procollagen, aggrecan	Chondrocytes fibroblasts	Secreted proteases
Meprins	Meprin-α, Meprin-β	Collagen IV, fibronectin	Kidney, intestine	Secreted/ membrane-bound
Cathepsins	Cathepsin K, L	Collagens, laminins	Macrophages, osteoclasts	Extracellular or lysosomal
Serine proteases	Plasmin, neutrophil elastase	Fibrin, fibronectin	Neutrophils epithelial cells	Pericellular/ solubleº
Heparanase	-	HSPGs	Endothelial cells, tumors	ECM-bound

**Table 2:** Endogenous inhibitors of ECM proteases

Inhibitor	Target protease	Localization	Functional notes
TIMP-1	MMPs (broad spectrum)	Soluble	Inhibits active MMPs
TIMP-3	MMPs, ADAMs, ADAMTS	ECM-bound	Inhibits sheddases and aggrecanases
Serpins	Serine proteases (e.g., plasmin)	Plasma/ interstitial	Regulate coagulation and fibrinolysis
Cystatins	Cysteine cathepsins	Cytosol/ extracellular	Inhibit lysosomal enzymes
Sulfatases	Modulate HSPG sulfatation	ECM-associated	Influence FGF/VEGF signaling

**Table 3:** All collagen types are divided into different groups that are classified. N.d. (Not defined).

Collagen				
Groups	Collagen types	Molecular assembly	Localization	Function
Fibrillar collagens	I	[α1(I)]_2_ α2(I)] [α1(I)]_3_	Dermis, tendons, and other tissues	Provide tensile strength to tissues; participate in cell migration, adhesion, angiogenesis, tissue development and repair
	II	[α1(II)]_3_		
	III	[α1(III)]_3_		
	V	[α1(V)]_2_ α2(V) [α1(V) α2(V) α3(V)] [α1(V)]_3_		
	XI	[α1(XI) α2(XI) αα3 (XI)]		
	XXIV	[α1 (XXIV)]_3_		
	XXVII	[α1 (XXVII)]_3_		
Network-forming collagens	IV	[α1(IV)_2_ α2(IV)]; α3(IV), α4(IV), α5(IV), α6(IV)	Basement membranes	Formation of complex multimolecular networks from interaction with ECM components; structural support and attachment for cells and tissues; macromolecular filtration barrier
	VII	[α1(VII)]_3_	Descemet´s membrane and vascular sub-endothelial matrices	
	X	[α1(X)]_3_	Hypertrophic zone of growth plate cartilage	
FACITs	IX	[α1(IX) α2(IX) αα3(IX)]	Cartilage associated with type II collagen fibrils	Connecting collagen fibers with other components of the extracellular matrix to maintain tissue integrity
	XII	[α1(XII)]_3_	Collagen type I and II fibrils	
	XIV	[α1(XIV)]_3_	n.d.	
	XVI	[α1(XVI)]_3_	Microfibrils in skin	
	XIX	[α1(XIX)]_3_	N.d.	
	XX	[α1(XX)]_3_	N.d.	
	XXI	[α1(XXI)]_3_	N.d.	
	XXII	[α1(XXII)]_3_	N.d.	
MACITs	XIII	[α1(XIII)]_3_	Cell surface and extracellular matrix	Cell surface receptors; involved in cell adhesion and motility
	XVII	[α1(XVII)]_3_		
	XXIII	[α1(XXIII)]_3_		
	XXV	[α1(XXV)]_3_		
Anchoring fibrils	VII	[α1(VII)]_3_	Lamina densa of the basement membrane	Connects the basement membrane to the underlying stroma to provide structural stability
Beaded-filament-forming-collagens	VI	[α1(VI) α2(VI) αα3(VI)]	Widely expressed in different tissues	Interact with several ECM proteins, HA, PGs, in the tissues in which it is expressed, and type IV collagen in basement membranes
	XXVI	[α1(XXIV)]_3_	N.d.	N.d.
	XXVIII	[α1(XXVIII)]_3_	N.d.	N.d.
Multiplexin	XV	[α1(XV)]_3_	Vascular and epithelial basement membranes in human tissues	Enhances the adhesion of basement membranes to the underlying connective tissue stroma by connecting adjacent collagen fibrils
	XVIII	[α1(XVIII)]_3_		Angiogenesis inhibition

**Table 4:** All collagen types are divided into different groups that are classified. N.d. (Not defined).

Metalloproteases				
Group of Metalloproteases	Subtype	Chromosome	Function	Implication
Collagenase	MMP-1	11	Collagen degradation	Inflammation, autoimmune diseases
	MMP-8	11	Collagen type I, II and III degradation	Scarring and periodontal disease
	MMP-13	11	Type II collagen degradation	Osteoarthritis, pulmonary disease, astrocyte migration, liver fibrosis, metastasis, nasopharyngeal cancer
	MMP-18	12	ECM degradation	Macrophage migration, axonal growth
Gelatinase	MMP-2	16	Localized two-phase collagen degradation	Angiogenesis, tissue repair, inflammation, metastasis, esophageal and lung cancer
	MMP-9	20	ECM degradation	Inflammation, tissue remodeling, esophageal cancer
Stromelysins	MMP-3	11	Degradation of collagen, proteoglycans, elastin and fibronectin	Tissue remodeling, gene regulation, apoptosis, osteoarthritis, atherosclerosis, metastases
	MMP-10	11	N.d.	Pulmonary fibrosis, viral infections, arterial disease, scarring, metastasis, hepatocellular carcinoma
	MMP-11	22	N.d.	Tissue remodeling in embryonic development, scarring, breast cancer, esophageal cancer, oral squamous cell carcinoma
Mathylisins	MMP-7	11	Fas and E-cadherin ligand cleavage	Tissue remodeling, dual involvement in apoptosis, chronic tonsillitis, idiopathic pulmonary fibrosis
	MMP-26	11	Degradation of collagen, fibronectin and gelatin. MMP-9 activation	Tissue remodeling, angiogenesis, tumor invasion, pancreatic adenocarcinoma
Membrane type	MMP-14	14	Modulates MMP-2 activation	Cell migration, tumor invasion, metastasis, aggressive head and neck carcinoma, salivary gland carcinoma
	MMP-15	16	Modulates MMP-2 activation	Placental vasculogenesis, colorectal cancer, supraglottic carcinoma, laryngeal cancer
	MMP-16	8	Modulates activation of MMP-2 and MMP-9	Melanoma, bladder cancer, breast cancer, bronchopulmonary dysplasia
	MMP-17	12	Modulation of growth factors and inflammatory mediators	Breast cancer
	MMP-24	20	Activation by MMP-2, N-cadherin cleavage	Tumorigenesis, neural plasticity, nociceptive signaling, glioblastoma, angiogenesis
	MMP-25	16	Gelatinolytic, MMP-2 activation	Apoptosis, immune response, glioblastomas, colon, urothelial and prostate cancer
Not classified	MMP-12	11	Interferon-α secretion	Immunity, inflammation, cerebral ischemia, head and neck squamous cell carcinoma
	MMP-19	12	N.d.	Angiogenesis, fibrosis, colorectal cancer, lung cancer, gallbladder carcinoma, nasopharyngeal carcinoma
	MMP-20	11	Degradation of amelogenin, processing of aggrecan and oligomeric protein	Enamel biomineralization
	MMP-21	1	Gelatinolytic	Embryogenesis, invasive carcinomas, oral, esophageal and colorectal cancers, Merkel cell carcinoma, pancreatic cancer
	MMP-22	1	N.d.	Tumor suppression
	MMP-23	1	N.d.	Reproduction and breast cancer
	MMP-27	11	N.d.	Endometriotic lesions, menstruation, breast cancer
	MMP-28	17	N.d.	Tissue homeostasis, wound healing, repair, embryo implantation, cardiac remodeling, myocardial infarction, colon cancer, osteoarthritis, rheumatoid arthritis

**Table 5:** Overview of the biological functions of ECM

Function	Key ECM components	Mechanism	Biological impact
Structural support	Collagens I, III, elastin	Tensile strength, elasticity	Tissue architecture
Cell anchorage	Fibronectin, laminins	Integrin-mediated adhesion	Cell polarity, survival
Barrier function	Basement membrane, proteoglycans	Restriction of molecular diffusion	Compartmentalization
Reservoir of growth factors	Perlecan, decorin	Binding and controlled release	Morphogenesis, repair
Signal transduction	All major ECM ligands	Integrin and receptor activation	Proliferation, survival
Migration scaffold	Fibronectin, collagen I	Topography-guided movement	Development, immunity
Pathological modulation	Stiff collagen matrix, matrikines	Remodeling via MMPs, ADAMs	Fibrosis, cancer

**Table 6:** ECM-regulated signaling pathway and cellular outcomes

ECM interaction	Receptor/Pathway	Downstream effect	Context
Integrin-collagen I	FAK/PI3K/MAPK	Cell proliferation	Tumor growth
Lamini-integrin α6β4	YAP/TAZ	Lineage commitment	Strem cell fate
Fibronectin-integrin α5β1	MAPK, Rac1	Cell migration	Wound healing
Detachment from ECM	Loss of integrin signaling	Anoikis	Epithelial turnover
Proteoglycan-TGF-β	SMADs	Fibroblast activation	Fibrosis
Heparan sulfate- FGF/VEGF	RTKs	Angiogenesis	Development, cancer

**Table 7:** ECM changes in physiological vs. Pathological remodeling

Process	ECM composition	Enzymatic activity	Functional outcome
Homeostasis	Balanced ECM synthesis/degradation	Controlled MMP	Tissue integrity
Wound healing	Transient increase in fibronectin, collagen III	MMPs upregulated	Regeneration
Fibrosis	Excess collagen I, reduced elastin	Persistent fibroblast activation	Stiffness, organ failure
Chronic inflammation	Fragmented ECM, increased hyaluronan	MMP overexpression	Tissue disorganization
Cancer	Dense, aligned ECM, rich in fibronectin	MMPs, ADAMs active	Invasion, immune evasion

**Table 8:** The principal ECM changes and shared mechanisms across aging, calcification, and cancer

Process	Key ECM alterations	Shared mechanisms	Pathological outcomes
Aging	Collagen fragmentation, oxydation, glycation, AGEs accumulation Increased ECM stiffness via LOX/LOXL crosslinking Decreased elastin and proteoglycans Accumulation of SASP	Chronic inflammation (inflammaging) ECM stiffening: altered mechanotransduction (YAP/TAZ) SASP-driven MMP activation and ECM degradation	Fibrosis Vascular stiffening and vascular calcification Increased susceptibility to cancer initiation
Calcification	Deposition pf calcium-phosphate crystals in ECM Altered collagen/elastin scaffolds favoring mineralization ECM remodeling via MMPs, ADMTs, and EVs	Senescence and inflammation as triggers of mineralization SASP/EVs promoting osteogenic transformation (RUNX2, Sox9, Klf10) ECM stiffness reinforcing mineralization	Vascular calcification: CVD Osteoarthritis Calcific aortic valve disease Tumor microcalcifications
Cancer	Excessive ECM deposition (collagen I, fibronectin) Crosslinking by LOX/LOXL enzymes: stiffness MMP/ADAMTS-driven ECM degradation releasing bioactive fragments EV accumulation in ECM	Aging-associated SASP fueling tumor-promoting inflammation Calcification (microcalcifications in tumor) as diagnostic/biological marker Shared role of LOX enzymes in fibrosis, calcification, and tumor progression	Tuor growth and metastasis Therapy resistance Chronic inflammation and immune invasion

**Table 9:** Summarized table of the multiple therapist targets against ECM

Therapeutic strategy	Target/ Mechanism	Application/ Benefit	Status/ Evidence
CAR T-cell therapy	Glypican-3	Liver cancer targeting tumor ECM	Ongoing clinical trials
Heparanase inhibitors	ECM degradation enzyme	Multiple myeloma	Preclinical/ clinical studies
PGs, MMPs, CD44/HA signaling modulation	ECM structural proteins and signaling pathways	Tumor growth inhibition, ECM remodeling	Preclinical evidence
VEGF/PDGF inhibitors	Vascular normalization	Improve blood flow and drug distribution	Preclinical/ clinical studies
Collagen-degrading enzymes (MMPs, collagenases)	ECM stiffness reduction	Enhance drug penetration	Preclinical studies
LOX inhibitors	ECM crosslinking and stiffness	Reduce ECM rigidity, facilitate therapy	Preclinical evidence
Biomaterials (collagen, gelatin, fibrin, alginate)	Drug carriers, ECM-mimetic scaffolds	Drug delivery tissue regeneration	Preclinical studies
ECM-derived peptides in biomaterial	Promote vascularization and tissue regeneration	Synergistic regenerative effects	Preclinical studies
Decellularized ECM hydrogels	Structural ECM replacement, progenitor cell support	Tissue regeneration, neovascularization	Preclinical; approaching clinical translation
ECM hydrogels + stem cell/ growth factors	Combination therapy	Enhanced regeneration and repair	Preclinical; increased complexity/ cost

**Table 10:** Main ECM-targeted therapeutic agents and strategies in cardiovascular disease: preclinical and clinical evidence

Therapeutic agent/ Strategy	Target/ Mechanism	Preclinical evidence	Clinical evidence	Notes
Stem cell therapy (autologous/ allogenic CSCs)	Repopulate cardiomyocytes, modify ECM stiffness, promote angiogenesis	Multiple animal models of myocardial infarction; enhanced survival with preconditioning (hypoxia, heat shock, chemical)	Early-phase trials focus on cardiac function; limited long-term ECM data	Variability due to cell type, dose, timing, delivery method
Endogenous progenitor cell stimulation	ECM-based or pharmacological activation of cardiac repair pathways	Preclinical studies show improved vascularization and cell integration	Limited clinical translation	Still under investigation
Decellularized ECM scaffolds	Provide structural and biochemical cues; MMP-sensitive peptides facilitate remodeling	Preclinical tissues engineering models; improve vascularization and hots cell proliferation	Not widely applied clinically yet	Oxygen diffusion limits max scaffold thicknees (near 400 µm)
Omecamtiv mecarbil	Myosin activator; enhances contractility	Preclinical cardiac models	Shown to slightly reduce HF risk in patients with low LVEF	Primarily targets cytoskeleton and contractility
Agrin	ECM proteoglycan: supports neuromuscular junction formation, reduced myocardial injury	Animals models of MI and DCM	Not yet in advanced clinical trials	Demonstrates cardioprotective and anti-fibrotic effects
Spironolactone	Mineralocorticoid receptor antagonist; limits ECM turnover and fibrosis	Preclinical CDV models	Used in patients with congestive heart failure to improve outcomes	Widely clinically applied; acts on ECM remodeling indirectly

**Table 11:** ECM-targeted and ECM-modulating therapeutic strategies in cancer: preclinical and clinical evidence

Therapeutic agent/ Strategy	Target/ Mechanism	Preclinical evidence	Clinical evidence	Notes
Immune checkpoint inhibitors (PD-1, PD-L1, CTL4-A)	Blockade of inhibitory immune pathways; ECM stiffness and TGF-β can impair efficacy	ECM-related genes linked to resistance; ECM stiffness increases PD-L1	Approved for multiple cancers; heterogeneous outcomes	ECM modulation may improve infiltration and response
CAR-T cell therapy	Genetically engineered T cells to recognize tumor antigens	Limited efficacy in solid tumors due to ECM barrier; engineered CAR-T expressing heparanase improve infiltration	Approved in hematologic cancers; limited success in solid tumors	ECM remodeling is key to enhance efficacy
ECM remodeling enzymes (Hyaluronidase)	Degrades hyaluronic acid, reduces stiffness, improves drug penetration	Enhances efficacy of PD-L1 inhibitors, vaccines, and oncolytic viruses in melanoma, breast cancer, glioblastoma	Ongoing preclinical and early translational research	Improves immune infiltration and drug delivery
Oncolytic viruses expressing hyaluronidase	ECM degradation; HA breakdown; activation pf NF-κB in macrophages	Improved CD8+ T cell infiltration and PD-1 blockade efficacy in glioblastoma	Preclinical models	Synergistic with checkpoint inhibitors
TGF-β inhibitors/ modulators	ECM remodeling and immune invasion	Increased CD8+ infiltration improved anti-tumor immunity in colon and breast cancer models	Early clinical trials in solid tumors	Targeting peritumoral collagen improves immunotherapy
Collagen-binding conjugates (PD-L1, CTLA-4 inhibitors, IL-2)	Targeted delivery to collagen-rich tumor ECM	Improved specificity and efficacy in preclinical models	Translational; no large clinical trials yet	Increase immune activation in TME
Chemotherapy (doxorubicin, paclitaxel, NACT)	Disrupt cell proliferation; indirect effects on ECM and TIME	ECM remodeling: collagen VI upregulation, MMP-9 effect on resistance	Standard of care; evidence of ECM-driven resistance	May promote fibrosis or metastasis; context-dependent
MMP-9 modulation	ECM degradation and vascular remodeling	MMP-9 deletion improves doxorubicin response; macrophage-derived MMP-9 linked to resistance	Preclinical studies	Dual role: sensitization vs chemoresistance

**Table 12:** Therapeutic strategies involving EVs in aging and age-related diseases

EV source/ strategy	Role/ mechanism	Impact on aging	Therapeutic potential
EVs from young/ healthy tissues or stem cells	Positive signaling molecules; promote repair and regeneration	Counteract tissue damage, support homeostasis	Regenerative medicine; anti-aging interventions
EVs from aged or SASP-associated tissues	Propagate senescence and chronic inflammation	Accelerate tissue dysfunction and age-related diseases	Target for elimination or inhibition
Strategy 1: Inhibition/ elimination of harmful EV-secreting cells	Prevent release of aging-promoting EVs	Reduces propagation of senescence signaling	Gerotherapeutic approach under study
Strategy 2: EV-based regenerative therapy	Administration of healthy EVs	Restore tissue homeostasis, mitigate degeneration	Next-generation therapies for cardiovascular, cancer, pulmonary, musculoskeletal, neurodegenerative diseases

**Table 13:** Role of EVs in vascular calcification and ECM remodeling

EV source/ strategy	Cargo/ mechanism	Impact on ECM and vessels	Clinical relevance
Normal VSMCs	EVs contain calcification inhibitors	Maintain vascular homeostasis, prevent pathological mineralization	Protective mechanism against vascular disease
Osteoblast-like VSMCs (pathological state)	EVs enriched with annexin 1; resemble bone matrix vesicles	Aggregate in collagen fibrils, act as nucleation sites for mineralization	Promote vascular calcification, thrombosis, vessel rupture
EV aggregation in ECM	Surface phospholipids + annexins enable Ca^2+^/ PO_4_^3-^ deposition	Destabilizes vessel wall structure, drives pathological mineralization	Increases risk of severe cardiovascular complications

**Table 14:** EVs in the tumor microenvironment: roles in ECM remodeling, resistance, and therapeutic applications

EV source/ type	Cargo/ mechanism	Impact on TME and ECM	Clinical/ Therapeutic relevance
Tumor-derived EVs	Proteins, mRNA, miRNA	Modulate ECM; promote chemotherapy resistance and metastasis; shape immunologically cold tumors	Circulating biomarkers; potential therapeutic targets
Stromal cell-derived EVs (CAFS, TACs)	Deliver bioactive molecules	Induce chemoresistance, immunotherapy resistance, EMT, stemness, and tumor dormancy	Foster immunosuppressive, therapy-resistant microenvironment
Matrix bond vesicles (MBVs)	ECM-embedded nanovesicles	Influence cell behavior; niche formation; tumor-specific features	Potential novel biomarkers (need further research)
Exosomes (drug delivery systems)	Small molecules, proteins, nucleic acids, gene therapies	Stable and biocompatible carriers; enable precise molecular transport	Enhances drug solubility, targeting and reduces toxicity, promising for personalized therapy
ECM-derived vesicles (matrix vesicles)	Collagen, fibronectin, laminin interactions; MMPs on EV surface	Promote tumor stiffness, mechanotransduction, immune modulation, vascular regulation	Involved in poor prognosis tumors (pancreatic ductal adenocarcinoma); biomarkers (glypican-1)

**Table 15:** Decellularized extracellular matrix (dECM) biomaterials in tissue engineering and regeneration

Strategy/ material	Key properties	Applications/ Impact	Clinical/ Translational relevance
Native ECM (baseline)	Dynamic crosstalk; surface topology, pore size, mechanical strength, degradation profile	Governs homeostasis, wound healing, and tissue regeneration	Basis for biomimetic scaffold desing
Decellularized ECM (dECM)	Removal of immunogenic components; preservation of structural proteins and bioactive macromolecules	Provides biologically relevant microenvironment; activated intrinsic regenerative pathways	FDA-approved scaffolds (UBM, SIS) for skin muscle, GI tissue
Whole organs/ tissue sheets (early strategies)	Retain native architecture	Limited by poor mechanical stability and incomplete mimicry	Less practical for clinical use
Micronized dECM (next-gen approach)	Reconstituted into hydrogels, electrospun scaffolds, 3D-bioprinted constructs	Versatile formats: customizable to tissue-specific needs	Expands application across multiple tissue types
Tissue-specific dECM scaffolds	Tailored biochemical & biochemical properties	Cardiac, neural, cartilage muscle, liver, lung (well-developed); pancreas, kidney, vocal folds (emerging)	Significant progress in scaffold design and regenerative outcomes
